# Phthalazine-based quaternary ammonium salts: synthesis, biological evaluation and membrane-targeting mechanism against *Staphylococcus aureus*

**DOI:** 10.3389/fmicb.2026.1864148

**Published:** 2026-06-19

**Authors:** Wenwen Liu, Weiling Guo, Dong Xiao, Wang Xin, Baobao Meng, Guifang Zhai, Yan Wang

**Affiliations:** 1 Department of Neurology, Weifang People's Hospital, The First Clinical Hospital of Shandong Second Medical University, Weifang, Shandong, China; 2 Department of Anesthesiology, Weifang People's Hospital, The First Clinical Hospital of Shandong Second Medical University, Weifang, Shandong, China; 3 Clinical Laboratory, Weifang People's Hospital, The First Clinical Hospital of Shandong Second Medical University, Weifang, Shandong, China; 4 Pediatric Surgery Department, Weifang People's Hospital, The First Clinical Hospital of Shandong Second Medical University, Weifang, Shandong, China; 5 Neurosurgery Department, Weifang People's Hospital, The First Clinical Hospital of Shandong Second Medical University, Weifang, Shandong, China

**Keywords:** antibacterial, cell membrane, mechanism of action, phthalazine, quaternary ammonium compounds

## Abstract

*Staphylococcus aureus*, especially methicillin-resistant *S. aureus* (MRSA), poses a severe threat to human health due to the limited efficacy of traditional antibiotics and the rapid emergence of drug resistance. Quaternary ammonium compounds (QACs) are promising antibacterial agents with a membrane-disruptive mechanism that is less prone to inducing resistance. Herein, a series of novel phthalazine-derived QACs (**2a**–**2v**) were designed, synthesized, and evaluated for their antibacterial activities. The structure-activity relationship (SAR) analysis revealed that the length of *N*-alkyl substituent significantly affected antibacterial potency, with compound **2n** emerging as the lead compound. Compound **2n** exhibited broad-spectrum antibacterial activity against *S. aureus*, MRSA, *Streptococcus pneumoniae*, and *Klebsiella pneumoniae*, with MIC values of 2 μg/mL. It showed rapid bactericidal activity, low hemolysis (HC_50_ > 64 μg/mL), minimal resistance evolution, and excellent stability in physiological salts and against proteolytic enzymes, with certain concentration-dependent effects on biofilm. Mechanistic studies revealed that compound **2n** exerts antibacterial activity via triggering bacterial membrane damage, which was verified by multiple phenotypic results, including Gram staining alteration, increased extracellular AKP activity, cell membrane depolarization, intracellular protein and nucleic acid leakage, as well as morphological observations from SEM and DAPI/PI staining. Molecular docking and competitive displacement assays indicated that compound **2n** may bind to bacterial DNA, which is presumed to be a potential auxiliary antibacterial mechanism. These findings suggest phthalazine-derived QACs are promising and compound **2n** has great potential for further development to combat drug-resistant bacterial infections.

## Graphical Abstract

**Figure:**
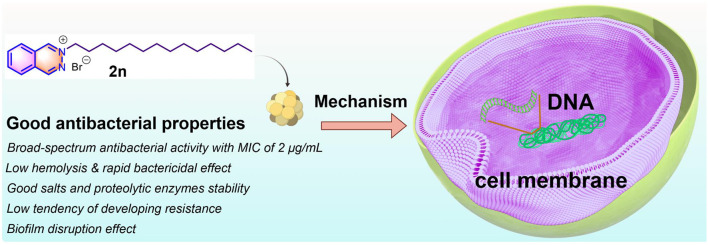

## 1 Introduction

Bacterial infections continue to represent a major public health concern, and *Staphylococcus aureus* stands out as a principal opportunistic microorganism implicated in a range of clinical conditions, such as infections of the respiratory tract, skin and soft tissue structures, and the bloodstream (Parsons et al., 2026). The widespread global prevalence of methicillin-resistant *S. aureus* (MRSA), in particular, has rendered traditional β-lactam antibiotics ineffective (Abebe and Birhanu, 2023). At present, the constrained armamentarium of approved therapeutic options is largely dependent on last-resort agents, including vancomycin and linezolid. Nevertheless, the relentless evolution of drug-resistant variants continues to pose a significant impediment to current treatment paradigms (Elbaiomy et al., 2025). Traditional antibiotics predominantly operate via a “single-target” mechanism of action, readily inducing resistance (Zhang Y. et al., 2024). Moreover, they are usually proved ineffective in eradicating biofilms, frequently leading to chronic infections (Simões, 2011). Given that the evolution of bacterial resistance far outpaces the pace of new drug development, the discovery of lead compounds featuring novel scaffolds, innovative mechanisms of action, and a reduced propensity for inducing resistance has thus become a pressing mandate for the advancement of novel anti-infective strategies (Xue et al., 2026).

Quaternary ammonium compounds (QACs), as a significant class of cationic antibacterial agents, have garnered considerable attention due to their distinctive antibacterial mechanism of action (Dai et al., 2024). The antimicrobial activity is initiated via electrostatic adherence, driven by the attraction of positively charged functional groups to the negatively charged exterior of the bacterial cell envelopes (Li et al., 2026). Concurrently, their hydrophobic alkyl chains insert into the phospholipid bilayer, disrupting membrane integrity and causing bacterial cell death via leakage of cytoplasmic contents (Xu et al., 2025). This physical membrane-disruptive mechanism, targeting the cell membrane, renders QACs less prone to inducing bacterial resistance, while also offering advantages such as rapid bactericidal action and a broad antimicrobial spectrum (Seferyan et al., 2023). In recent years, integrating quaternary ammonium structural units into aromatic heterocyclic frameworks to construct QAC derivatives has emerged as a key strategy in the design of novel antibacterial molecules (Qin et al., 2026). Such hybrid compounds not only retain cationic properties and membrane-targeting capabilities but also have the potential to interact with intracellular biological actions through their rigid scaffolds, thereby engaging a synergistic multiple pharmacological mechanism (Dan et al., 2025). A number of quaternary ammonium salt-based antibacterial agents have been successfully marketed or advanced into clinical stages, typical representatives including cefradine ester and XF-73 (Li et al., 2025).

Phthalazine (2,3-diazanaphthalene), as an important nitrogen-containing heterocyclic scaffold, represents a recurring structural motif across a diverse array of pharmacologically active agents, such as vatalanib and hydralazine (Lakkad et al., 2026). Moreover, the absence of commercial antibacterial drugs based on this scaffold presents a strategic advantage, as it could effectively circumvent cross-resistance (Sakenova et al., 2025). This scaffold is not only cost-effective but also offers excellent structural modifiability, making it an ideal platform for constructing novel QACs. More importantly, the introduction of flexible quaternary ammonium side chains onto the rigid phthalazine core can generate molecular architectures that possess both structural rigidity and membrane-insertion capability. Furthermore, this scaffold may potentially interact with multiple intracellular biological actions, such as DNA replication (Khalifa et al., 2021), statistically reducing the likelihood of resistance emerging through simultaneous genetic mutations.

Based on the preceding analysis, this study designed and synthesized a series of novel QACs based on the phthalazine scaffold. By introducing alkyl chains of varying lengths, we systematically investigated the effect of structural modifications on antibacterial activity, with the ultimate goal of discovering potent new antibacterial lead compounds effective against *Staphylococcus aureus*.

## 2 Materials and methods

### 2.1 Chemistry materials and instruments

All commercially sourced reagents were procured from Aladdin Industrial Co., Ltd. (Shanghai, China) and Energy-Chemical Co., Ltd. (Shanghai, China), and used as received unless otherwise specified. Anhydrous solvents for synthesis and chromatography were supplied by Bodi Chemical Co., Ltd. (Tianjin, China).^1^H and ^13^C nuclear magnetic resonance (NMR) spectra were recorded on a Bruker Avance 400 MHz spectrometer (Rheinstetten, Germany) with chemical shifts reported in parts per million (ppm) relative to residual solvent signals. High-resolution mass spectrometry (HRMS) data were acquired on a Waters SYNAPT G2-Si quadrupole time-of-flight (Q-TOF) mass spectrometer (Manchester, UK). Assessment of final compound content was carried out using a Shimadzu Prominence Plus LC-20A high-performance liquid chromatography (HPLC) system (Kyoto, Japan) fitted with a Shim-pack GIST C18-AQ reversed-phase column (150 mm × 4.6 mm, 5 μm).

### 2.2 Synthesis of compounds 2a−2v

**Scheme 1:**
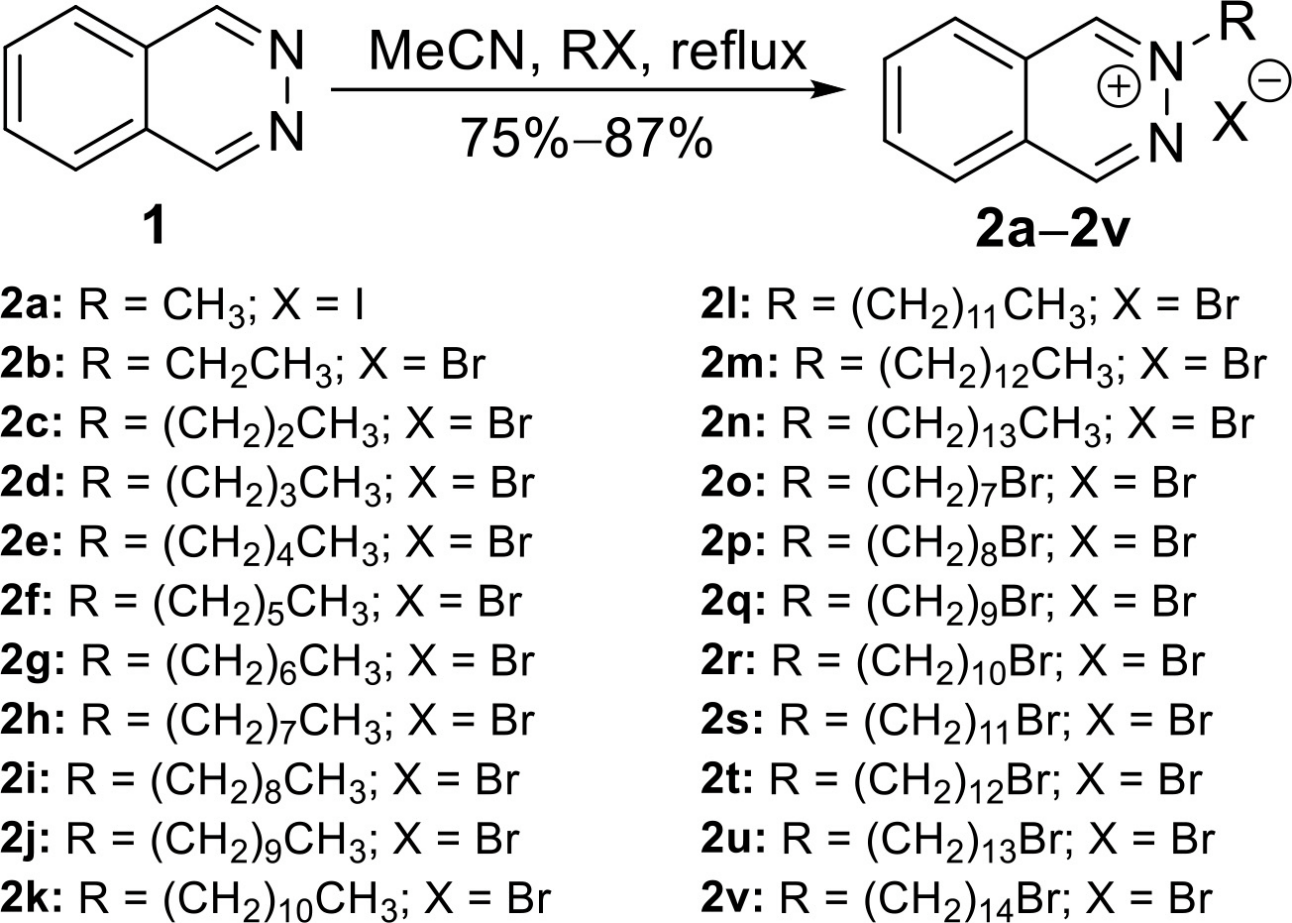
**(A)** Synthesis of the phthalazine-based QACs 2a−2.

As shown in Scheme 1, brominated alkane (1 mmol) was combined with a solution of compound **1** (130 mg, 1 mmol) in anhydrous acetonitrile (10 mL). The reaction mass was brought to reflux temperature until thin-layer chromatography (TLC) indicated full conversion of the starting material **1** (Xiong et al., 2025). Following removal of the solvent in vacuo, the crude residue was subjected to column chromatography with a dichloromethane/methanol gradient elution to yield the desired products **2a**–**2v**. The structural characterization data and spectra can be found in the Supplementary material.

### 2.3 Determination of MIC and MBC

The minimum inhibitory concentration (MIC) values of test compounds **2a**–**2v** against the selected strains of *S. aureus* ATCC 29213, MRSA ATCC 43300, MRSA N315, *Streptococcus pneumoniae* ATCC 49619 and *Klebsiella pneumoniae* CMCC (B) 46117, mainly obtained from the China General Microbiological Culture Collection Center (CGMCC), China Medical Culture Collection Center (CMCC) and Guangdong Microbial Culture Collection Center (GDMCC), were assessed by means of standard broth microdilution methodology (Sui et al., 2025). Briefly, two-fold serial dilutions of each compound were prepared in 96-well plates and inoculated with a standardized bacterial suspension (1 × 10^5^ CFU/mL). Following an 18 h incubation period at 37 °C, the MIC value was defined as the minimal drug concentration at which no visible bacterial proliferation was observed. Among them, vancomycin was used as the positive control. To determine the minimum bactericidal concentration (MBC) value, aliquots taken from wells displaying no macroscopic growth were plated onto nutrient agar and incubated for a further 24 h at 37 °C. The MBC endpoint was designated as the minimum concentration that prevented any subsequent colonial emergence.

### 2.4 Determination of time-growth curve

The mid-log phase bacterial culture was diluted into fresh Mueller-Hinton Broth (MHB) medium (1 × 10^5^ CFU/mL) and grown under 37 °C for 180 rpm. The tested compound **2n** was added at final concentrations of 0, 0.5, 1 and 2 × MIC. Vancomycin (0.5 and 1 × MIC) was included as a positive comparator. The progression of bacterial proliferation was tracked via optical density at 600 nm (OD_600_) measurements acquired on a microplate reader at selected time points (0, 1, 2, 4, 6, 8, 10, 12, 16 and 24 h). The OD_600_ values were plotted against time to construct the growth curve (Xiong et al., 2025).

### 2.5 Determination of time-kill curve

The mid-log phase bacterial culture was diluted to approximately 1 × 10^5^ CFU/mL in fresh medium. The tested compound was added at final concentrations of 1, 2 and 4 × MIC, while vancomycin at 1 and 4 × MIC were used as the positive control and an equal volume of sterile phosphate-buffered saline (PBS) was added to the negative control. All cultures were maintained at 37 °C under constant orbital agitation at 180 rpm. At selected time points (0, 0.5, 1, 1.5, 2 and 4 h), aliquots were collected, serially diluted 10-fold in sterile PBS, and 100 μL of each dilution was spread onto Mueller-Hinton agar (MHA) plates. Colonies were counted after incubation at 37 °C for 24 h. The log_10_ reduction in viable bacterial burden was tracked over time to delineate the corresponding time-kill relationships (Chen W. et al., 2025).

### 2.6 Hemolytic activity assay

Commercial ovine erythrocytes were stored at 4 °C and subsequently rinsed with PBS. Following the final wash, the cells were diluted in PBS to achieve an 8% (*v*/*v*) suspension. In separate centrifuge tubes, 250 μL of compound **2n** at concentrations ranging from 2 to 128 μg/mL were mixed with 250 μL of the erythrocyte suspension. Following a 1 h incubation period at 37 °C, the samples were subjected to centrifugation. The resulting supernatants were carefully collected, and their absorbance was obtained at 540 nm. Control experiments were conducted using sterile 1 × PBS as the negative reference and 1% Triton X-100 as the positive lytic agent. Hemolytic activity was expressed as a percentage and computed in accordance with previously described methods (Gao et al., 2023).

### 2.7 Resistance induction assay

The bacterial suspension was incubated with compound **2n** or norfloxacin at a sub-MIC concentration at 37 °C for a period of 18 h. The culture was then used to determine the updated MIC value, which served as the basis for the subsequent passage. This sequential exposure was sustained over the course of 20 successive passages (Huo et al., 2025). Norfloxacin, a classic fluoroquinolone known to readily induce MRSA resistance, served as the positive control to evaluate whether the test compound promotes or avoids resistance (Xu et al., 2026a,b).

### 2.8 Enzymatic digestion and salt stable assays

The stability of compound **2n** in different concentrations of chymotrypsin and pepsin (1, 10, 100, 1000 and 10,000 μg/mL) was evaluated by RP-HPLC (Zhong et al., 2025). Among them, protease need to be co-incubated with compound **2n** (100 μg/mL) at 37 °C for 6 h in advance, whose activity was quenched by ice acetonitrile. The sample for analysis was subsequently obtained by centrifugation at 12,000 rpm and filtration by microfiltration membrane (0.22 μm). Chromatographic separation was achieved using a gradient of 5–85% acetonitrile in water (containing 0.1% formic acid) over 20 min at a flow rate of 1 mL/min, with UV detection set at 310 nm.

In addition, salt sensitivity was assessed by comparing the MIC fold change of compound **2n** against *S. aureus* ATCC 29213 in the presence of different saline ions (150 mM NaCl, 4.5 mM KCl, 2 mM CaCl_2_, 8 μM ZnCl_2_, and 1 mM MgCl_2_) relative to that in MHB medium (Guo et al., 2023). Notably, the concentrations of salt ions were selected by referring to the physiological ion concentration in the human microenvironment (Zhang J. et al., 2025).

### 2.9 Biofilm disruption assay

To establish an *in vitro* preformed biofilm model of *S. aureus* ATCC 29213, 200 μL of bacterial suspension (1 × 10^8^ CFU/mL) in TSBG (TSB medium containing 0.5% glucose) was dispensed into a 96-well plate and cultured at 37 °C for a period of 24 h. Once the biofilms had been established, the wells were gently washed with PBS and subsequently incubated with 200 μL of fresh culture medium supplemented with compound **2n** at final concentrations of 0, 2, 4, 8, and 16 μg/mL. The plates were then incubated for an additional 24 h. Following another PBS wash, the biofilms were fixed with methanol and stained with 0.1% crystal violet (CV). Unbound dye was removed, and 200 μL of 33% acetic acid was added to each well. The OD_570_ value was subsequently measured to assess changes in biofilm biomass. Meanwhile, for the rinsed biofilms, alterations in viable bacterial colonies were evaluated using the standard plate count method (Dai et al., 2024). This experiment was designed to evaluate the eradication effect on preformed mature *S. aureus* biofilms, rather than biofilm formation inhibition. Vancomycin was set as the standard antibacterial control under the same experimental conditions for parallel comparison (Yu et al., 2021).

### 2.10 Scanning electron microscope (SEM) assay

The bacterial suspension (200 mL) in the logarithmic growth phase was diluted to 1 × 10^8^ CFU/mL in MHB medium containing compound **2n** (2 μg/mL). Bacterial cultures were propagated at 37 °C for 4 h under constant agitation (180 rpm). The cells were subsequently harvested by centrifugation at 4,000 rpm for 5 min and rinsed twice with PBS. The washed bacterial pellets were fixed in a 2.5% (*v*/*v*) glutaraldehyde solution, which were then delivered to the company of Research Dog Instrument Test Platform for sampling and taking photos. MHB without compound **2n** served as the blank control (Li et al., 2026).

### 2.11 Gram staining assay

Mid-logarithmic phase cultures were normalized to 1 × 10^8^ CFU/mL using fresh PBS. Subsequently, compound **2n** with various concentrations of 0, 1, 2 and 4 × MIC was added and cultured at 37 °C with shaking at 180 rpm for a period of 4 h. Bacterial smears were prepared on glass slides, dried, and fixed by heat. Gram staining involved sequential treatment with CV (1 min), water wash, Gram's iodine (1 min), water wash, decolorizer (1 min), and a final water wash followed by blotting. Counterstaining was performed with safranin for 1 min, after which the slides were rinsed with water. Observation was carried out under a light microscope using a 100 × oil immersion objective (Zhang L. et al., 2024).

### 2.12 Determination of alkaline phosphatase (AKP) activity

The bacterial culture in mid-logarithmic growth phase was adjusted to a density of roughly 1 × 10^8^ CFU/mL using sterile PBS. Subsequently, varying concentrations of compound **2n** (0, 2, 4, 8, and 16 μg/mL) were introduced, and the mixtures were incubated at 37 °C (180 rpm, 4 h). Following centrifugation, the resulting supernatants were harvested, and the AKP activity was measured following the protocol provided with the commercial AKP assay kits (Tian et al., 2026), procured from Nanjing Jiancheng Bioengineering Institute (Nanjing, China).

### 2.13 Membrane depolarization assay

The bacterial culture in mid-logarithmic growth phase was pelleted via centrifugation at 4,000 rpm for 5 min. After washing, the cells were re-suspended in a mixed solution consisting of 5 mM HEPES buffer, 5 mM glucose, and 100 mM KCl at a volume ratio of 1:1:1. Aliquots of the bacterial suspension (1 × 10^8^ CFU/mL, 150 μL) were transferred into wells, followed by the addition of 50 μL of the fluorescent probe DiSC35 at a concentration of 10 μM. The mixture was allowed to pre-incubate for 30 min. Fluorescence readings were then taken at excitation and emission wavelengths of 622 nm and 670 nm, respectively, at 1-min intervals until the signal reached a steady state. Thereafter, varying concentrations of compound **2n** (0, 2, 4, 8, and 16 μg/mL) along with the positive control (1% Triton X-100) were introduced, and fluorescence intensity was recorded every 1 min (Xiong et al., 2025).

### 2.14 Protein and nucleic acid leakage assays

Serially diluted concentrations of compound **2n** (0, 2, 4, 8, and 16 μg/mL) were subjected into the logarithmically growing bacterial suspension (1 × 10^8^ CFU/mL). After incubation at 37 °C for 4 h with shaking at 180 rpm, centrifugation at 4,000 rpm for 5 min was employed to clarify the suspension, after which the resulting supernatant was carefully harvested. The leaked proteins were then evaluated by measuring the OD_280_ values, while the leaked nucleic acids were assessed by OD_260_ values (Li et al., 2026).

### 2.15 Double staining assay

A logarithmically growing *S. aureus* ATCC 29213 suspensions adjusted to 1 × 10^8^ CFU/mL was treated with compound **2n** at a fixed concentration of 8 μg/mL. After a 4 h incubation at 37 °C, the cells were rinsed with 1 × PBS. The samples were then sequentially stained with 4′,6-diamidino-2-phenylindole (DAPI, 10 μg/mL) and propidium iodide (PI, 5 μg/mL), followed by a 10 min incubation in the dark. A parallel set of samples treated with PBS alone served as the untreated reference. Finally, the stained bacterial cells were visualized under a fluorescence microscope (Dan et al., 2025).

### 2.16 Molecular docking assay

The three-dimensional structure of DNA was retrieved from the RCSB Protein Data Bank under the accession code 454D. The molecular model of compound **2n** was built within the Sybyl-X 2.0 software environment. Subsequent molecular docking studies were carried out via the Surflex-Dock engine integrated within the aforementioned software suite (Dan et al., 2025).

### 2.17 Competitive displacement assays

Calf thymus DNA (ct-DNA) is universally recognized as an ideal alternative model to simulate bacterial double-stranded DNA *in vitro*, which can effectively reflect the binding, intercalation and groove-binding behaviors of antibacterial small molecules with DNA (Liu et al., 2024). The ct-DNA–EB or ct-DNA–AO complex was prepared by adding 5 μM EB or 5 μM AO into 60 μM ct-DNA in Tris-HCl buffer (pH 7.4), respectively. After mixing the solutions of **2n** (30, 50, 70, 90, 110, 130, 150 μM) and the complex system, the final mixture was allowed to equilibrate for 10 min at room temperature. Fluorescence measurements for the EB displacement assay were conducted using an excitation wavelength of 497 nm, while emission data were collected across the 520–800 nm spectral window. Similarly, AO displacement assay was performed with an excitation wavelength of 500 nm and an emission range of 460–700 nm. Notably, compound **2n** remained non-fluorescent under the assay conditions, displaying negligible emission both in the free state and in the presence of ct-DNA at room temperature (Amir et al., 2025).

### 2.18 Statistical analysis

Each assay was performed a minimum of three times independently. The resulting data are presented as the mean ± standard deviation (SD), and statistical processing was carried out using GraphPad Prism version 8.0. To assess statistical significance, either Student's *t*-test or one-way analysis of variance (ANOVA) was applied, with significance levels denoted as follows: ^*^P < 0.05, ^**^P < 0.01, ^***^P < 0.001, and ^****^P < 0.0001.

## 3 Results and discussion

### 3.1 Chemistry

In the pursuit of developing novel antibacterial agents with optimized membrane-interaction properties, we designed a series of phthalazine-derived QACs (Scheme 1, **2a**–**2v**) by conducting the relevant literatures of cationic amphiphiles (Dan et al., 2022). The core design rationale centers on the critical role of hydrophobic alkyl chain length in modulating the amphiphilic balance of QACs. The positively charged quaternary nitrogen provides electrostatic affinity for the negatively charged bacterial cell membrane, while the alkyl tail governs hydrophobic insertion into the lipid bilayer, a key step for membrane disruption and subsequent bactericidal activity. By systematically varying the length of the *N*-alkyl substituent, from methyl to tetradecyl, including ω-bromoalkyl chains for further functionalization, we aimed to fine-tune the lipophilicity, membrane permeability, and ultimately the antibacterial potency of the compounds, while retaining the phthalazine heterocyclic scaffold as a rigid, pharmacologically active core.

The synthetic route to the target phthalazinium salts is outlined in Scheme 1. Commercially available phthalazine **1** was subjected to a direct *N*-alkylation reaction with a panel of alkyl halides in refluxing acetonitrile. This nucleophilic substitution reaction proceeds via attack of the nucleophilic phthalazine nitrogen on the electrophilic alkyl halide, cleanly furnishing the corresponding *N*-alkylphthalazinium halides **2a**–**2v** in good yields (75%−87%). The series encompasses linear alkyl chains of increasing length to evaluate the impact of hydrophobicity on antibacterial activity. This modular synthetic approach enables systematic structure-activity relationship (SAR) studies to identify the optimal chain length for maximum antibacterial efficacy, laying the foundation for subsequent biological evaluation.

All synthesized phthalazinium QACs **2a**–**2v** were fully characterized by standard spectroscopic methods to confirm their chemical structures. ^1^H NMR and ^13^C NMR spectroscopy were employed to assign the proton and carbon environments of each compound, verifying the successful *N*-alkylation of the phthalazine core and the integrity of the alkyl chain substituents. HRMS was used to confirm the molecular weight of each target compound, consistent with the expected quaternary ammonium cation structure. All spectroscopic data are in full agreement with the proposed structures, confirming the correctness of the synthesized compounds.

### 3.2 Antibacterial activity and SAR

The *in vitro* antibacterial efficacy of compounds **2a**–**2v** was assessed against a panel of clinically relevant pathogens, with MIC values summarized in Table 1. A clear, length-dependent SAR was observed across the series, governed by the hydrophobic balance of the *N*-alkyl substituent. For linear alkyl chain derivatives (**2a**–**2n**), short-chain analogs (C_1_-C_6_, **2a**–**2f**) were universally inactive (MIC > 128 μg/mL), while potency increased progressively with chain extension. Activity emerged at C_7_-C_8_ (**2g**–**2i**) and improved dramatically with longer chains, with the C_14_ derivative (**2n**) emerging as the lead compound. Compound **2n** exhibited exceptional broad-spectrum activity against all tested strains, with MIC values of 2 μg/mL against *S. aureus*, MRSA, *S. pneumoniae*, and *K. pneumoniae*. For ω-bromoalkyl chain analogs (**2o**–**2v**), a similar length-dependent trend was observed, with activity rising as the alkyl spacer extended from C_7_ to C_14_. While terminal bromination slightly reduced potency relative to linear alkyl counterparts, C_12_-C_14_ derivatives (**2t**–**2v**) retained strong activity against MRSA and *S. pneumoniae*. Notably, multiple analogs (**2l**–**2n**, **2t**–**2v**) displayed potent activity against MRSA strains (MIC = 2–4 μg/mL). Critically, lead compound **2n** demonstrated excellent activity against both *S. pneumoniae* and *K. pneumoniae*, key etiological agents of bacterial pneumonia, highlighting the scaffold's potential for treating respiratory infections. Subsequent antibacterial property and mechanistic investigations focused on *S. aureus* ATCC 29213 to elucidate the mode of action of this class of cationic amphiphiles.

**Table 1:** MIC values of compounds **2a**–**2v** against bacteria (μg/mL).

Compounds	R	X	*S. aureus* ATCC 29213	MRSA ATCC 43300	MRSA N315	*S. pneumoniae* ATCC49619	*K. Pneumoniae* CMCC (B) 46117
**2a**	CH_3_	I	>128	>128	>128	>128	>128
**2b**	CH_2_CH_3_	Br	>128	>128	>128	>128	>128
**2c**	(CH_2_)_2_CH_3_	Br	>128	>128	>128	>128	>128
**2d**	(CH_2_)_3_CH_3_	Br	>128	>128	>128	>128	>128
**2e**	(CH_2_)_4_CH_3_	Br	>128	>128	>128	>128	>128
**2f**	(CH_2_)_5_CH_3_	Br	>128	>128	>128	>128	>128
**2g**	(CH_2_)_6_CH_3_	Br	>128	>128	>128	128	>128
**2h**	(CH_2_)_7_CH_3_	Br	>128	>128	>128	64	>128
**2i**	(CH_2_)_8_CH_3_	Br	64	64	>128	16	64
**2j**	(CH_2_)_9_CH_3_	Br	16	16	16	8	16
**2k**	(CH_2_)_10_CH_3_	Br	8	4	16	8	4
**2l**	(CH_2_)_11_CH_3_	Br	4	4	4	4	4
**2m**	(CH_2_)_12_CH_3_	Br	2	4	4	4	4
**2n**	(CH_2_)_13_CH_3_	Br	2	2	2	2	2
**2o**	(CH_2_)_7_Br	Br	>128	>128	>128	>128	>128
**2p**	(CH_2_)_8_Br	Br	128	>128	128	>128	>128
**2q**	(CH_2_)_9_Br	Br	64	64	64	64	64
**2r**	(CH_2_)_10_Br	Br	32	64	64	64	64
**2s**	(CH_2_)_11_Br	Br	8	8	8	8	8
**2t**	(CH_2_)_12_Br	Br	4	4	4	4	4
**2u**	(CH_2_)_13_Br	Br	2	4	4	4	4
**2v**	(CH_2_)_14_Br	Br	2	4	4	4	2
Vancomycin	–	–	1	1	1	0.5	>128

### 3.3 Growth and killing kinetics assessment

**Figure 1:**
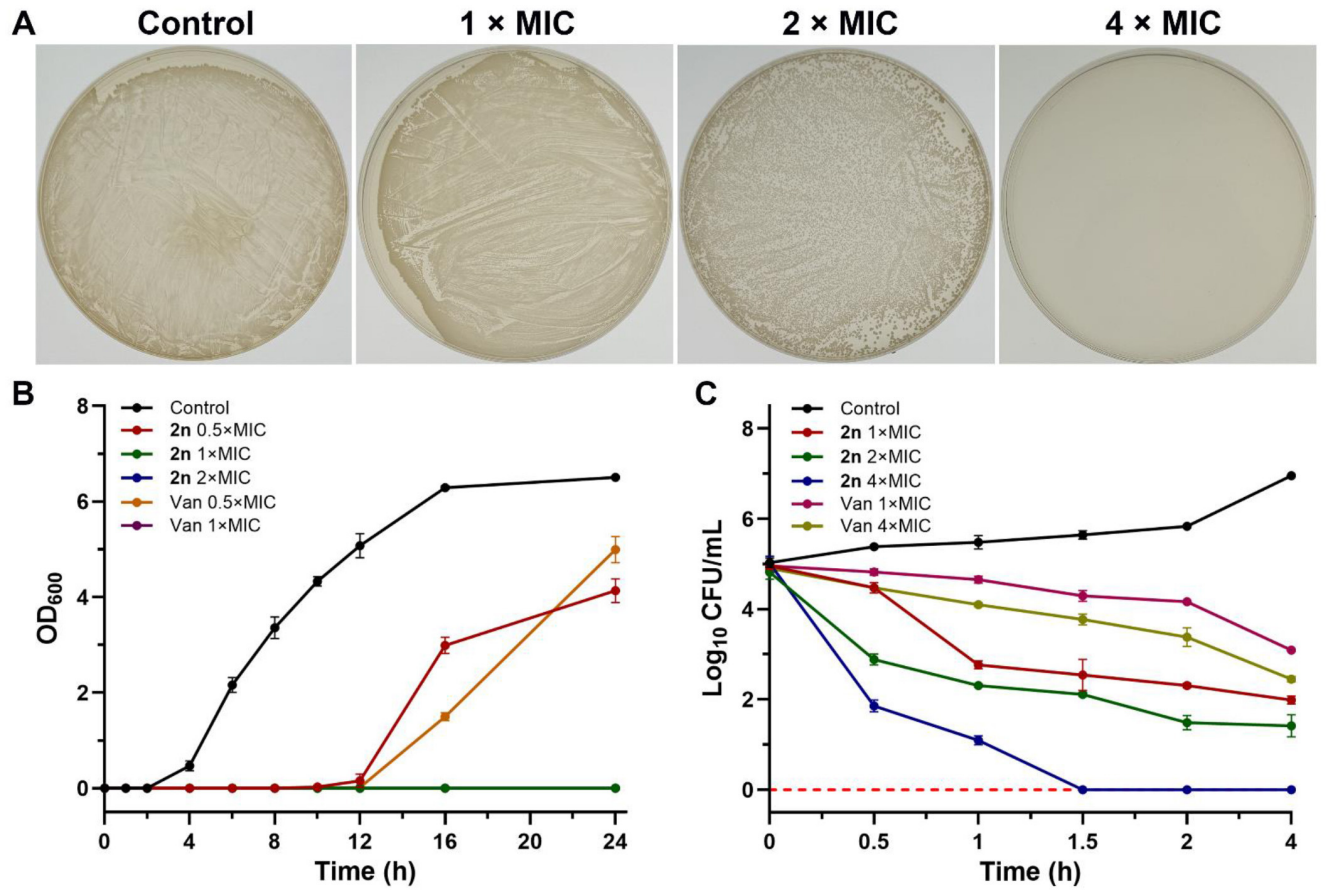
**(A)** Representative agar plates of *S. aureus* growth after 24 h exposure to **2n** at 0 (control), 1 ×, 2 ×, and 4 × MIC; **(B)** Time-growth curves of *S. aureus* treated with **2n** (0, 0.5, 1 and 2 × MIC) and vancomycin (0.5 and 1 × MIC) over 24 h; **(C)** Time-kill kinetics of *S. aureus* treated with **2n** (1, 2 and 4 × MIC) and vancomycin (1 and 4 × MIC) over 4 h.

Bactericidal kinetics and growth inhibition are key indicators for evaluating antibacterial performance (Chen W. et al., 2025). As depicted in Figure 1A, the MBC of lead compound **2n** against *S. aureus* ATCC 29213 was determined as 4 × MIC, at which complete bacterial eradication was achieved, demonstrating a distinct bactericidal action. In time-growth curves (Figure 1B), 0.5 × MIC of **2n** significantly delayed bacterial proliferation and prolonged the lag phase, whereas concentrations of 1 × MIC and above completely inhibited *S. aureus* growth over 24 h. Time-kill assays further revealed that **2n** exerted rapid, concentration-dependent bactericidal activity (Figure 1C). At 4 × MIC, it achieved full bacterial elimination within 1.5 h. And at 2 × MIC, it induced a 3.4 log_10_ CFU/mL reduction in viable bacterial counts within 4 h. Notably, compound **2n** exhibited faster and more potent bactericidal activity than vancomycin at equivalent concentrations, with a markedly higher rate of bacterial clearance. Collectively, these results confirm that **2n** acts as a potent and fast-acting bactericidal agent against *S. aureus*, with both growth-retarding effects at sub-MIC levels and strong bactericidal efficacy at effective concentrations.

### 3.4 Hemolytic toxicity

**Figure 2:**
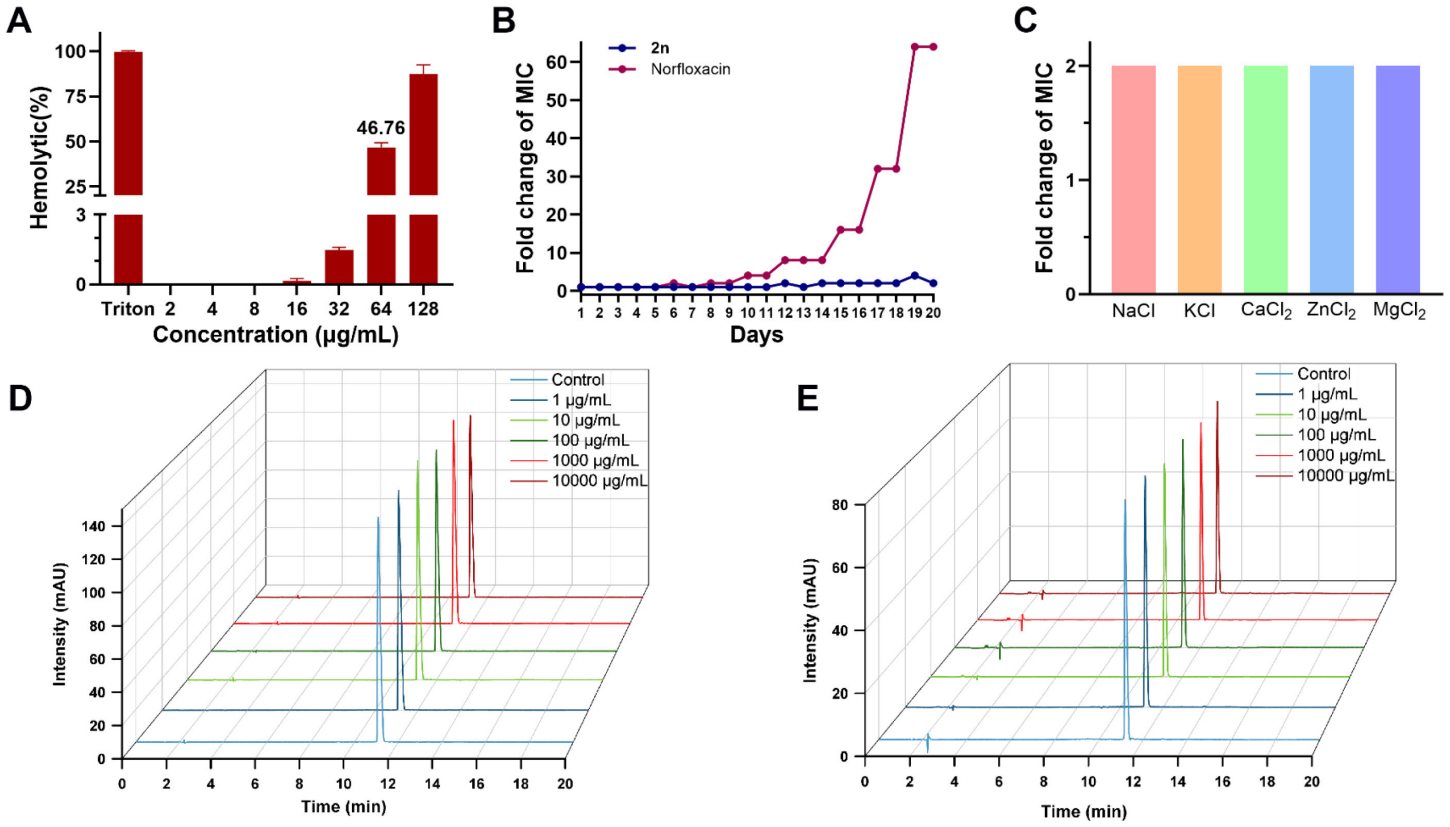
**(A)** Hemolytic activity of **2n** against sheep RBCs at concentrations ranging from 2 to 128 μg/mL, with 1% Triton X-100 as the positive control; **(B)** Resistance development of *S. aureus* ATCC 29213 during 20 serial passages in the presence of sub-MIC concentrations of **2n** and norfloxacin; **(C)** Effect of physiological salts (NaCl, KCl, CaCl2, ZnCl2, MgCl2) on the antibacterial activity of **2n** against *S. aureus* ATCC 29213; (**D** and **E**) HPLC chromatograms showing the stability of **2n** after incubation with pepsin **(D)** and chymotrypsin **(E)** at 37 °C for 6 h.

Hemolytic toxicity is a key safety concern for cationic QACs (Li et al., 2026), as their membrane-disrupting mechanism can cause non-specific lysis of eukaryotic red blood cells (RBCs), making the selectivity index (SI = HC_50_/MIC) a critical metric for clinical potential. As shown in Figure 2A, lead compound **2n** exhibited negligible hemolysis at concentrations ≤ 32 μg/mL, with a hemolytic rate of 46.76% at 64 μg/mL, confirming an HC_50_ value above 64 μg/mL. Given its MIC of 2 μg/mL against tested bacteria, compound **2n** achieves a high selectivity index of 32, indicating a wide therapeutic window. This favorable profile stems from the optimized C_14_ alkyl chain, which balances potent antibacterial membrane disruption against bacteria with minimal non-specific lysis of mammalian cells, supporting its safety as a promising antibacterial candidate. Nevertheless, the current work only provides hemolytic toxicity data, which cannot fully confirm the cellular selectivity and therapeutic potential of compound **2n**. More comprehensive cytotoxicity and safety evaluation should be performed in future studies to supplement the pharmacological application basis.

### 3.5 Resistance evolution study

Antibiotic resistance is a critical clinical threat, making resistance propensity a key translational metric (Huo et al., 2025). As shown in Figure 2B, serial passaging of *S. aureus* ATCC 29213 under sub-MIC pressure revealed distinct resistance profiles. Lead compound **2n** exhibited minimal resistance evolution, with only a 2-fold increase in MIC after 20 passages, reflecting excellent genetic stability. In stark contrast, norfloxacin induced rapid, robust resistance, with its MIC rising 64-fold over the same period. This confirms **2n** strongly suppresses resistance development in *S. aureus*. Unlike target-specific antibiotics, compound **2n** disrupts bacterial membranes via a physical mechanism, highlighting its strong potential against resistant infections.

### 3.6 Stability study

The stability of antibacterial agents in physiological environments is a critical prerequisite for *in vivo* therapeutic efficacy, directly impacting their clinical translational potential (Zhong et al., 2025). As illustrated in Figure 2C, lead compound **2n** exhibited exceptional stability against physiological salt interference in the presence of NaCl, KCl, CaCl_2_, ZnCl_2_, and MgCl_2_. Its MIC against *S. aureus* only increased by a maximum of 2-fold, with no significant loss of antibacterial activity. This confirms that compound **2n** maintains potent bactericidal efficacy even under physiological ionic conditions, a key advantage for *in vivo* applications.

For enzymatic stability, HPLC chromatograms (Figures 2D, E) demonstrated that compound **2n** remained fully intact after incubation with pepsin (gastric) and chymotrypsin (intestinal) for 6 h, with no detectable degradation products and no significant changes in peak area across all tested concentrations. This robust resistance to proteolytic degradation, combined with excellent salt stability, addresses a major limitation of many conventional antimicrobials and peptide-based agents. Collectively, these results validate the high stability of compound **2n** in physiological and digestive environments, supporting its suitability for both systemic and oral administration, and further reinforcing its promise as a clinically viable antibacterial candidate.

### 3.7 Mature biofilm disruption activity

**Figure 3:**
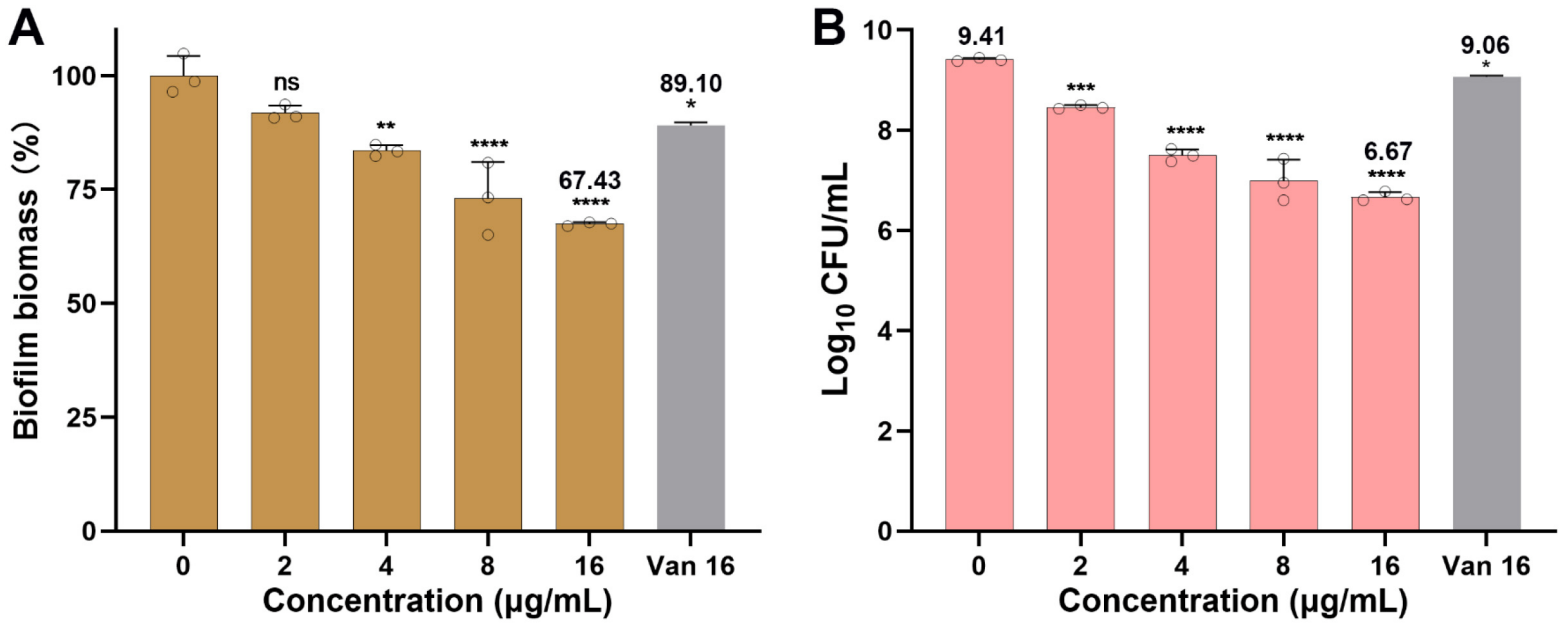
**(A)** Quantification of biofilm biomass by CV staining after 24 h treatment with **2n** at 0, 2, 4, 8, and 16 μg/mL; **(B)** Enumeration of viable bacteria within mature biofilms following **2n** treatment.

Mature bacterial biofilms are a critical driver of antibiotic tolerance and persistent infections, as the extracellular matrix shields embedded bacteria from antimicrobials (Dai et al., 2024). Compound **2n** exhibited concentration-dependent eradication activity against mature *S. aureus* biofilms. CV staining confirmed **2n** significantly reduced biofilm biomass in a dose-dependent manner, with 32.57% clearance of mature biofilms at 16 μg/mL (Figure 3A). Viable count assays further demonstrated a marked reduction in biofilm-embedded bacteria, achieving a 2.74 log_10_ CFU/mL decrease at 16 μg/mL (Figure 3B). Notably, a discrepancy was observed between the two assays. CV staining showed only moderate reduction in total biofilm biomass, while viable counts revealed a pronounced decrease in culturable bacteria. As we all known, CV labels the entire biofilm matrix including extracellular polysaccharides and dead cell debris, whereas viable counts specifically quantify metabolically active, embedded bacteria. The residual biomass signal likely reflects partial retention of the biofilm's structural components, even as the majority of viable bacteria were eliminated. These results suggest compound **2n** might eliminate protected bacteria at high concentrations, even when full structural disruption of the matrix is incomplete. In addition, the standard clinical antibacterial agent vancomycin showed weak eradication activity against mature *S. aureus* biofilms at high concentration (16 μg/mL), which is consistent with previous literature reports (Chen S. et al., 2025; Yu et al., 2021).

### 3.8 Bacteria morphology analysis

**Figure 4:**
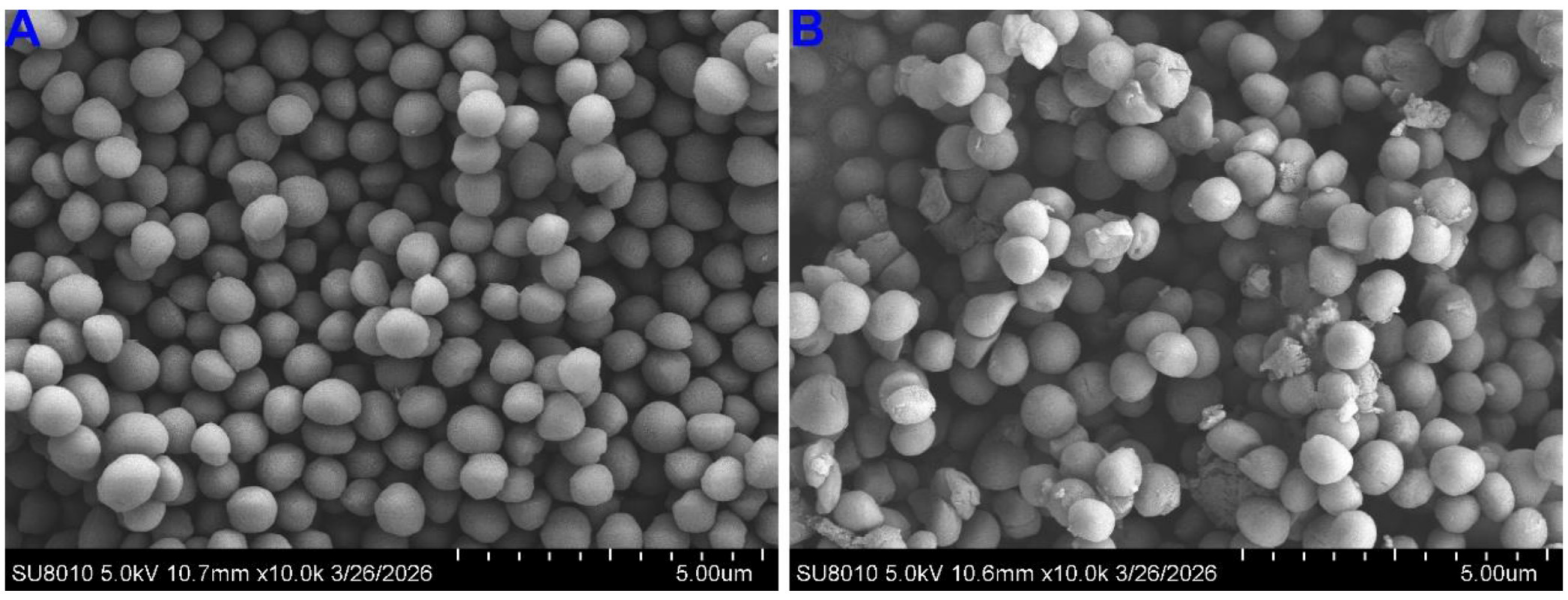
SEM analysis of *S. aureus* ATCC 29213 morphologies after treatment with compound **2n**. **(A)** Control group; **(B)** Treatment with **2n** at 1 × MIC for 4 h. Scale bar is 5 μm.

SEM was employed to visualize the morphological effects of compound **2n** on *S. aureus* ATCC 29213 (Xu et al., 2026b). As shown in Figure 4A, untreated control cells exhibited a typical, intact spherical morphology with smooth, regular surfaces and intact cell walls as well as membranes. In contrast, treatment with **2n** at 1 × MIC for 4 h induced severe morphological damage, including cell shrinkage, membrane rupture, cytoplasmic leakage, and partial cell lysis (Figure 4B). These findings confirm compound **2n** disrupts bacterial membranes, consistent with its cationic antibacterial mechanism.

### 3.9 Effect on cell membrane

**Figure 5:**
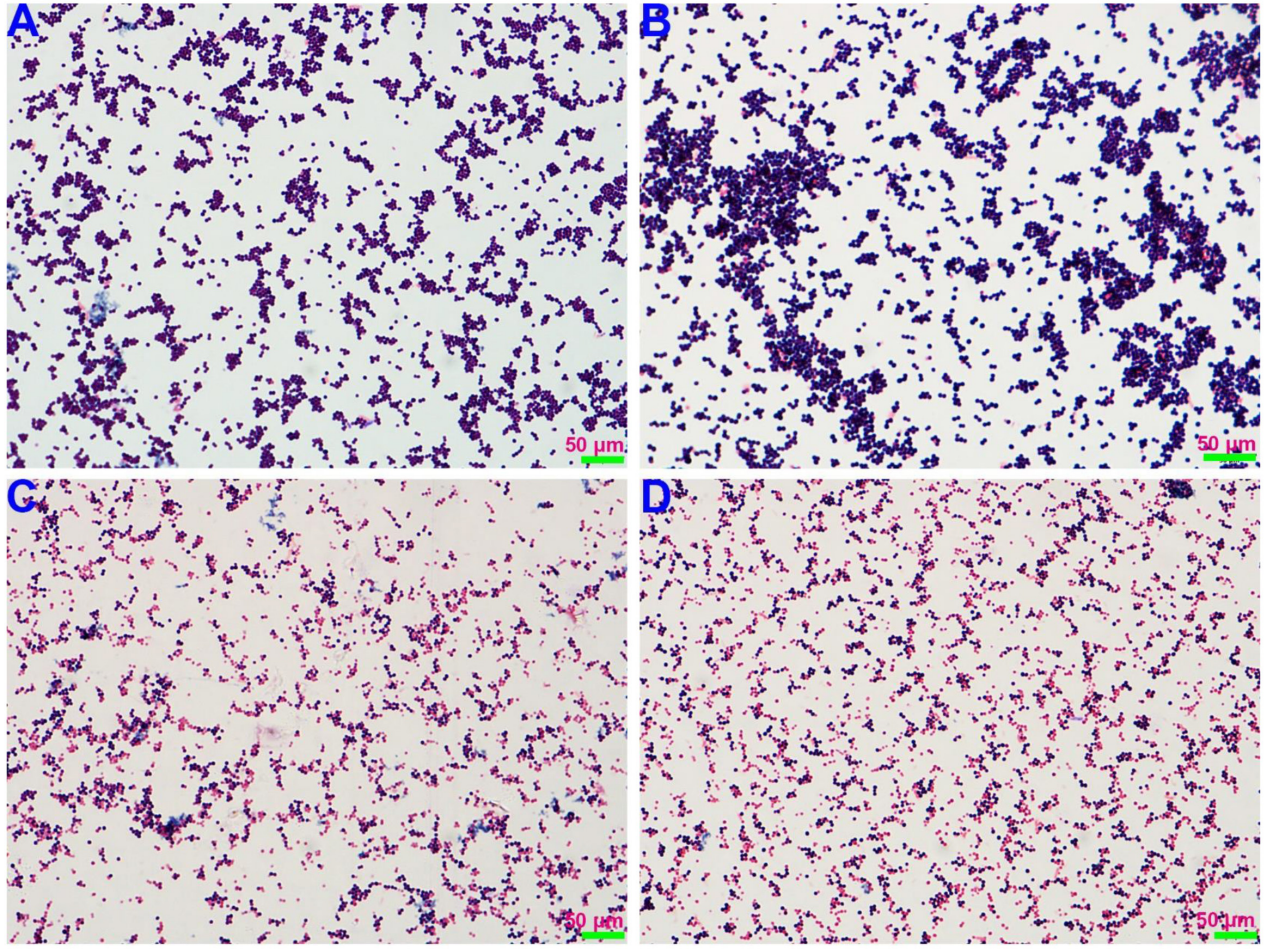
Gram staining analysis of *S. aureus* ATCC 29213 after treatment with compound **2n**. **(A)** Control group, 0 × MIC; **(B)** 1 × MIC treatment group; **(C)** 2 × MIC treatment group; **(D)** 4 × MIC treatment group. Scale bar is 50 μm.

Gram staining was employed to evaluate the influence of compound **2n** on the *S. aureus* ATCC 29213 (Figure 5). Untreated control cells exhibited uniform, deep purple Gram-positive staining, characteristic of intact, functional cell envelopes. In contrast, compound **2n** treatment induced a concentration-dependent impairment of cell envelopes. Partial decolorization was evident at 1 × MIC, with progressively extensive pink counterstaining and severe structural disruption at 2 × and 4 × MIC.

**Figure 6:**
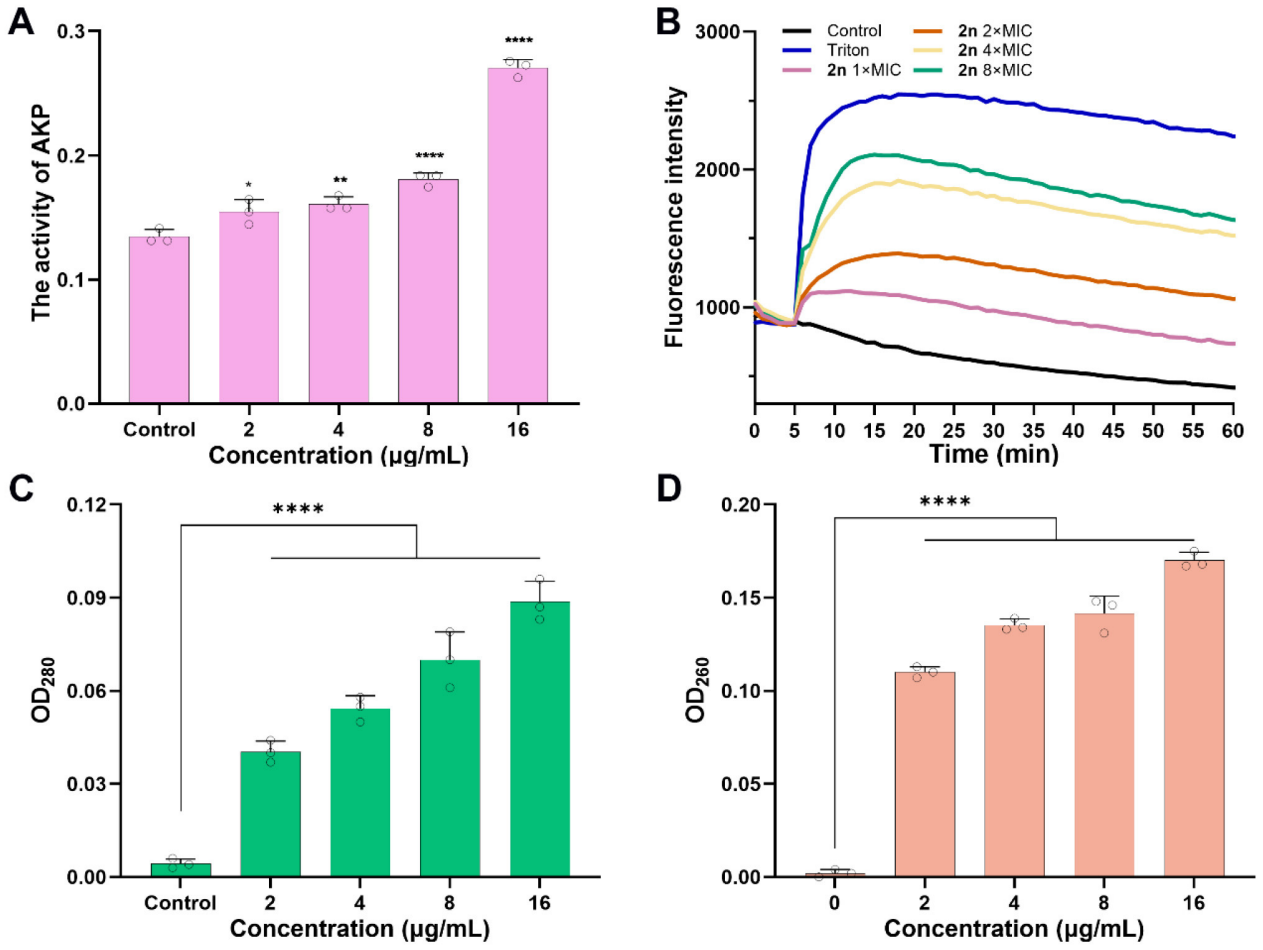
**(A)** AKP activity in bacterial supernatants after 4 h treatment with **2n** at 0, 2, 4, 8, and 16 μg/mL; **(B)** Membrane depolarization assay measured by DiSC35 fluorescence intensity; **(C)** Quantification of intracellular protein leakage; **(D)** Quantification of intracellular nucleic acid leakage.

AKP is a periplasmic enzyme whose extracellular release serves as a direct biomarker for bacterial cell envelopes integrity impairment (Tian et al., 2026). As shown in Figure 6A, compound **2n** induced a concentration-dependent increase in AKP activity in *S. aureus* supernatants after 4 h treatment. Compared to the untreated control, compound **2n** at 2 μg/mL (1 × MIC) caused a significant elevation in AKP release, with a 2-fold increase observed at 16 μg/mL (8 × MIC, ^****^P < 0.0001).

To comprehensively elucidate the bactericidal mechanism of compound **2n**, we systematically evaluated its effects on bacterial membrane integrity, potential, intracellular content retention, and cell viability. As shown in Figure 6B, the membrane depolarization assay using DiSC35 demonstrated that compound **2n** induced rapid, dose-dependent membrane potential disruption in *S. aureus*. The untreated control group maintained stable baseline fluorescence accompanied by a certain degree of fluorescence quenching, while the positive control Triton X-100 triggered maximal fluorescence enhancement. Treatment with compound **2n** at 1, 2, 4 and 8 × MIC elicited progressive, concentration-dependent increases in fluorescence intensity, confirming that compound **2n** effectively depolarizes the bacterial cytoplasmic membrane, a critical early event in membrane damage.

Consistent with the depolarization results, Figures 6C, D revealed concentration-dependent leakage of intracellular contents. After 4 h of compound **2n** treatment, the extracellular levels of proteins measured by OD_280_ values and nucleic acids measured by OD_260_ values increased dramatically in a dose-responsive manner. At the highest tested concentration (16 μg/mL, 8 × MIC), compound **2n** induced a 20-fold elevation in OD_280_ and an 85-fold increase in OD_260_ relative to the control group (^****^P < 0.0001), indicating irreversible membrane rupture.

**Figure 7:**
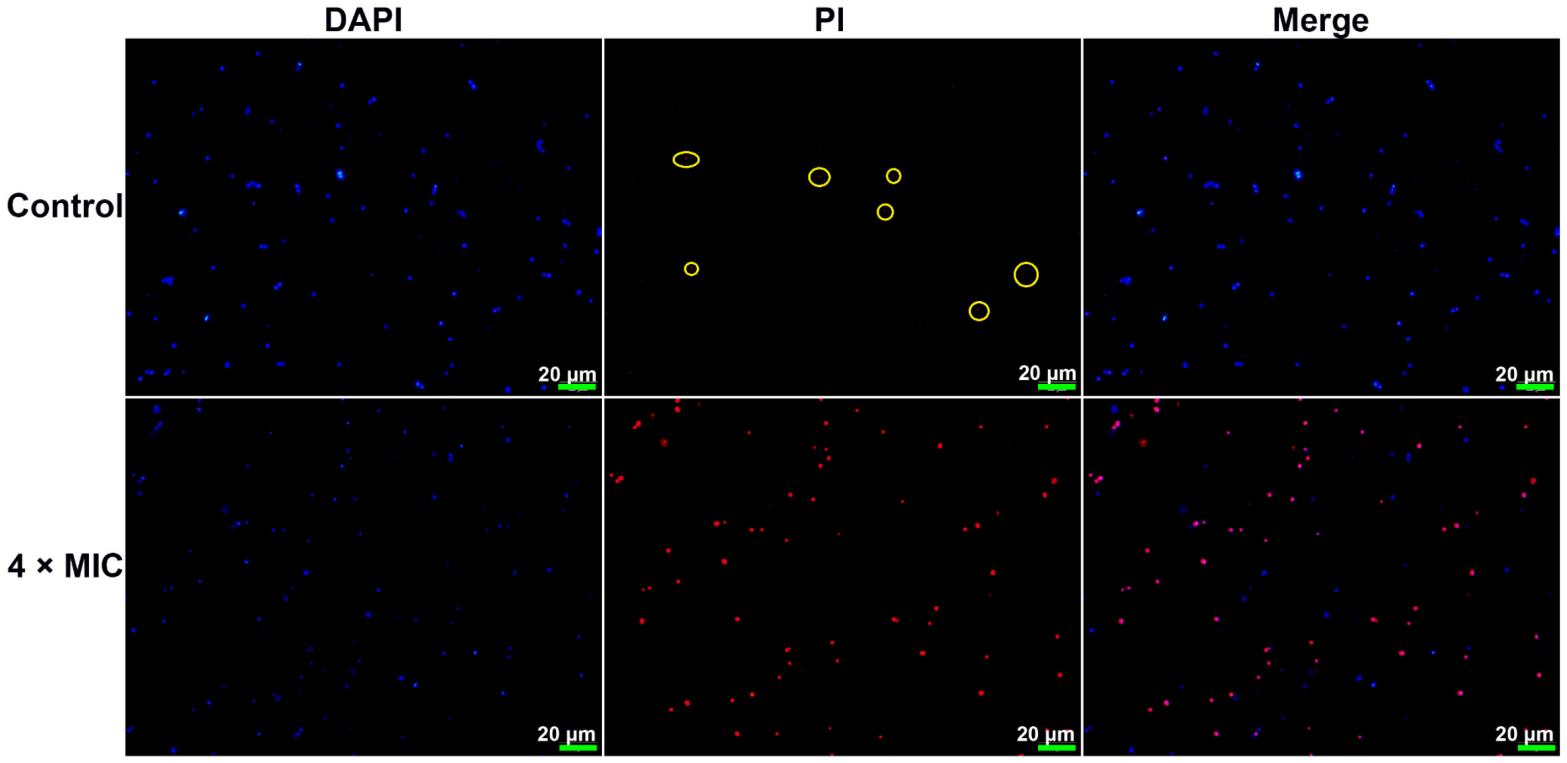
Assessment of *S. aureus* ATCC 29213 viability and membrane integrity following compound **2n** treatment by DAPI/PI double staining. Scale bar is 20 μm. Bacteria in the yellow circle show faint red fluorescence staining.

Subsequently, DAPI/PI double staining further validated the membrane-disrupting and bactericidal activity of compound **2n** (Figure 7). In the untreated control group, nearly all bacteria were stained blue by DAPI, a membrane-permeable nuclear stain, with negligible red PI fluorescence, and the weak sporadic PI fluorescence signal in the control group was derived from spontaneous physiological death of a small number of normal bacterial cells under routine culture conditions, confirming intact cell membranes and high viability. In contrast, treatment with **2n** at 4 × MIC resulted in a dramatic increase in PI-positive cells, demonstrating widespread membrane damage that allowed the membrane-impermeable dye PI to enter and label dead bacteria. The merged images showed extensive co-localization of blue and red fluorescence, confirming that compound **2n** effectively kills *S. aureus* by disrupting membrane integrity.

Collectively, these findings in phenomenological level confirm that compound **2n** exerts potent bactericidal activity via a comprehensive membrane-disrupting mechanism, which first depolarizes the bacterial membrane potential, then disrupts membrane integrity, leading to massive leakage of cytoplasmic proteins and nucleic acids, ultimately resulting in bacterial cell death. Notably, the observed AKP release and altered Gram staining might be secondary phenotypic manifestations resulting from membrane damage.

### 3.10 Interaction with DNA

**Figure 8:**
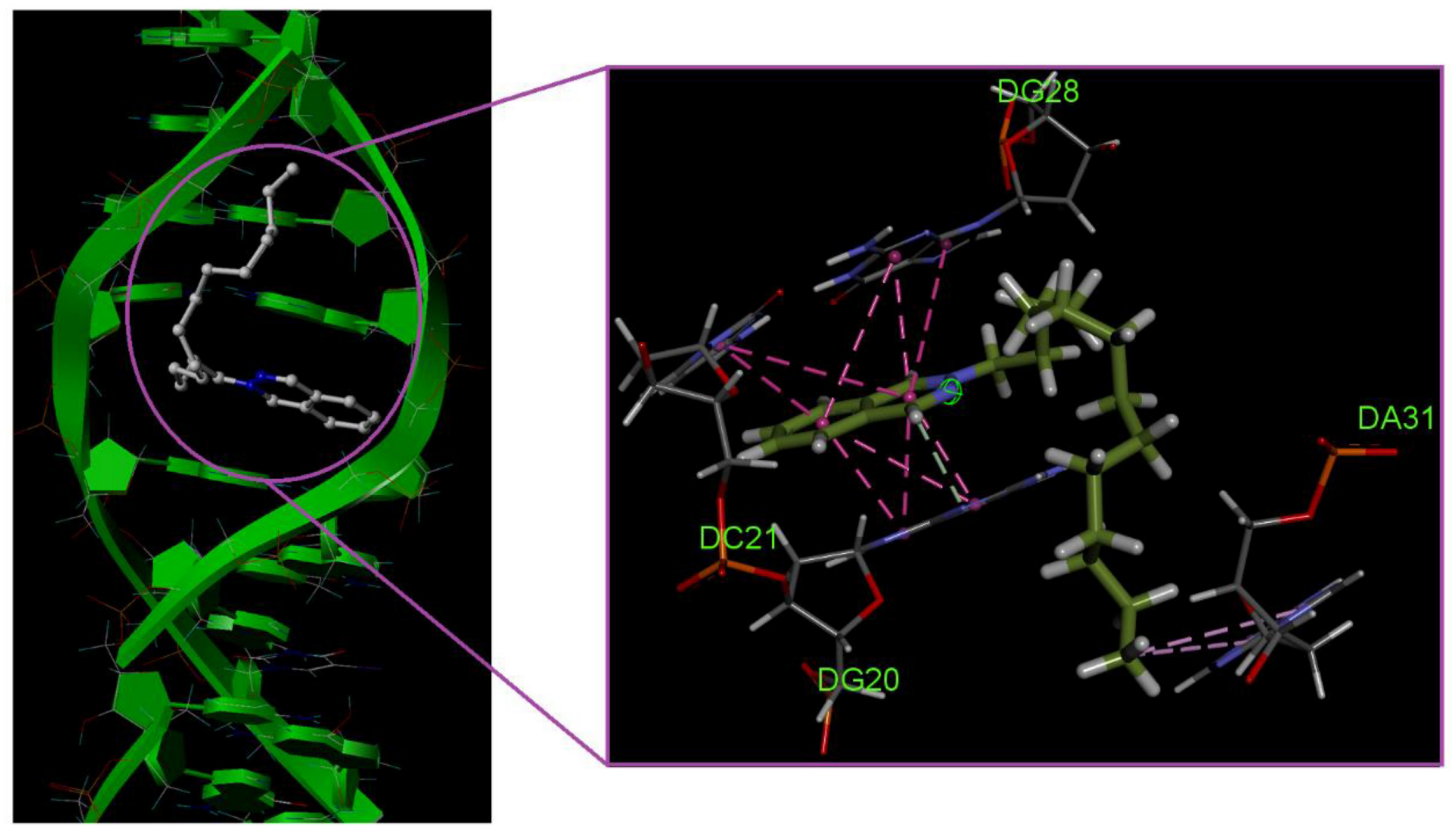
Molecular docking analysis between lead compound **2n** and DNA (PDB: 454D).

To elucidate the molecular mechanism underlying the DNA-binding activity of compound **2n**, we integrated molecular docking and fluorescence competitive displacement assays. As shown in Figure 8, molecular docking revealed that compound **2n** binds to the DNA double helix, with π-π stacking interactions as the dominant driving force (DG20, DC21, DG28). The phthalazine moiety, with its two fused aromatic rings and rigid planar skeleton, enables optimal geometric matching with DNA base pairs, maximizing stacking interactions and stabilizing the ligand-DNA complex. Furthermore, we also observed the hydrophobic interaction formed between the alkyl chain and the DNA residue (DA31). Notably, these docking results only reflect static binding inference rather than dynamic conformational changes (Alniss et al., 2024).

**Figure 9:**
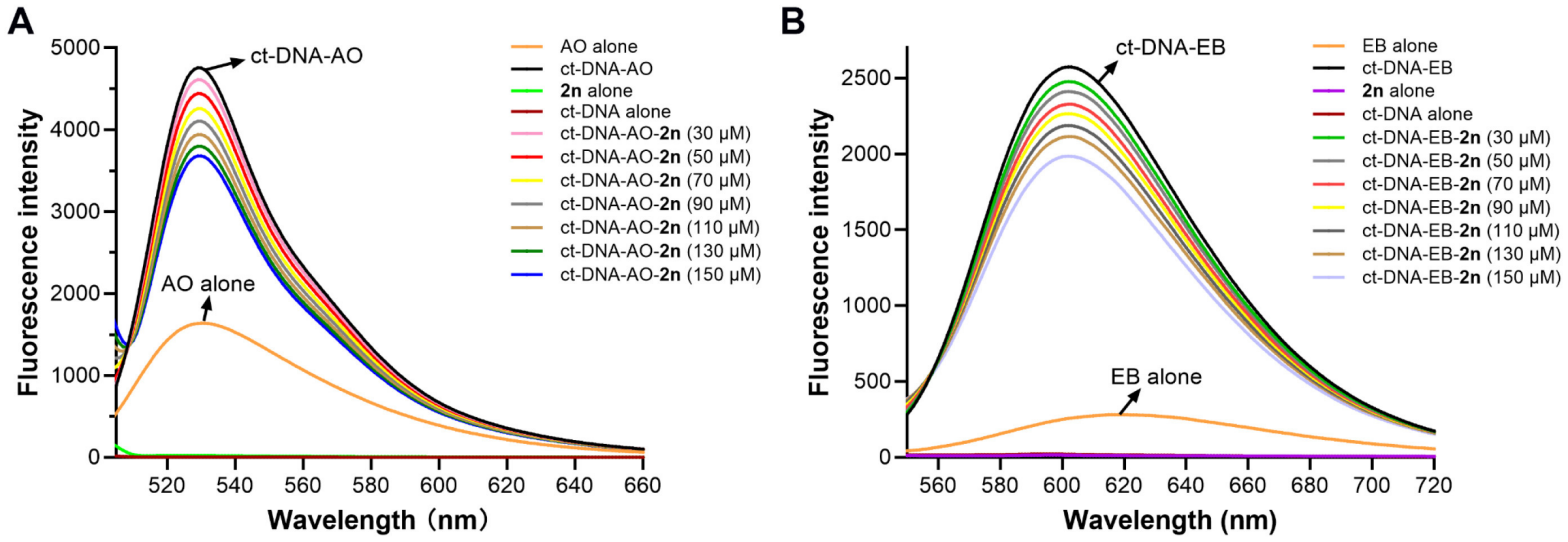
**(A)** Fluorescence emission spectra of the ct–DNA–AO complex in the absence and presence of increasing concentrations of compound **2n** (30–150 μM); **(B)** Fluorescence emission spectra of the ct–DNA–EB complex with increasing concentrations of **2n** (30–150 μM). AO alone, EB alone, **2n** alone, and ct-DNA alone are included as controls.

Consistently, fluorescence competitive displacement assays confirmed the DNA-binding affinity of compound **2n**. In the AO-displacement assay (Figure 9A), increasing concentrations of compound **2n** dose-dependently quenched the fluorescence of the ct-DNA-AO complex, indicating competitive displacement of the groove-binding ligand AO. Similarly, in the EB-displacement assay (Figure 9B), compound **2n** effectively reduced the fluorescence intensity of the ct-DNA-EB complex in a concentration-dependent manner, with significant quenching observed even at 30 μM. These *in vitro* results demonstrate that compound **2n** competes with both AO and EB for DNA binding sites, validating its potential DNA-binding capacity.

Notably, compound **2n** exhibited no intrinsic fluorescence under experimental conditions, eliminating interference and ensuring assay accuracy. Collectively, these data confirm that compound **2n** binds to DNA primarily via π-π stacking interactions enabled by its rigid phthalazine skeleton, which may disrupt DNA replication and transcription, thereby contributing to its antibacterial activity. It is worth emphasizing that the ct-DNA concentrations (30–150 μM) used in DNA binding assays were far higher than the MIC value (~ 4.9 μM). Accordingly, the detected DNA binding capability only reflects an *in vitro* molecular interaction. Such interaction presumably acts as a secondary effect following membrane damage, instead of serving as a dominant and independent intracellular bactericidal target. Systematic validations including intracellular accumulation assessment and transcriptional profiling are still required in future research to fully clarify the definite intracellular effects of the compound **2n**.

## 4 Conclusion

In this study, we successfully designed and synthesized a series of phthalazine-derived QACs and systematically evaluated their antibacterial properties, safety, stability, and mechanisms of action. The SAR analysis clearly indicated that the length of the *N*-alkyl substituent is a key factor regulating antibacterial activity, with compound **2n** bearing an C14 chain emerging as the lead compound. Compound **2n** exhibited potent broad-spectrum antibacterial activity against clinically relevant pathogens, including MRSA, with a MIC value of 2 μg/mL. It also showed favorable properties, including negligible hemolytic toxicity with HC_50_ greater than 64 μg/mL, minimal resistance evolution with only a 2-fold MIC increase after 20 passages, and excellent stability in physiological salts and against proteolytic enzymes, as well as moderate concentration-dependent biofilm disruption. Mechanistic investigations demonstrate that the core bactericidal mode of compound **2n** is cell membrane targeting. Collectively, this work provides a novel phthalazine-based scaffold for the development of QAC-based antibacterial agents and confirms that compound **2n** is a promising lead compound for further preclinical research. It offers a potential solution to the global challenge of drug-resistant bacterial infections.

## Data Availability

The original contributions presented in the study are included in the article/Supplementary material, further inquiries can be directed to the corresponding author/s.
