# Supplementary material for: Phthalazine-based quaternary ammonium salts: synthesis, biological evaluation and membrane-targeting mechanism against Staphylococcus aureus
Source: Front Microbiol. 2026 Jun 19;17:1864148. doi: 10.3389/fmicb.2026.1864148 (PMC13328279; doi:10.3389/fmicb.2026.1864148)
Supplement: Supplementary file 1 [file Table_1.docx]

Supplementary Material

# The characterization data of compounds 2a‒2v

*2-Methylphthalazin-2-ium iodide* ***2a***. Yellow solid; Yield 223 mg, 82%; Purity: 96.18%; ^1^H NMR (400 MHz, DMSO-*d_6_*) *δ*: 10.61 (s, 1H), 10.08 (s, 1H), 8.63 (d, *J* = 8.0 Hz, 1H), 8.60 (d, *J* = 8.0 Hz, 1H), 8.52 (t, *J* = 8.0 Hz, 1H), 8.43 (t, *J* = 8.0 Hz, 1H), 4.59 (s, 3H); ^13^C NMR (100 MHz, DMSO-*d_6_*) *δ*: 154.98, 152.18, 139.38, 136.74, 130.56, 128.78, 128.11, 127.64, 51.30. HRMS (ESI) m/z calcd for C_9_H_9_N_2_ [M‒I]^+^ 145.0766, found 145.0772.

*2-Ethylphthalazin-2-ium bromide* ***2b***. Brown solid; Yield 193 mg, 81%; Purity: 98.27%; ^1^H NMR (400 MHz, DMSO-*d_6_*) *δ*: 10.74 (s, 1H), 10.13 (s, 1H), 8.65 (d, *J* = 8.0 Hz, 1H), 8.62 (d, *J* = 8.0 Hz, 1H), 8.52 (t, *J* = 8.0 Hz, 1H), 8.43 (t, *J* = 8.0 Hz, 1H), 4.89 (q, *J* = 8.0 Hz, 2H), 1.68 (t, *J* = 8.0 Hz, 3H); ^13^C NMR (100 MHz, DMSO-*d_6_*) *δ*: 155.22, 151.48, 139.37, 136.64, 130.71, 128.77, 128.23, 127.93, 59.43, 14.94. HRMS (ESI) m/z calcd for C_10_H_11_N_2_ [M‒Br]^+^ 159.0922, found 159.0917.

*2-Propylphthalazin-2-ium bromide* ***2c***. Brown oil; Yield 202 mg, 80%; Purity: 95.77%; ^1^H NMR (400 MHz, DMSO-*d_6_*) *δ*: 10.75 (s, 1H), 10.13 (s, 1H), 8.65 (d, *J* = 8.0 Hz, 1H), 8.62 (d, *J* = 8.0 Hz, 1H), 8.53 (t, *J* = 8.0 Hz, 1H), 8.43 (t, *J* = 8.0 Hz, 1H), 4.79 (t, *J* = 8.0 Hz, 2H), 2.16‒2.07 (m, 2H), 0.98 (t, *J* = 8.0 Hz, 3H); ^13^C NMR (100 MHz, DMSO-*d_6_*) *δ*: 155.32, 151.79, 139.47, 136.67, 130.78, 128.80, 128.18, 127.98, 65.30, 23.04, 10.93. HRMS (ESI) m/z calcd for C_11_H_13_N_2_ [M‒Br]^+^ 173.1079, found 173.1084.

*2-Butylphthalazin-2-ium bromide* ***2d***. Brown oil; Yield 224 mg, 84%; Purity: 95.71%; ^1^H NMR (400 MHz, DMSO-*d_6_*) *δ*: 10.76 (s, 1H), 10.13 (s, 1H), 8.65 (d, *J* = 8.0 Hz, 1H), 8.62 (d, *J* = 8.0 Hz, 1H), 8.53 (t, *J* = 8.0 Hz, 1H), 8.43 (t, *J* = 8.0 Hz, 1H), 4.82 (t, *J* = 8.0 Hz, 2H), 2.11‒2.04 (m, 2H), 1.44‒1.38 (m, 2H), 0.96 (t, *J* = 8.0 Hz, 3H); ^13^C NMR (100 MHz, DMSO-*d_6_*) *δ*: 155.30, 151.77, 139.45, 136.66, 130.76, 128.79, 128.22, 127.96, 63.64, 31.52, 19.32, 13.88. HRMS (ESI) m/z calcd for C_12_H_15_N_2_ [M‒Br]^+^ 187.1235, found 187.1251.

*2-Pentylphthalazin-2-ium bromide* ***2e***. Brown oil; Yield 230 mg, 82%; Purity: 95.42%; ^1^H NMR (400 MHz, DMSO-*d_6_*) *δ*: 10.75 (s, 1H), 10.12 (s, 1H), 8.65 (d, *J* = 8.0 Hz, 1H), 8.62 (d, *J* = 8.0 Hz, 1H), 8.53 (t, *J* = 8.0 Hz, 1H), 8.43 (t, *J* = 8.0 Hz, 1H), 4.81 (t, *J* = 8.0 Hz, 2H), 2.11‒2.08 (m, 2H), 1.41‒1.32 (m, 4H), 0.89 (t, *J* = 8.0 Hz, 3H); ^13^C NMR (100 MHz, DMSO-*d_6_*) *δ*: 155.30, 151.76, 139.45, 136.67, 130.77, 128.79, 128.22, 127.96, 63.88, 29.23, 28.08, 22.09, 14.18. HRMS (ESI) m/z calcd for C_13_H_17_N_2_ [M‒Br]^+^ 201.1392, found 201.1422.

*2-Hexylphthalazin-2-ium bromide* ***2f***. Brown oil; Yield 242 mg, 82%; Purity: 95.13%; ^1^H NMR (400 MHz, DMSO-*d_6_*) *δ*: 10.74 (s, 1H), 10.12 (s, 1H), 8.64 (d, *J* = 8.0 Hz, 1H), 8.61 (d, *J* = 8.0 Hz, 1H), 8.53 (t, *J* = 8.0 Hz, 1H), 8.43 (t, *J* = 8.0 Hz, 1H), 4.81 (t, *J* = 8.0 Hz, 2H), 2.12‒2.05 (m, 2H), 1.41‒1.36 (m, 2H), 1.35‒1.24 (m, 4H), 0.87 (t, *J* = 8.0 Hz, 3H); ^13^C NMR (100 MHz, DMSO-*d_6_*) *δ*: 155.30, 151.75, 139.45, 136.67, 130.76, 128.79, 128.21, 127.95, 63.89, 31.13, 29.49, 25.62, 22.31, 14.31. HRMS (ESI) m/z calcd for C_14_H_19_N_2_ [M‒Br]^+^ 215.1542, found 215.1530.

*2-Heptylphthalazin-2-ium bromide* ***2g***. Brown oil; Yield 241 mg, 78%; Purity: 99.64%; ^1^H NMR (400 MHz, CDCl_3_) *δ*: 12.26 (s, 1H), 9.84 (s, 1H), 9.05 (d, *J* = 8.0 Hz, 1H), 8.55 (d, *J* = 8.0 Hz, 1H), 8.40 (t, *J* = 8.0 Hz, 1H), 8.28 (t, *J* = 8.0 Hz, 1H), 5.04 (t, *J* = 8.0 Hz, 2H), 2.20‒2.12 (m, 2H), 1.44‒1.38 (m, 2H), 1.36‒1.33 (m, 2H), 1.23 (s, 4H), 0.82 (t, *J* = 8.0 Hz, 3H); ^13^C NMR (100 MHz, CDCl_3_) *δ*: 154.23, 151.66, 139.35, 136.35, 131.74, 128.16, 127.97, 127.67, 64.33, 31.48, 30.33, 28.71, 26.13, 22.50, 14.05. HRMS (ESI) m/z calcd for C_15_H_21_N_2_ [M‒Br]^+^ 229.1705, found 229.1727.

*2-Octylphthalazin-2-ium bromide* ***2h***. Brown oil; Yield 268 mg, 83%; Purity: 95.45%; ^1^H NMR (400 MHz, CDCl_3_) *δ*: 12.15 (s, 1H), 9.76 (s, 1H), 9.09 (d, *J* = 12.0 Hz, 1H), 8.48 (d, *J* = 8.0 Hz, 1H), 8.39 (t, *J* = 8.0 Hz, 1H), 8.29 (t, *J* = 8.0 Hz, 1H), 5.05 (t, *J* = 8.0 Hz, 2H), 2.20‒2.13 (m, 2H), 1.45‒1.39 (m, 2H), 1.37‒1.31 (m, 2H), 1.28‒1.18 (m, 6H), 0.83 (t, *J* = 8.0 Hz, 3H); ^13^C NMR (100 MHz, CDCl_3_) *δ*: 154.08, 151.69, 139.32, 136.39, 131.91, 128.21, 127.75, 127.63, 64.41, 50.67, 31.67, 30.34, 29.00, 26.19, 22.59, 14.09. HRMS (ESI) m/z calcd for C_16_H_23_N_2_ [M‒Br]^+^ 243.1861, found 243.1853.

*2-Nonylphthalazin-2-ium bromide* ***2i***. Brown oil; Yield 293 mg, 87%; Purity: 97.64%; ^1^H NMR (400 MHz, CDCl_3_) *δ*: 12.35 (s, 1H), 9.69 (s, 1H), 9.13 (d, *J* = 8.0 Hz, 1H), 8.41‒8.37 (m, 2H), 8.32‒8.29 (m, 1H), 5.07 (t, *J* = 8.0 Hz, 2H), 2.22‒2.14 (m, 2H), 1.46‒1.41 (m, 2H), 1.37‒1.33 (m, 2H), 1.27‒1.23 (m, 8H), 0.84 (t, *J* = 8.0 Hz, 3H); ^13^C NMR (100 MHz, CDCl_3_) *δ*: 153.89, 151.89, 139.28, 136.39, 132.11, 128.27, 127.58, 127.52, 64.42, 31.79, 30.38, 29.30, 29.17, 29.07, 26.20, 22.64, 14.13. HRMS (ESI) m/z calcd for C_17_H_25_N_2_ [M‒Br]^+^ 257.2012, found 257.2005.

*2-Decylphthalazin-2-ium bromide* ***2j***. Brown oil; Yield 298 mg, 85%; Purity: 96.45%; ^1^H NMR (400 MHz, CDCl_3_) *δ*: 12.23 (s, 1H), 9.81 (s, 1H), 9.07 (d, *J* = 8.0 Hz, 1H), 8.52 (d, *J* = 8.0 Hz, 1H), 8.39 (t, *J* = 8.0 Hz, 1H), 8.29 (t, *J* = 8.0 Hz, 1H), 5.05 (t, *J* = 8.0 Hz, 2H), 2.20‒2.12 (m, 2H), 1.44‒1.38 (m, 2H), 1.35‒1.29 (m, 2H), 1.26‒1.20 (m, 10H), 0.83 (t, *J* = 8.0 Hz, 3H); ^13^C NMR (100 MHz, CDCl_3_) *δ*: 154.16, 151.68, 139.33, 136.36, 131.81, 128.18, 127.88, 127.65, 64.37, 31.83, 30.35, 29.45, 29.33, 29.24, 29.06, 26.19, 22.66, 14.14. HRMS (ESI) m/z calcd for C_18_H_27_N_2_ [M‒Br]^+^ 271.2174, found 271.2200.

*2-Undecylphthalazin-2-ium bromide* ***2k***. Brown oil; Yield 314 mg, 86%; Purity: 99.27%; ^1^H NMR (400 MHz, CDCl_3_) *δ*: 12.25 (s, 1H), 9.77 (s, 1H), 9.08 (d, *J* = 8.0 Hz, 1H), 8.49 (d, *J* = 8.0 Hz, 1H), 8.41 (t, *J* = 8.0 Hz, 1H), 8.29 (t, *J* = 8.0 Hz, 1H), 5.05 (t, *J* = 8.0 Hz, 2H), 2.20‒2.13 (m, 2H), 1.46‒1.39 (m, 2H), 1.35‒1.31 (m, 2H), 1.25‒1.21 (m, 12H), 0.84 (t, *J* = 8.0 Hz, 3H); ^13^C NMR (100 MHz, CDCl_3_) *δ*: 154.08, 151.74, 139.31, 136.37, 131.91, 128.21, 127.77, 127.63, 64.39, 31.88, 30.36, 29.55, 29.51, 29.34, 29.30, 29.07, 26.20, 22.69, 14.15. HRMS (ESI) m/z calcd for C_19_H_29_N_2_ [M‒Br]^+^ 285.2331, found 285.2354.

*2-Dodecylphthalazin-2-ium bromide* ***2l***. Brown oil; Yield 322 mg, 85%; Purity: 98.98%; ^1^H NMR (400 MHz, CDCl_3_) *δ*: 12.23 (s, 1H), 9.76 (s, 1H), 9.09 (d, *J* = 8.0 Hz, 1H), 8.47 (d, *J* = 8.0 Hz, 1H), 8.39 (t, *J* = 8.0 Hz, 1H), 8.29 (t, *J* = 8.0 Hz, 1H), 5.05 (t, *J* = 8.0 Hz, 2H), 2.20‒2.13 (m, 2H), 1.43‒1.39 (m, 2H), 1.35‒1.32 (m, 2H), 1.27‒1.21 (m, 14H), 0.84 (t, *J* = 8.0 Hz, 3H); ^13^C NMR (100 MHz, CDCl_3_) *δ*: 154.04, 151.72, 139.29, 136.36, 131.92, 128.20, 127.72, 127.61, 64.40, 31.90, 30.36, 29.59, 29.51, 29.34, 29.08, 26.20, 22.69, 14.16. HRMS (ESI) m/z calcd for C_20_H_31_N_2_ [M‒Br]^+^ 299.2487, found 299.2488.

*2-Tridecylphthalazin-2-ium bromide* ***2m***. Brown oil; Yield 327 mg, 83%; Purity: 99.04%; ^1^H NMR (400 MHz, CDCl_3_) *δ*: 12.29 (s, 1H), 9.72 (s, 1H), 9.11 (d, *J* = 8.0 Hz, 1H), 8.44 (d, *J* = 8.0 Hz, 1H), 8.39 (t, *J* = 8.0 Hz, 1H), 8.30 (t, *J* = 8.0 Hz, 1H), 5.06 (t, *J* = 8.0 Hz, 2H), 2.22‒2.14 (m, 2H), 1.88 (s, 2H), 1.46‒1.41 (m, 2H), 1.39‒1.33 (m, 2H), 1.27‒1.22 (m, 14H), 0.85 (t, *J* = 8.0 Hz, 3H); ^13^C NMR (100 MHz, CDCl_3_) *δ*: 153.94, 151.81, 139.25, 136.35, 132.03, 128.26, 127.61, 64.42, 31.90, 30.34, 29.62, 29.58, 29.49, 29.33, 29.06, 26.20, 22.68, 14.12. HRMS (ESI) m/z calcd for C_21_H_33_N_2_ [M‒Br]^+^ 313.2644, found 313.2660.

*2-Tetradecylphthalazin-2-ium bromide* ***2n***. Brown oil; Yield 346 mg, 86%; Purity: 99.86%; ^1^H NMR (400 MHz, CDCl_3_) *δ*: 12.28 (s, 1H), 9.74 (s, 1H), 9.10 (d, *J* = 8.0 Hz, 1H), 8.46 (d, *J* = 8.0 Hz, 1H), 8.39 (t, *J* = 8.0 Hz, 1H), 8.30 (t, *J* = 8.0 Hz, 1H), 5.06 (t, *J* = 8.0 Hz, 2H), 2.21‒2.14 (m, 2H), 1.92 (s, 2H), 1.45‒1.40 (m, 2H), 1.36‒1.33 (m, 2H), 1.27‒1.22 (m, 16H), 0.85 (t, *J* = 8.0 Hz, 3H); ^13^C NMR (100 MHz, CDCl_3_) *δ*: 153.98, 151.78, 139.26, 136.35, 131.97, 128.24, 127.67, 127.63, 64.41, 31.91, 30.34, 29.67, 29.63, 29.59, 29.50, 29.34, 29.06, 26.20, 22.68, 14.12. HRMS (ESI) m/z calcd for C_22_H_35_N_2_ [M‒Br]^+^ 327.2800, found 327.2801.

*2-(7-Bromoheptyl)phthalazin-2-ium bromide* ***2o***. Brown oil; Yield 326 mg, 85%; Purity: 99.30%; ^1^H NMR (400 MHz, CDCl_3_) *δ*: 12.27 (s, 1H), 9.75 (s, 1H), 9.08 (d, *J* = 8.0 Hz, 1H), 8.47 (d, *J* = 8.0 Hz, 1H), 8.40 (t, *J* = 8.0 Hz, 1H), 8.31 (t, *J* = 8.0 Hz, 1H), 5.08 (t, *J* = 8.0 Hz, 2H), 3.38 (t, *J* = 8.0 Hz, 2H), 2.33 (s, 2H), 2.20 (t, *J* = 8.0 Hz, 2H), 1.82 (t, *J* = 8.0 Hz, 2H), 1.42 (s, 2H), 1.23 (s, 2H); ^13^C NMR (100 MHz, CDCl_3_) *δ*: 154.07, 151.82, 139.35, 136.43, 131.94, 128.23, 127.72, 127.66, 64.21, 33.95, 32.48, 30.10, 28.10, 27.77, 25.96. HRMS (ESI) m/z calcd for C_15_H_20_BrN_2_ [M‒Br]^+^ 307.0804, found 307.0772.

*2-(8-Bromooctyl)phthalazin-2-ium bromide* ***2p***. Brown oil; Yield 318 mg, 79%; Purity: 99.28%; ^1^H NMR (400 MHz, CDCl_3_) *δ*: 12.25 (s, 1H), 9.75 (s, 1H), 9.08 (d, *J* = 8.0 Hz, 1H), 8.47 (d, *J* = 8.0 Hz, 1H), 8.40 (t, *J* = 8.0 Hz, 1H), 8.31 (t, *J* = 8.0 Hz, 1H), 5.07 (t, *J* = 8.0 Hz, 2H), 3.37 (t, *J* = 8.0 Hz, 2H), 2.50 (s, 2H), 2.23‒2.15 (m, 2H), 1.84‒1.77 (m, 2H), 1.44‒1.37 (m, 4H), 1.31 (t, *J* = 8.0 Hz, 2H); ^13^C NMR (100 MHz, CDCl_3_) *δ*: 154.06, 151.76, 139.33, 136.41, 131.92, 128.22, 127.72, 127.65, 64.29, 34.05, 32.61, 30.18, 28.78, 28.37, 27.91, 26.01. HRMS (ESI) m/z calcd for C_16_H_22_BrN_2_ [M‒Br]^+^ 321.0961, found 321.0945.

*2-(9-Bromononyl)phthalazin-2-ium bromide* ***2q***. Brown oil; Yield 337 mg, 81%; Purity: 97.83%; ^1^H NMR (400 MHz, CDCl_3_) *δ*: 12.29 (s, 1H), 9.73 (s, 1H), 9.10 (d, *J* = 8.0 Hz, 1H), 8.45 (d, *J* = 8.0 Hz, 1H), 8.40 (t, *J* = 8.0 Hz, 1H), 8.31 (t, *J* = 8.0 Hz, 1H), 5.07 (t, *J* = 8.0 Hz, 2H), 3.37 (t, *J* = 8.0 Hz, 2H), 2.21‒2.27 (m, 2H), 2.14‒2.13 (m, 2H), 1.85‒1.77 (m, 2H), 1.46‒1.41 (m, 2H), 1.40‒1.35 (m, 2H), 1.29‒1.27 (m, 4H); ^13^C NMR (100 MHz, CDCl_3_) *δ*: 153.98, 151.80, 139.30, 136.40, 131.99, 128.24, 127.63, 64.34, 34.10, 32.69, 30.25, 29.07, 28.88, 28.53, 28.02, 26.10. HRMS (ESI) m/z calcd for C_17_H_24_BrN_2_ [M‒Br]^+^ 335.1123, found 335.1129.

*2-(10-Bromodecyl)phthalazin-2-ium bromide* ***2r***. Brown oil; Yield 348 mg, 81%; Purity: 95.09%; ^1^H NMR (400 MHz, CDCl_3_) *δ*: 12.35 (s, 1H), 9.74 (s, 1H), 9.10 (d, *J* = 8.0 Hz, 1H), 8.46 (d, *J* = 8.0 Hz, 1H), 8.40 (t, *J* = 8.0 Hz, 1H), 8.30 (t, *J* = 8.0 Hz, 1H), 5.07 (t, *J* = 8.0 Hz, 2H), 3.38 (t, *J* = 8.0 Hz, 2H), 2.22‒2.15 (m, 2H), 1.85‒1.78 (m, 2H), 1.45‒1.34 (m, 6H), 1.28‒1.26 (m, 6H); ^13^C NMR (100 MHz, CDCl_3_) *δ*: 153.99, 151.82, 139.29, 136.37, 131.97, 128.24, 127.67, 127.63, 64.34, 34.13, 32.76, 30.29, 29.22, 29.15, 28.96, 28.63, 28.07, 26.14. HRMS (ESI) m/z calcd for C_18_H_26_BrN_2_ [M‒Br]^+^ 349.1279, found 349.1290.

*2-(11-Bromoundecyl)phthalazin-2-ium bromide* ***2s***. Brown oil; Yield 333 mg, 75%; Purity: 97.65%; ^1^H NMR (400 MHz, CDCl_3_) *δ*: 12.33 (s, 1H), 9.73 (s, 1H), 9.10 (d, *J* = 8.0 Hz, 1H), 8.46 (d, *J* = 8.0 Hz, 1H), 8.39 (t, *J* = 8.0 Hz, 1H), 8.30 (t, *J* = 8.0 Hz, 1H), 5.07 (t, *J* = 8.0 Hz, 2H), 3.38 (t, *J* = 8.0 Hz, 2H), 2.22‒2.14 (m, 2H), 1.85‒1.78 (m, 2H), 1.47‒1.33 (m, 6H), 1.27‒1.26 (m, 8H); ^13^C NMR (100 MHz, CDCl_3_) *δ*: 153.98, 151.81, 139.28, 136.36, 131.99, 128.25, 127.65, 127.62, 64.37, 34.14, 32.79, 30.32, 29.31, 29.24, 29.00, 28.67, 28.11, 26.17. HRMS (ESI) m/z calcd for C_19_H_28_BrN_2_ [M‒Br]^+^ 363.1430, found 363.1409.

*2-(12-Bromododecyl)phthalazin-2-ium bromide* ***2t***. Brown oil; Yield 375 mg, 82%; Purity: 98.47%; ^1^H NMR (400 MHz, CDCl_3_) *δ*: 12.37 (s, 1H), 9.72 (s, 1H), 9.11 (d, *J* = 8.0 Hz, 1H), 8.45 (d, *J* = 8.0 Hz, 1H), 8.39 (t, *J* = 8.0 Hz, 1H), 8.30 (t, *J* = 8.0 Hz, 1H), 5.07 (t, *J* = 8.0 Hz, 2H), 3.38 (t, *J* = 8.0 Hz, 2H), 2.22‒2.15 (m, 2H), 1.86‒1.79 (m, 2H), 1.45‒1.35 (m, 6H), 1.28‒1.24 (m, 10H); ^13^C NMR (100 MHz, CDCl_3_) *δ*: 153.93, 151.84, 139.27, 136.36, 132.02, 128.26, 127.61, 64.38, 34.15, 32.81, 30.33, 29.40, 29.35, 29.28, 29.02, 28.71, 28.14, 26.18. HRMS (ESI) m/z calcd for C_20_H_30_BrN_2_ [M‒Br]^+^ 377.1587, found 377.1573.

*2-(13-Bromotridecyl)phthalazin-2-ium* ***2u***. Brown oil; Yield 363 mg, 77%; Purity: 98.73%; ^1^H NMR (400 MHz, CDCl_3_) *δ*: 12.34 (s, 1H), 9.73 (s, 1H), 9.10 (d, *J* = 8.0 Hz, 1H), 8.46 (d, *J* = 8.0 Hz, 1H), 8.39 (t, *J* = 8.0 Hz, 1H), 8.30 (t, *J* = 8.0 Hz, 1H), 5.06 (t, *J* = 8.0 Hz, 2H), 3.38 (t, *J* = 8.0 Hz, 2H), 2.22‒2.14 (m, 2H), 1.87‒1.79 (m, 2H), 1.45‒1.33 (m, 6H), 1.28‒1.23 (m, 12H); ^13^C NMR (100 MHz, CDCl_3_) *δ*: 153.97, 151.82, 139.27, 136.35, 131.99, 128.25, 127.65, 127.63, 64.38, 34.15, 32.82, 30.34, 29.49, 29.44, 29.39, 29.30, 29.04, 28.73, 28.15, 26.19. HRMS (ESI) m/z calcd for C_21_H_32_BrN_2_ [M‒Br]^+^ 391.1749, found 391.1799.

*2-(14-Bromotetradecyl)phthalazin-2-ium* ***2v***. Brown oil; Yield 364 mg, 75%; Purity: 99.24%; ^1^H NMR (400 MHz, CDCl_3_) *δ*: 12.37 (s, 1H), 9.71 (s, 1H), 9.11 (d, *J* = 8.0 Hz, 1H), 8.44‒8.37 (m, 2H), 8.30 (t, *J* = 8.0 Hz, 1H), 5.06 (t, *J* = 8.0 Hz, 2H), 3.38 (t, *J* = 8.0 Hz, 2H), 2.18 (t, *J* = 8.0 Hz, 2H), 1.83 (t, *J* = 8.0 Hz, 2H), 1.45‒1.33 (m, 6H), 1.28‒1.22 (m, 14H); ^13^C NMR (100 MHz, CDCl_3_) *δ*: 153.92, 151.85, 139.25, 136.35, 132.04, 128.27, 127.61, 127.58, 64.41, 34.14, 32.83, 30.34, 29.60, 29.53, 29.49, 29.46, 29.41, 29.31, 29.05, 28.74, 28.16, 26.20. HRMS (ESI) m/z calcd for C_22_H_34_BrN_2_ [M‒Br]^+^ 405.1906, found 405.1949.

# The NMR, HRMS and LC data of compounds 2a–2v


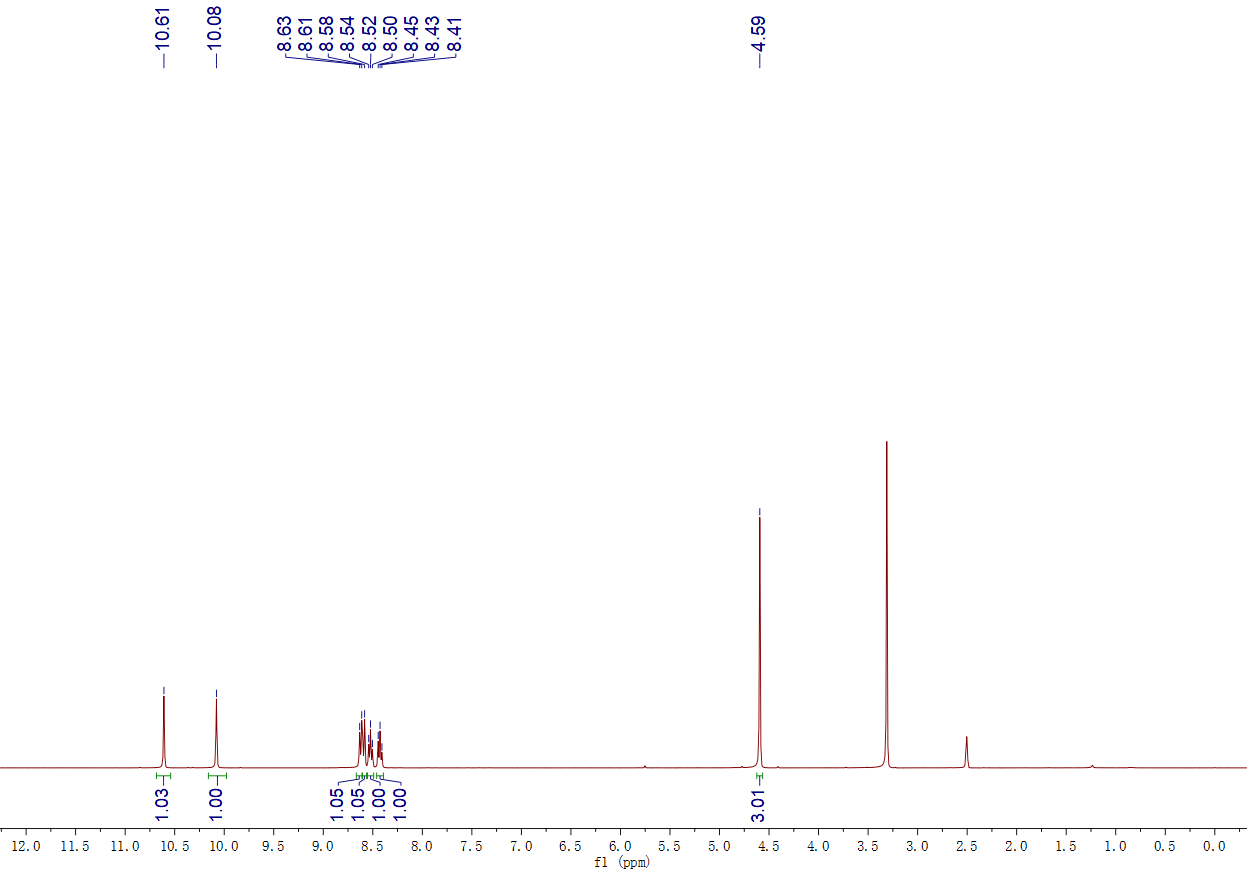


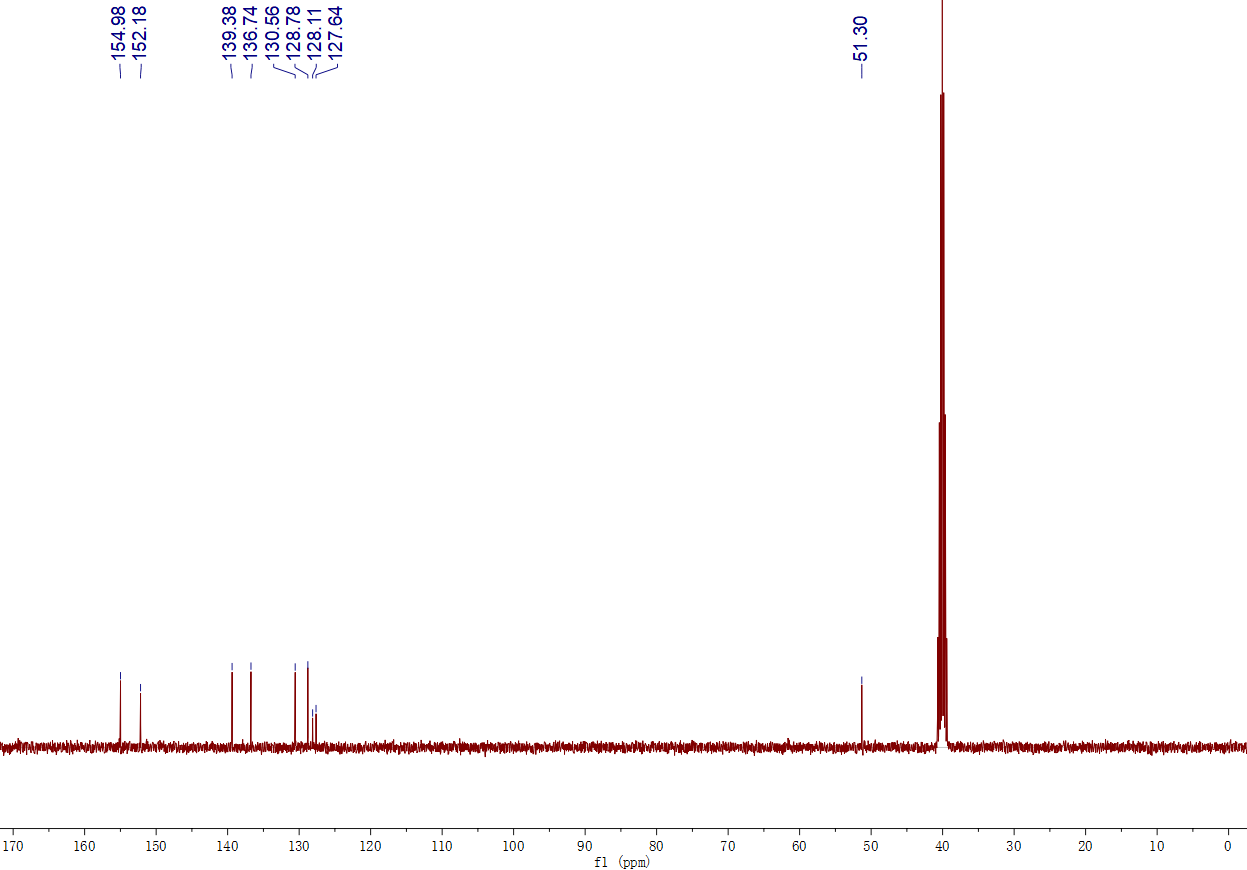

**Figure S1**. The NMR, HRMS and LC data of compound **2a**.


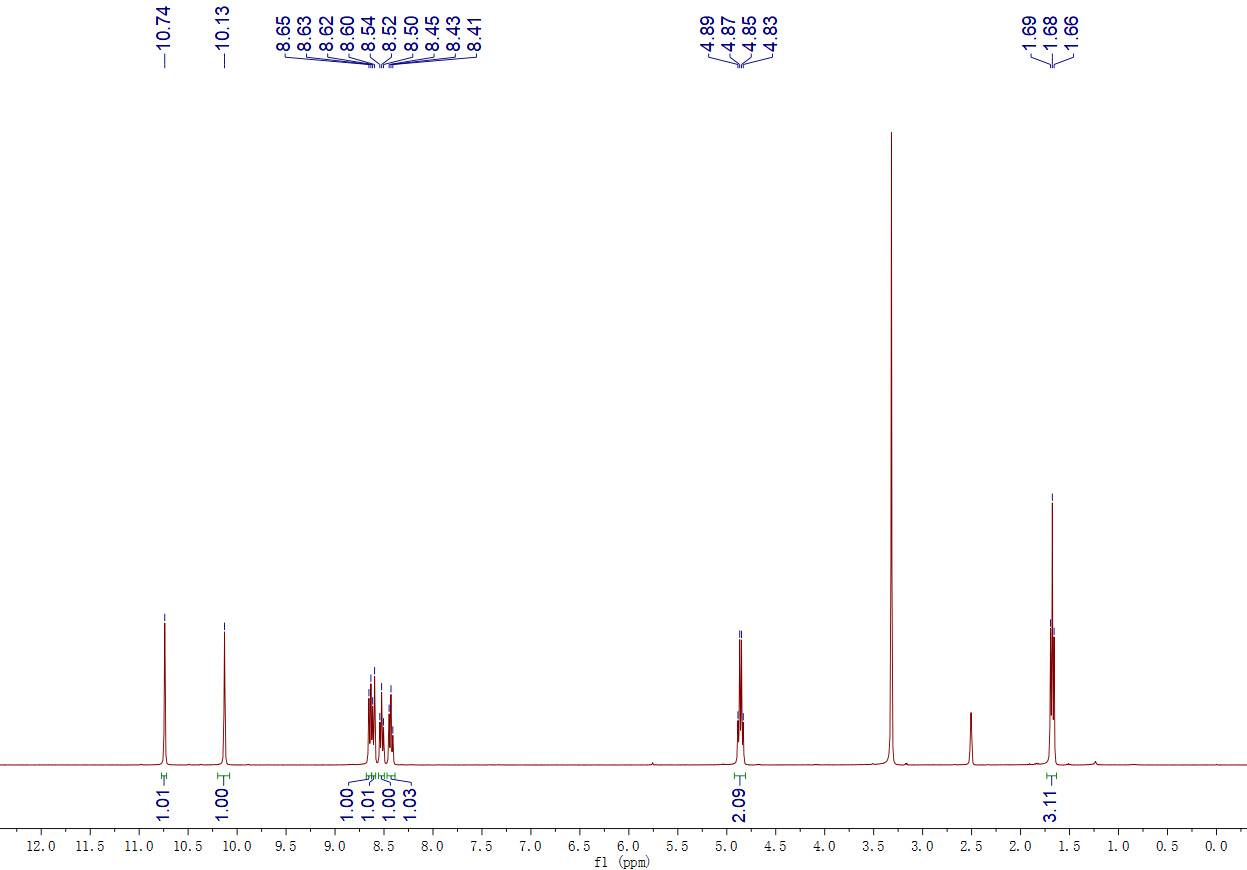


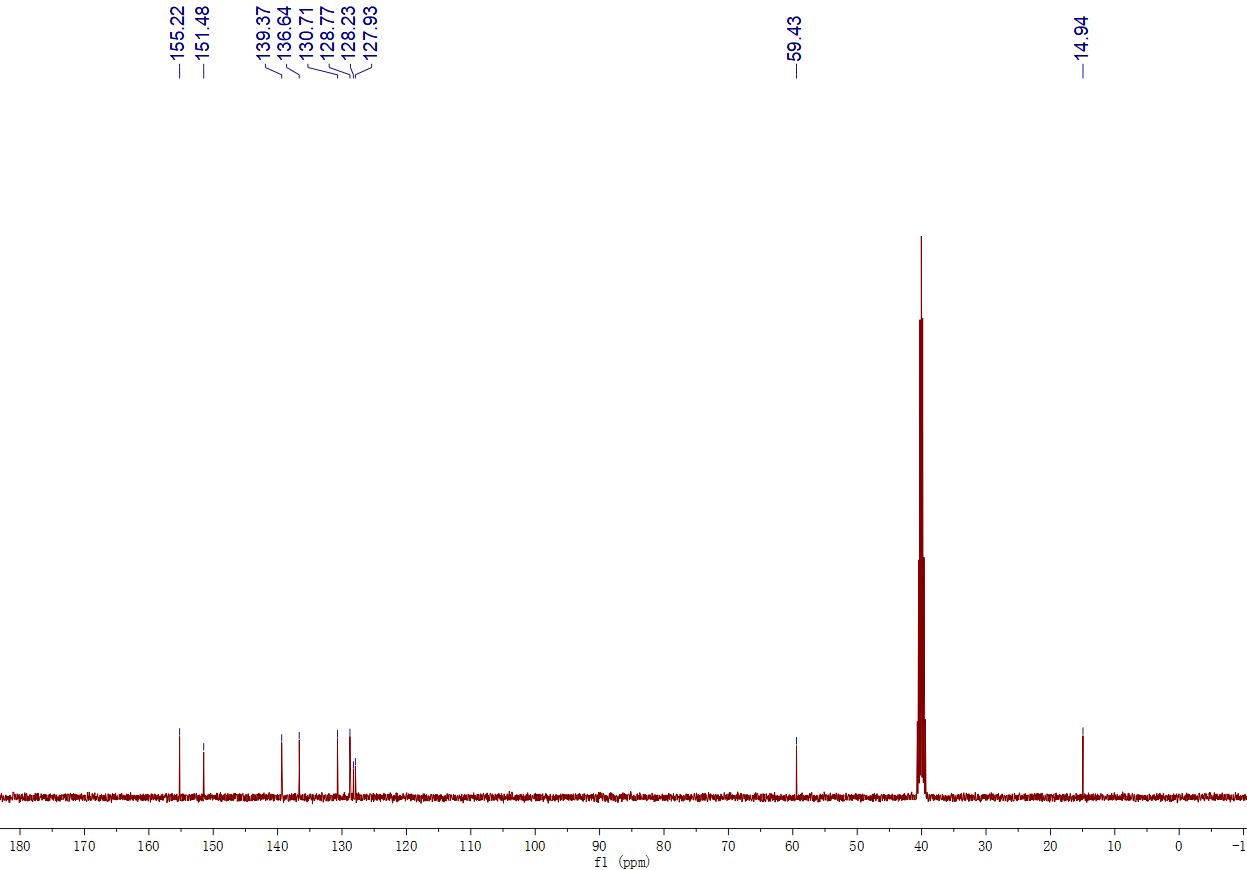

**Figure S2**. The NMR, HRMS and LC data of compound **2b**.


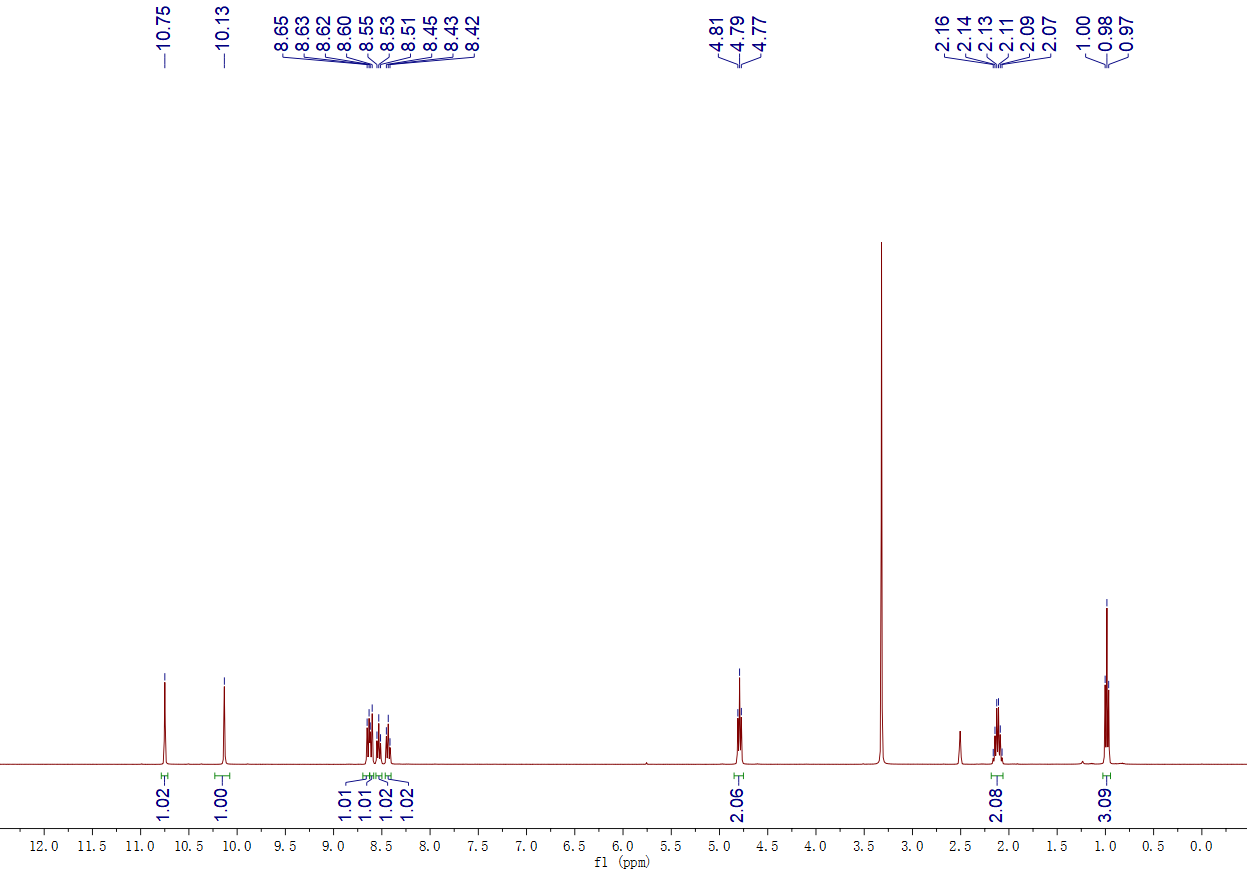


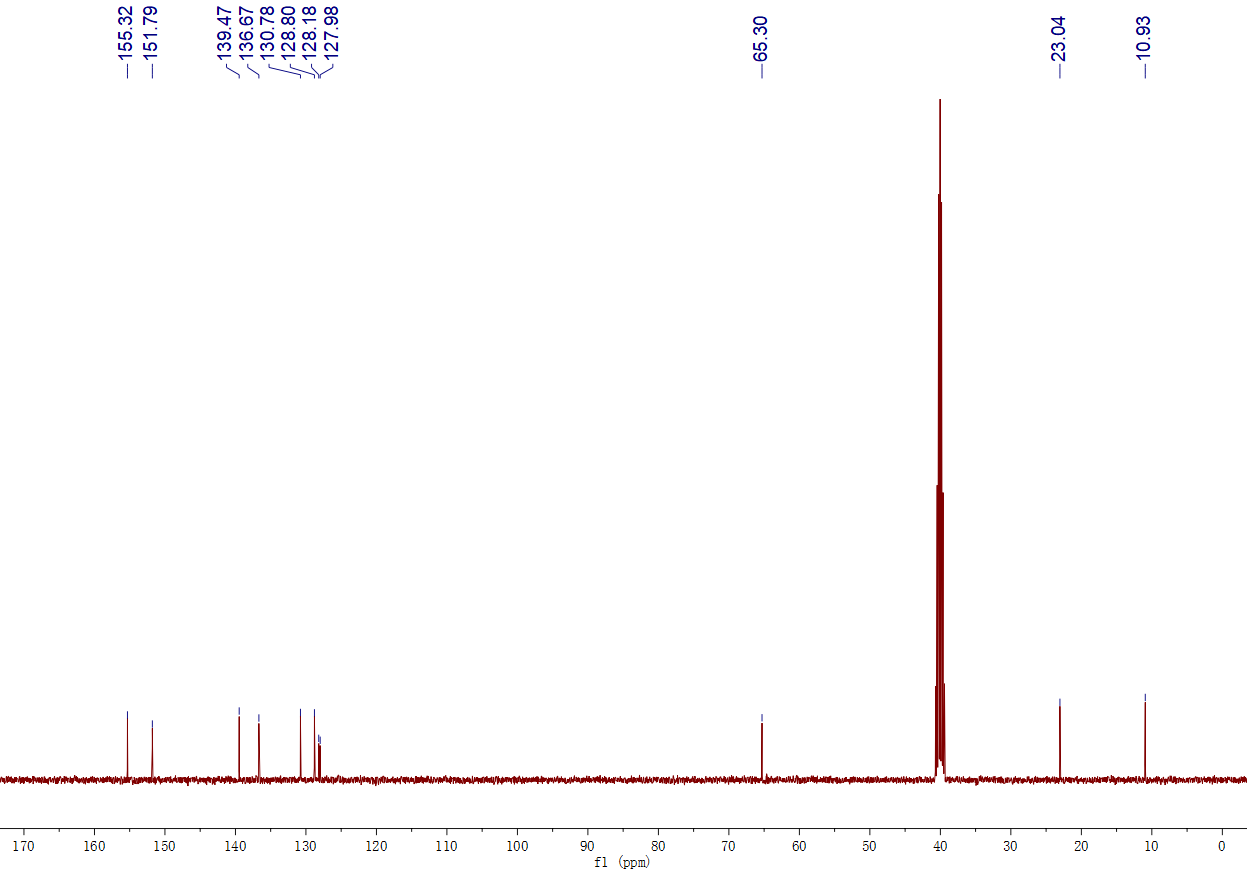

**Figure S3**. The NMR, HRMS and LC data of compound **2c**.


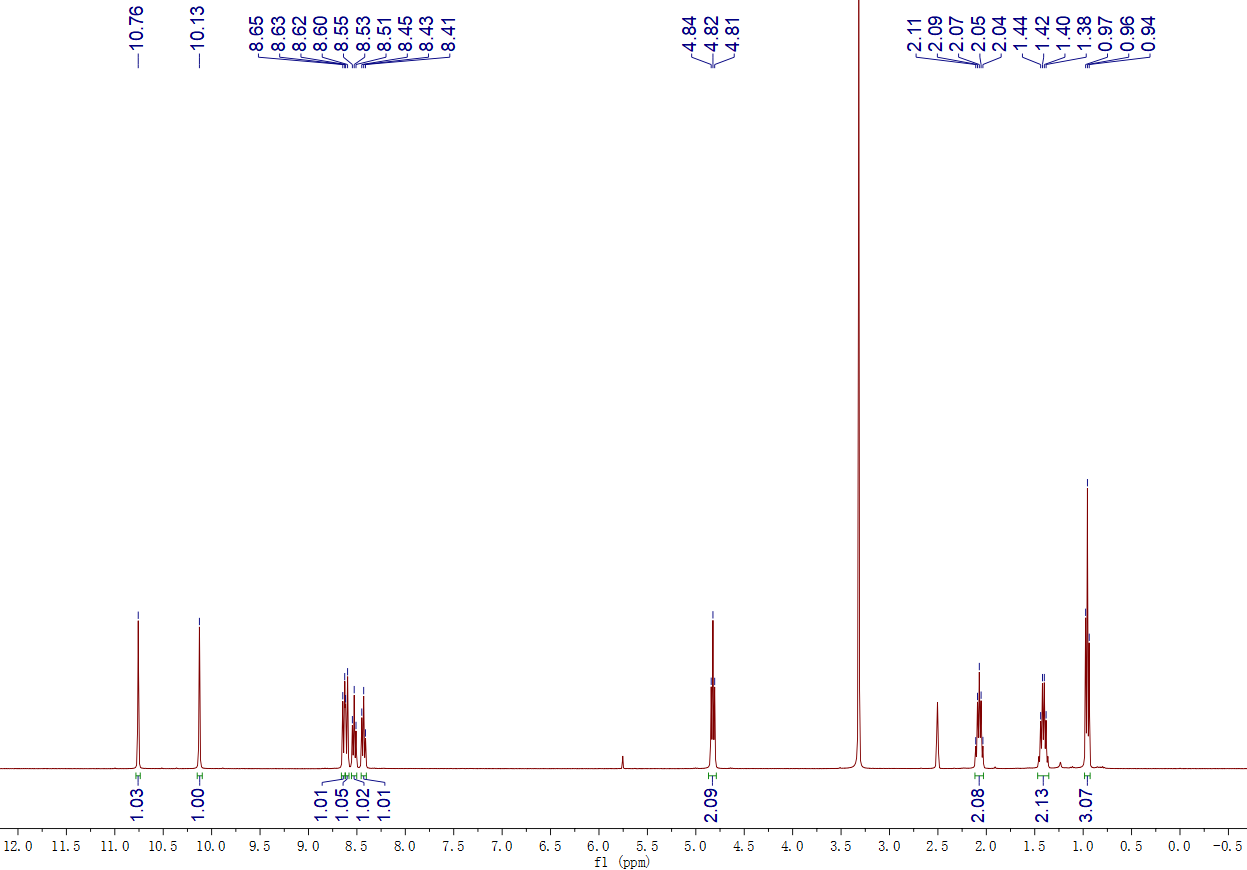


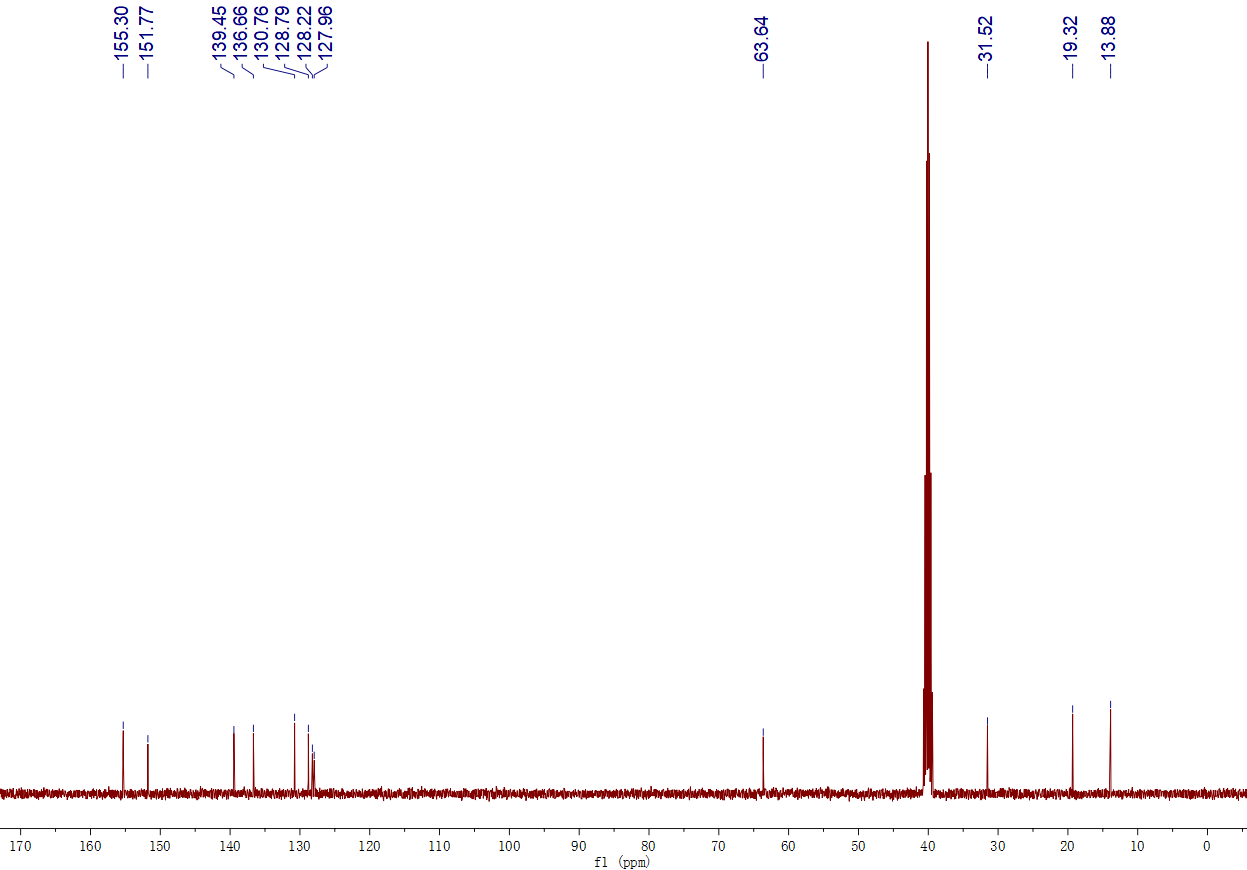

**Figure S4**. The NMR, HRMS and LC data of compound **2d**.


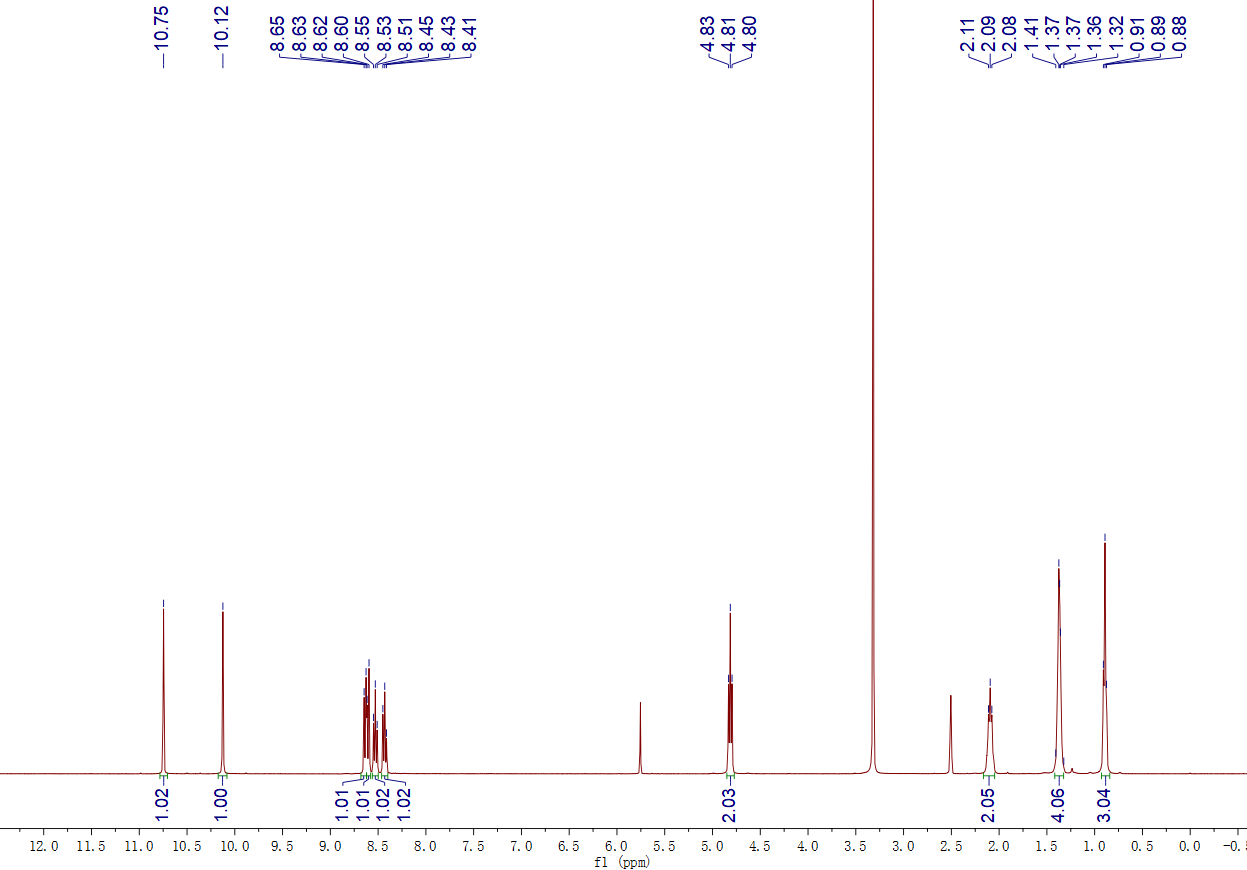


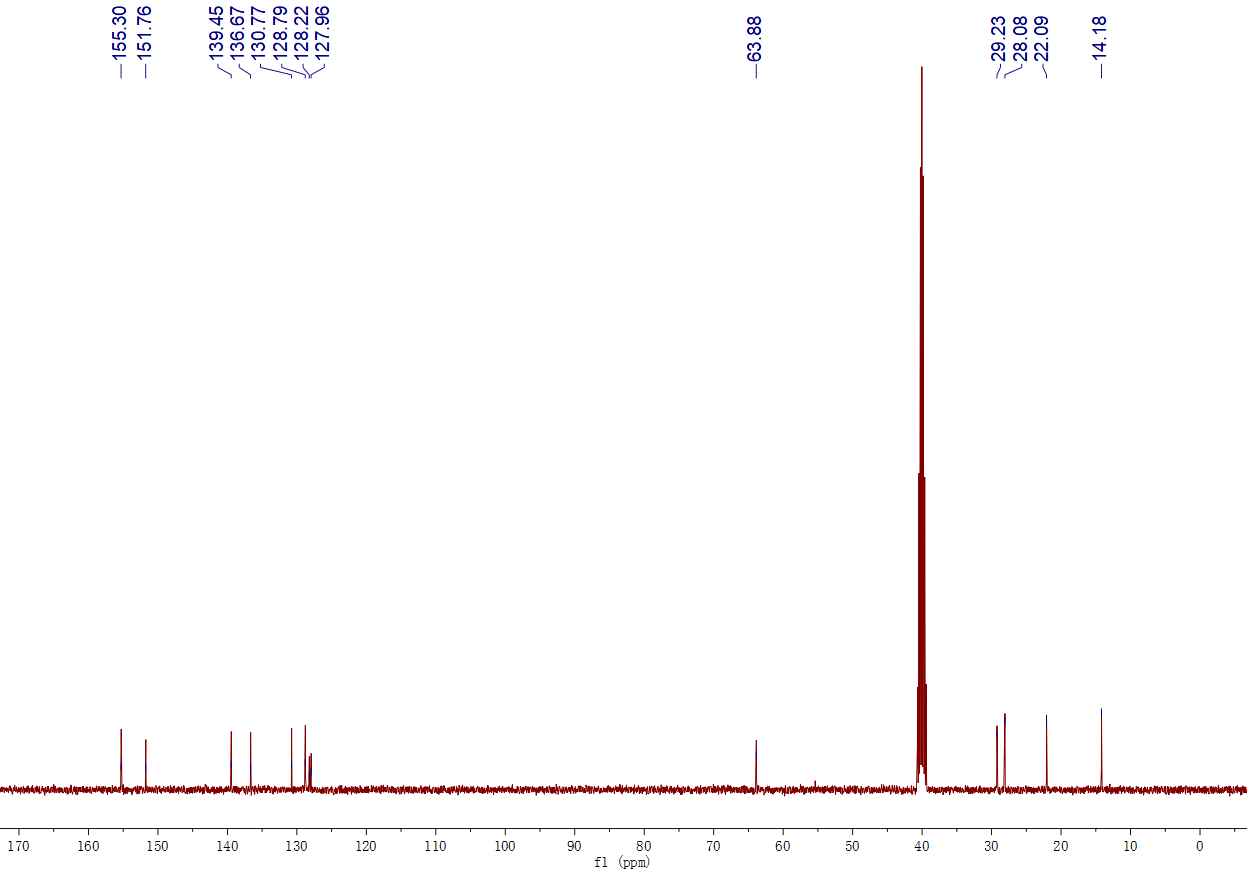

**Figure S5**. The NMR, HRMS and LC data of compound **2e**.


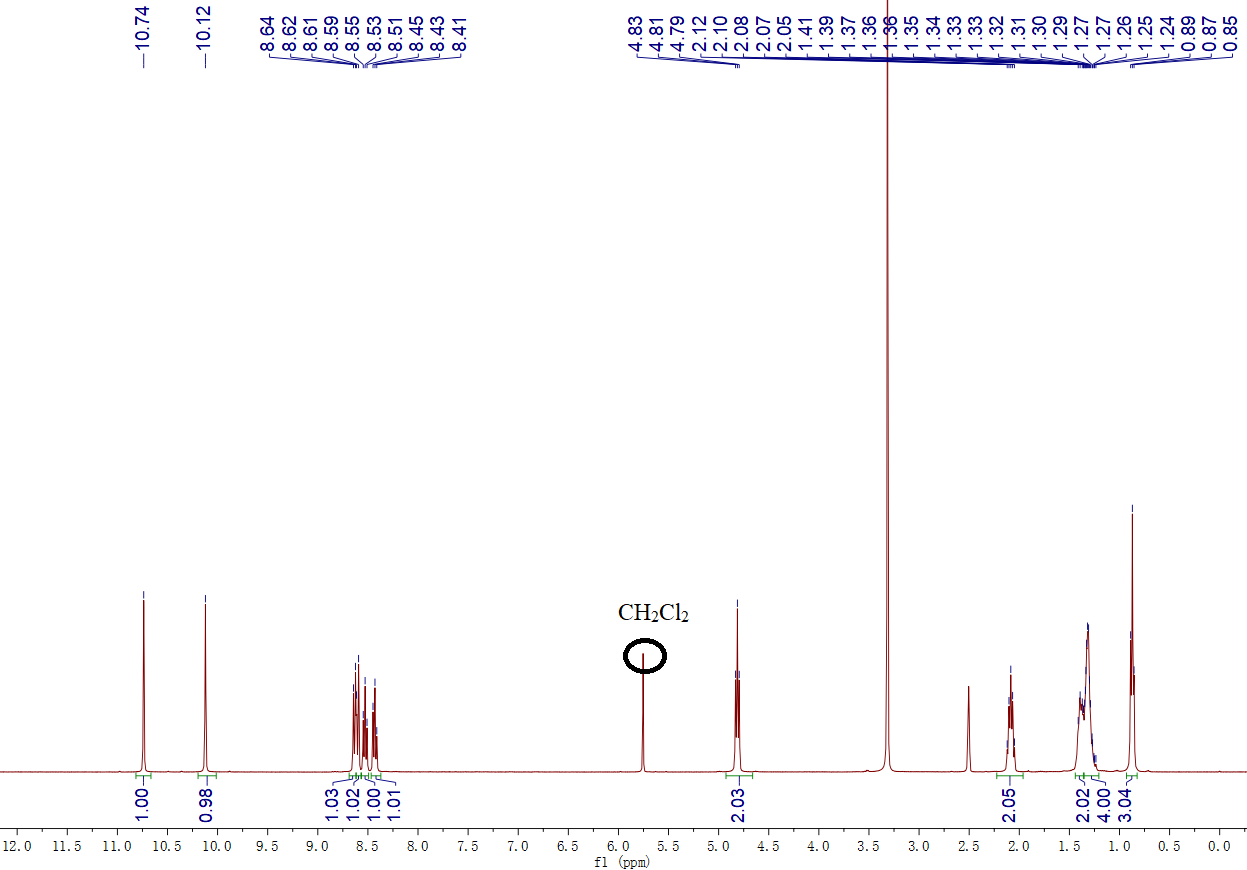


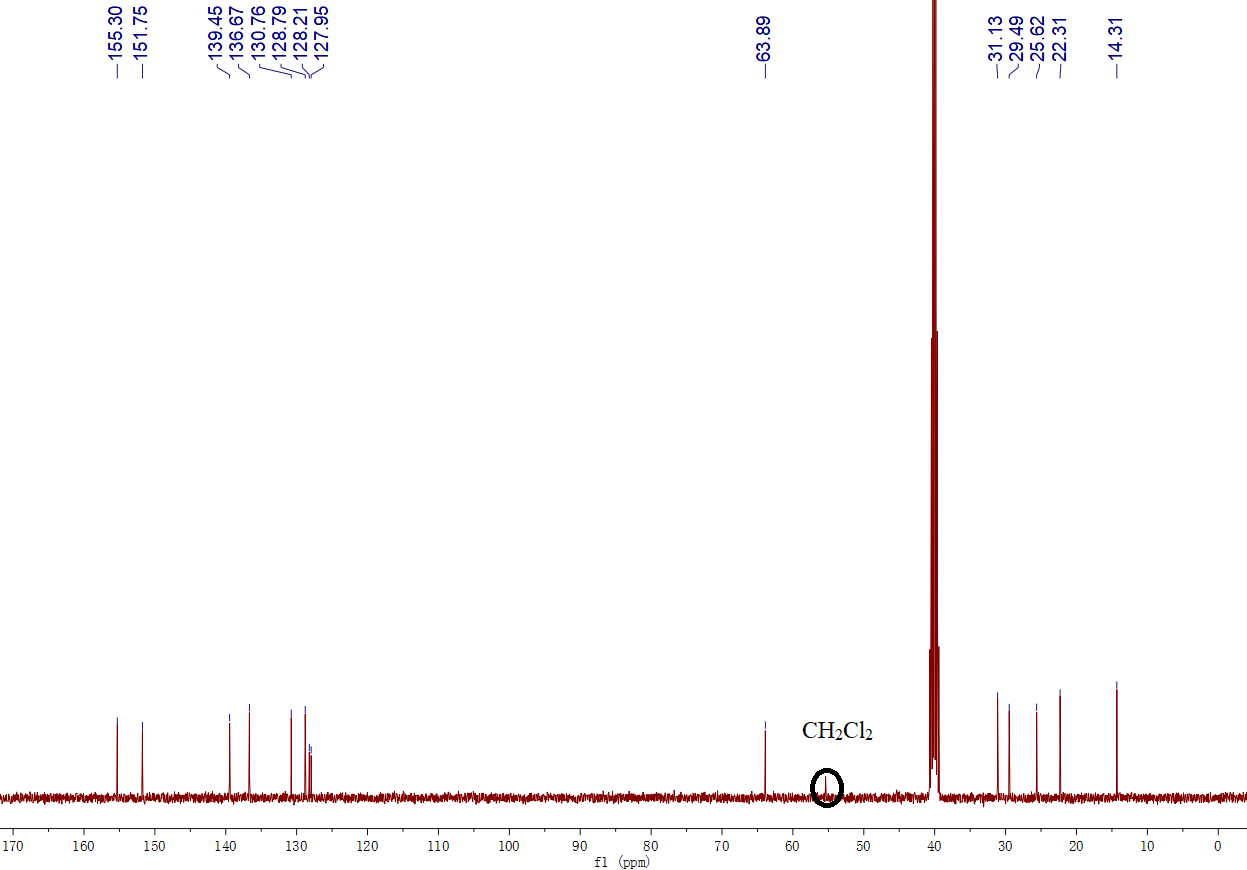

**Figure S6**. The NMR, HRMS and LC data of compound **2f**.


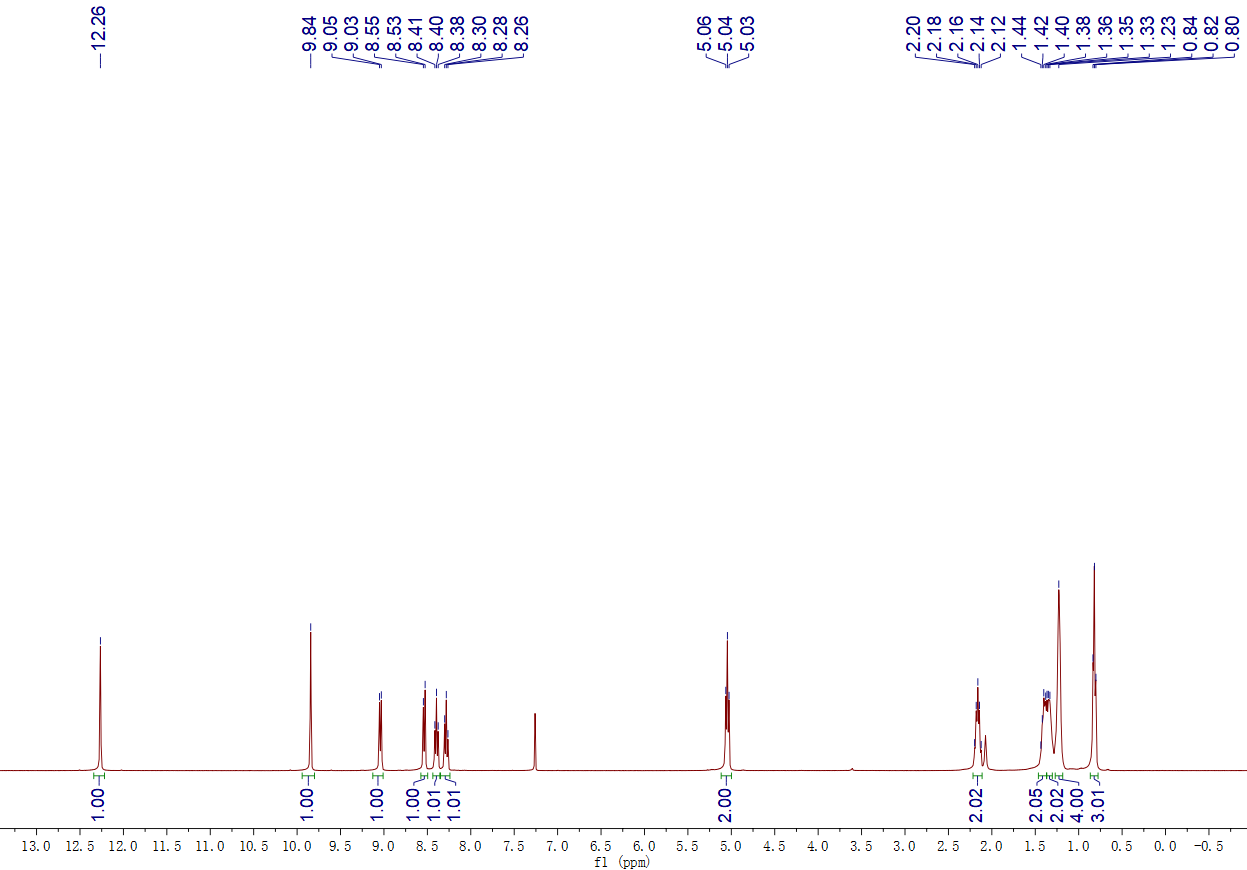


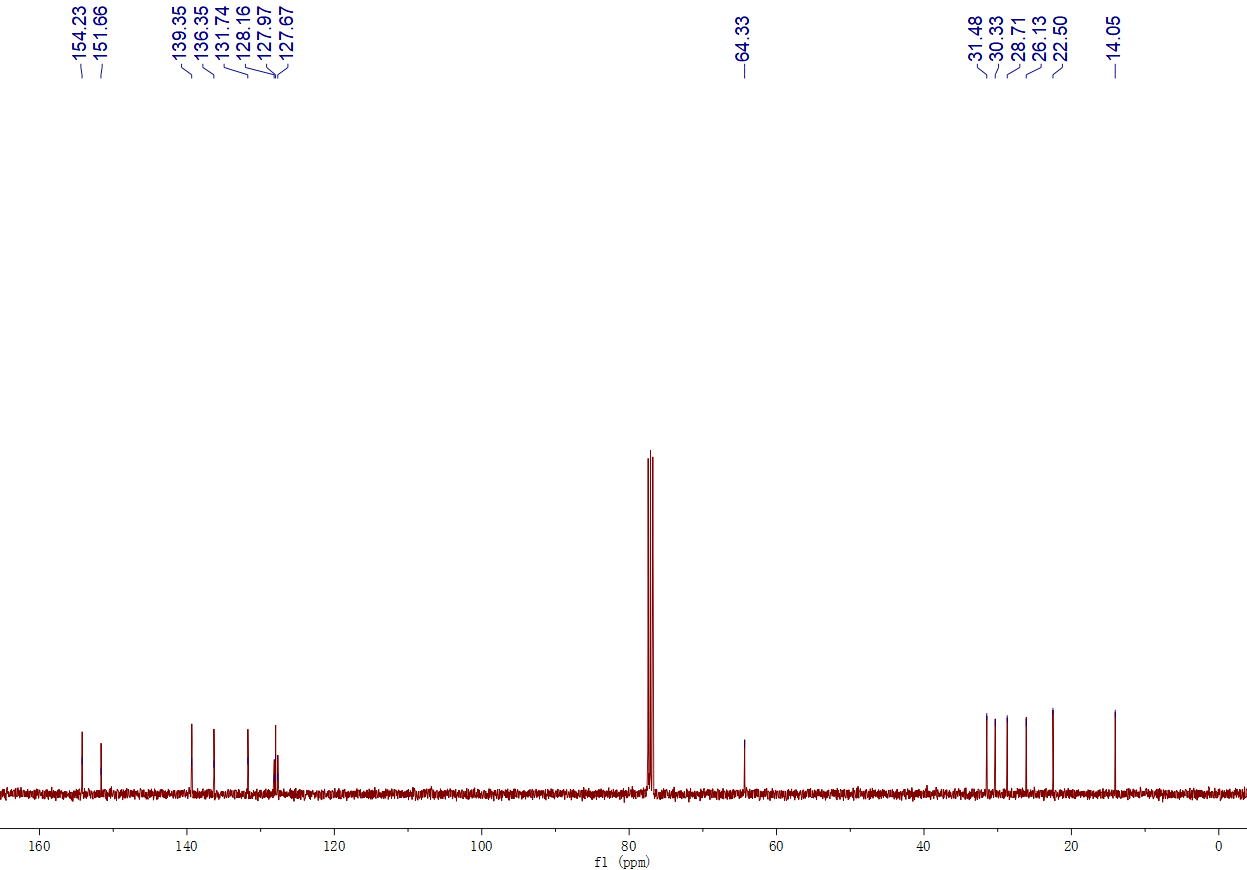

**Figure S7**. The NMR, HRMS and LC data of compound **2g**.


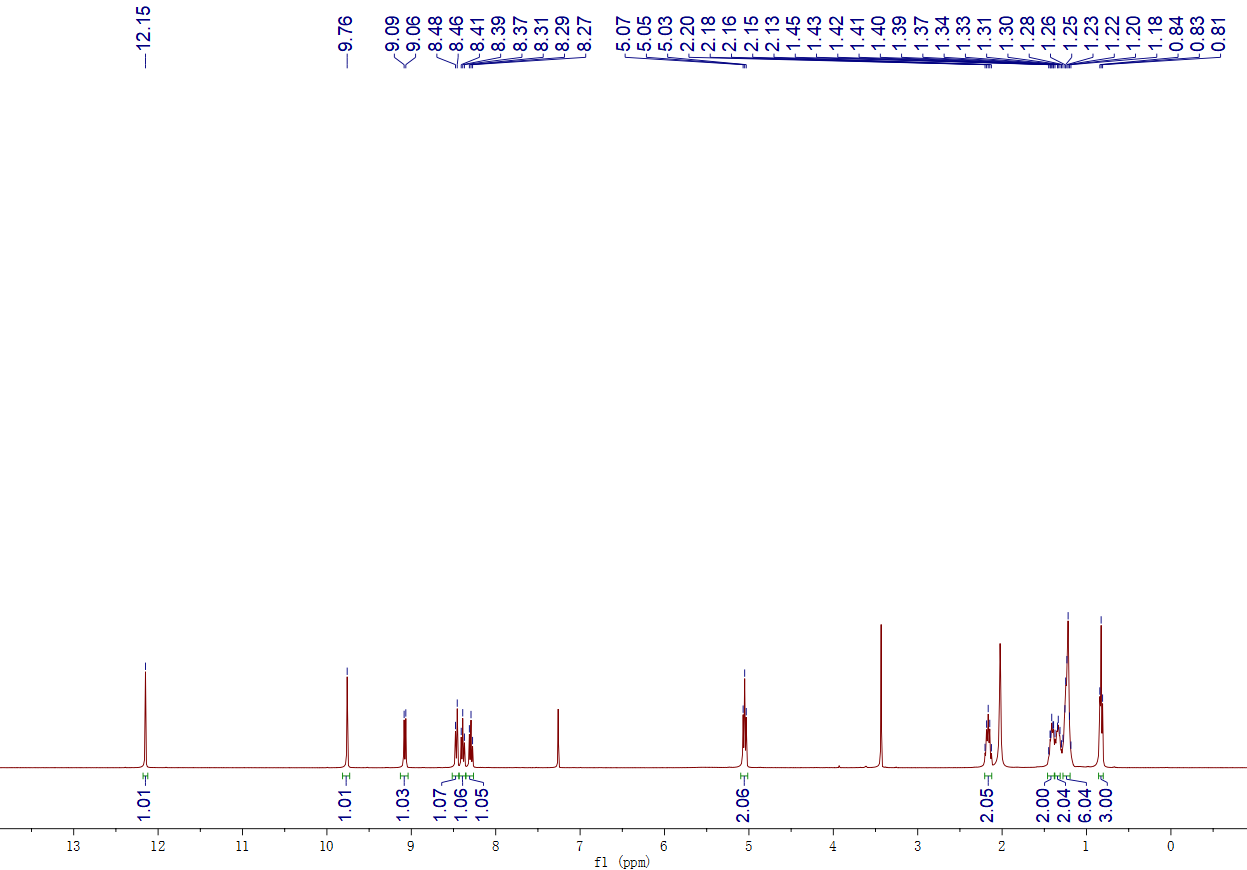


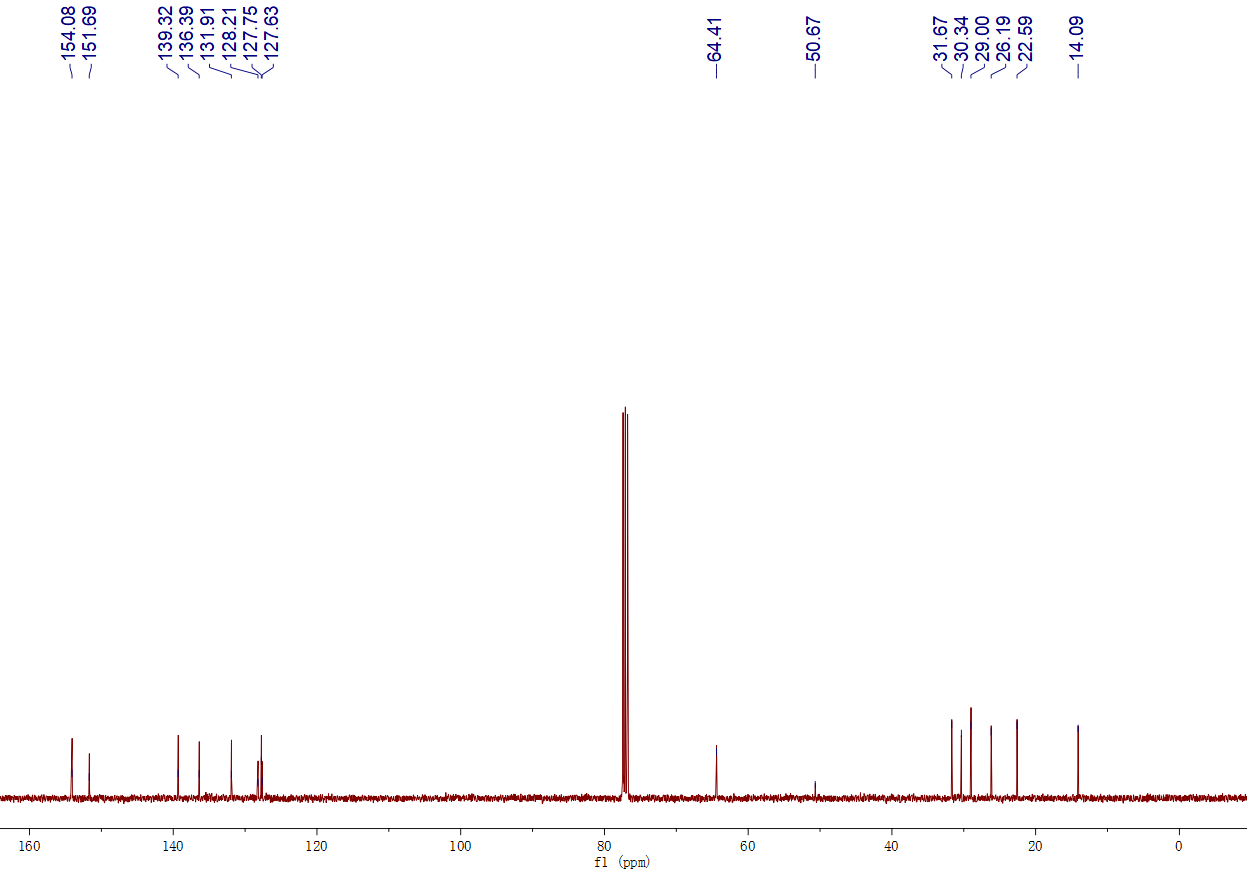

**Figure S8**. The NMR, HRMS and LC data of compound **2h**.


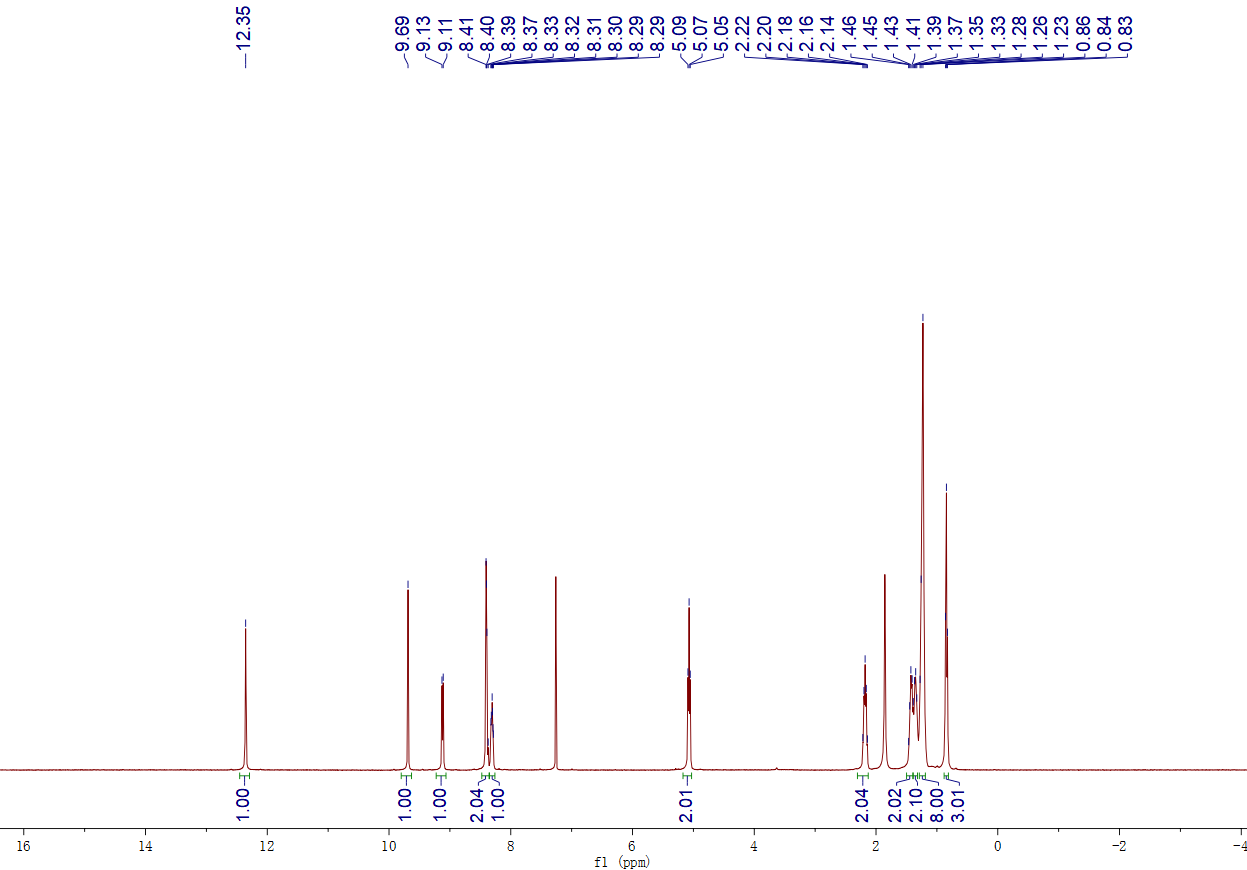


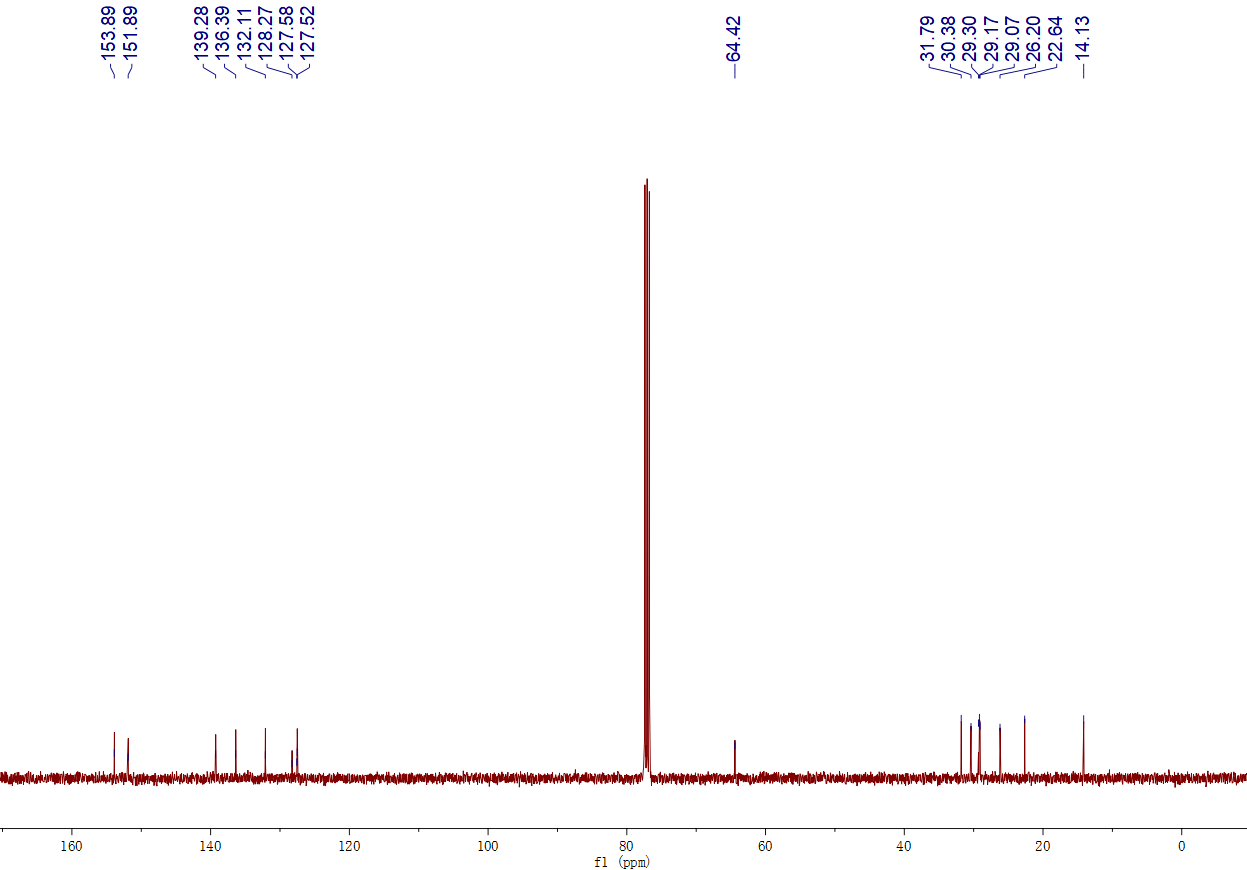

**Figure S9**. The NMR, HRMS and LC data of compound **2i**.


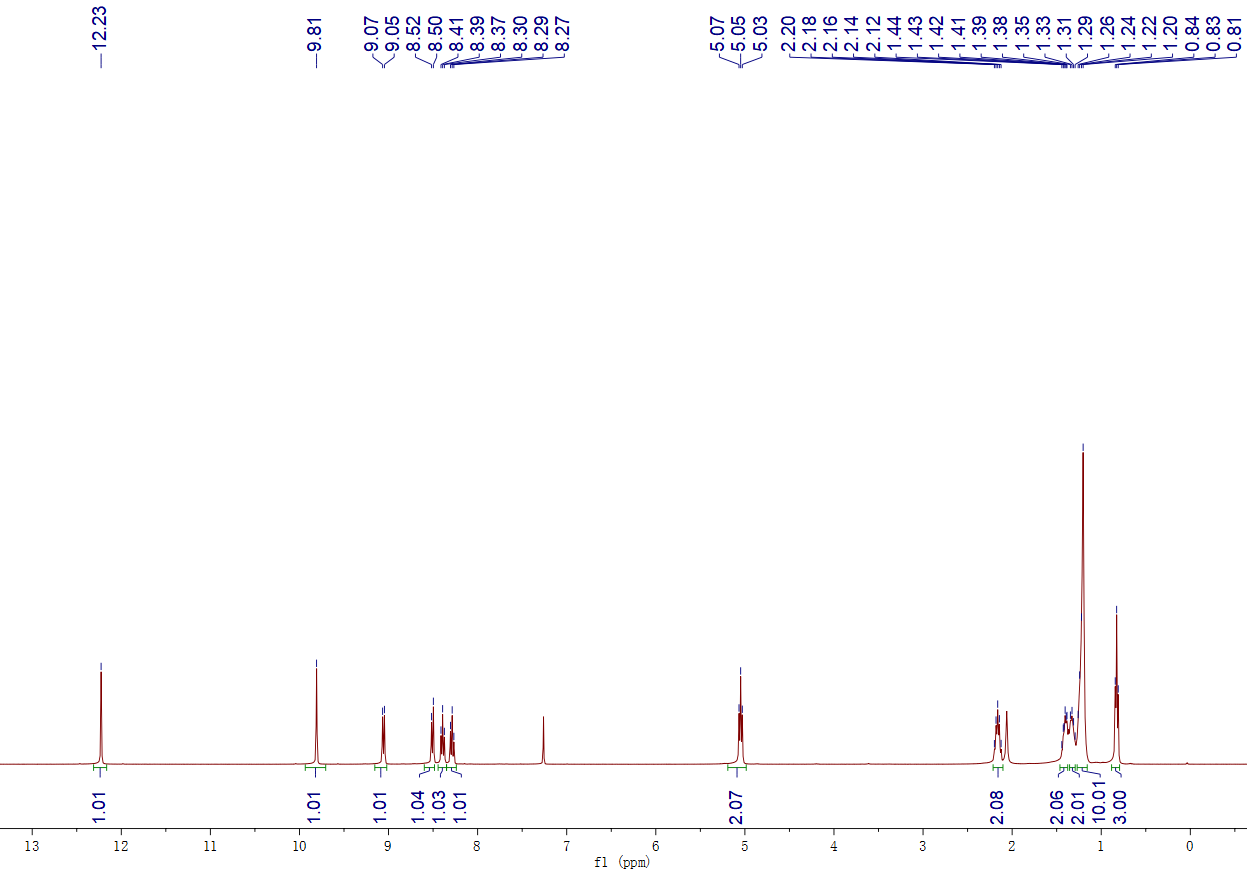


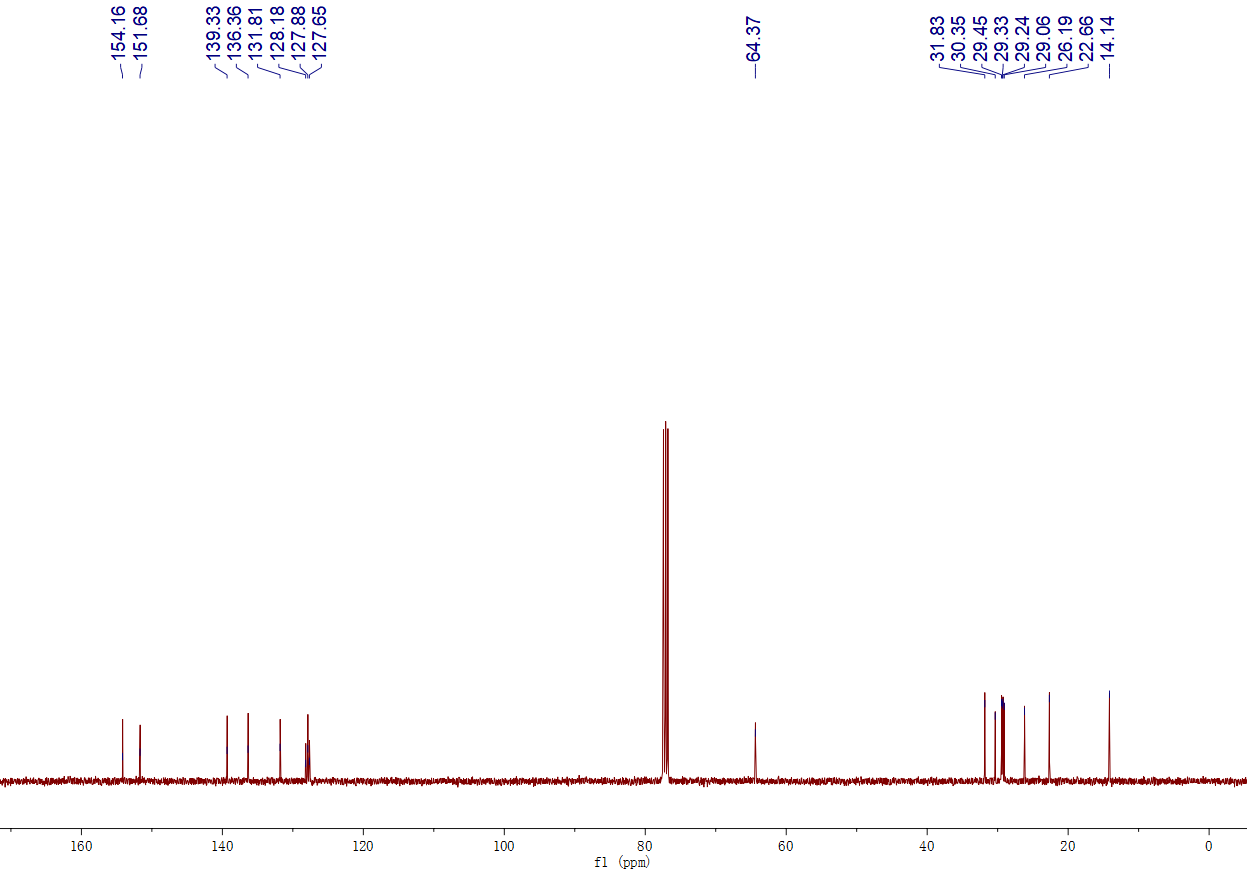

**Figure S10**. The NMR, HRMS and LC data of compound **2j**.


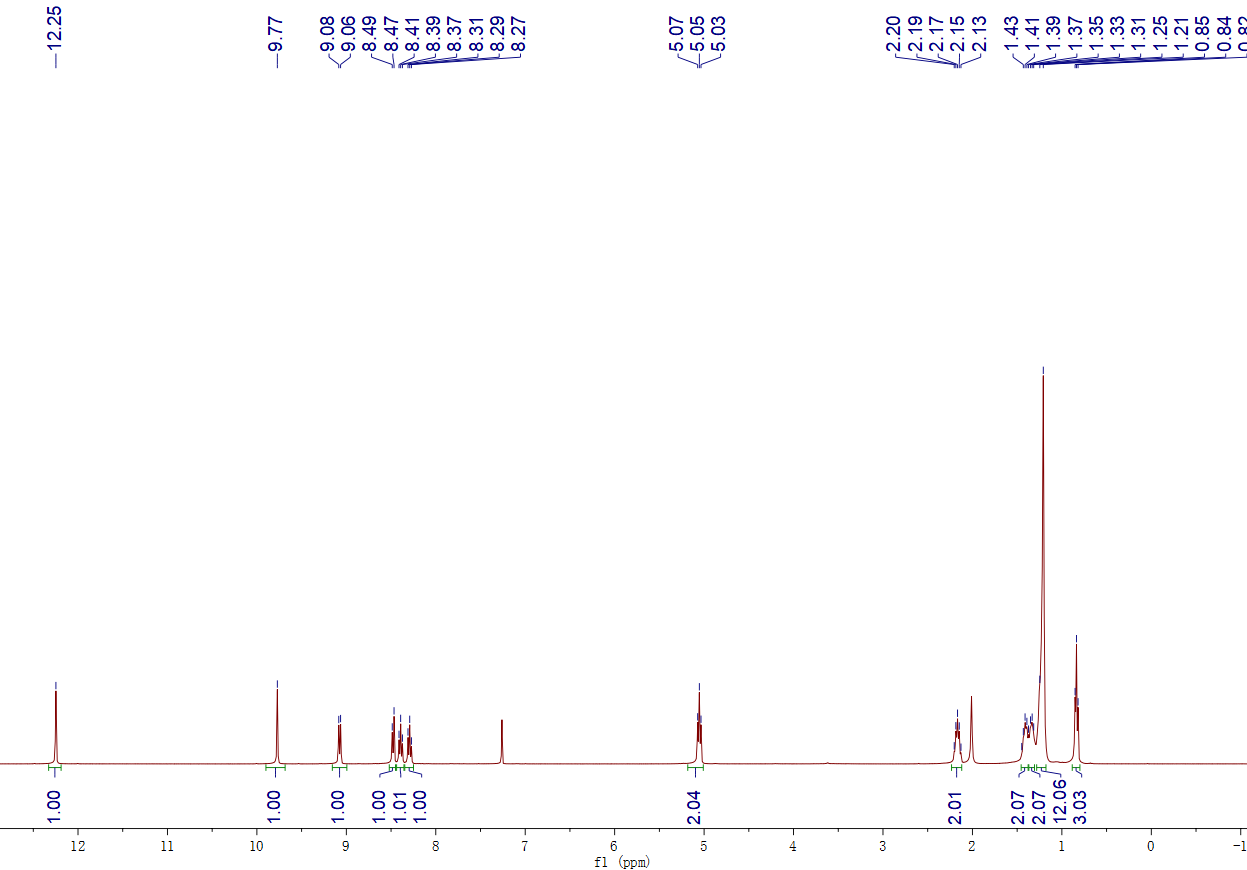


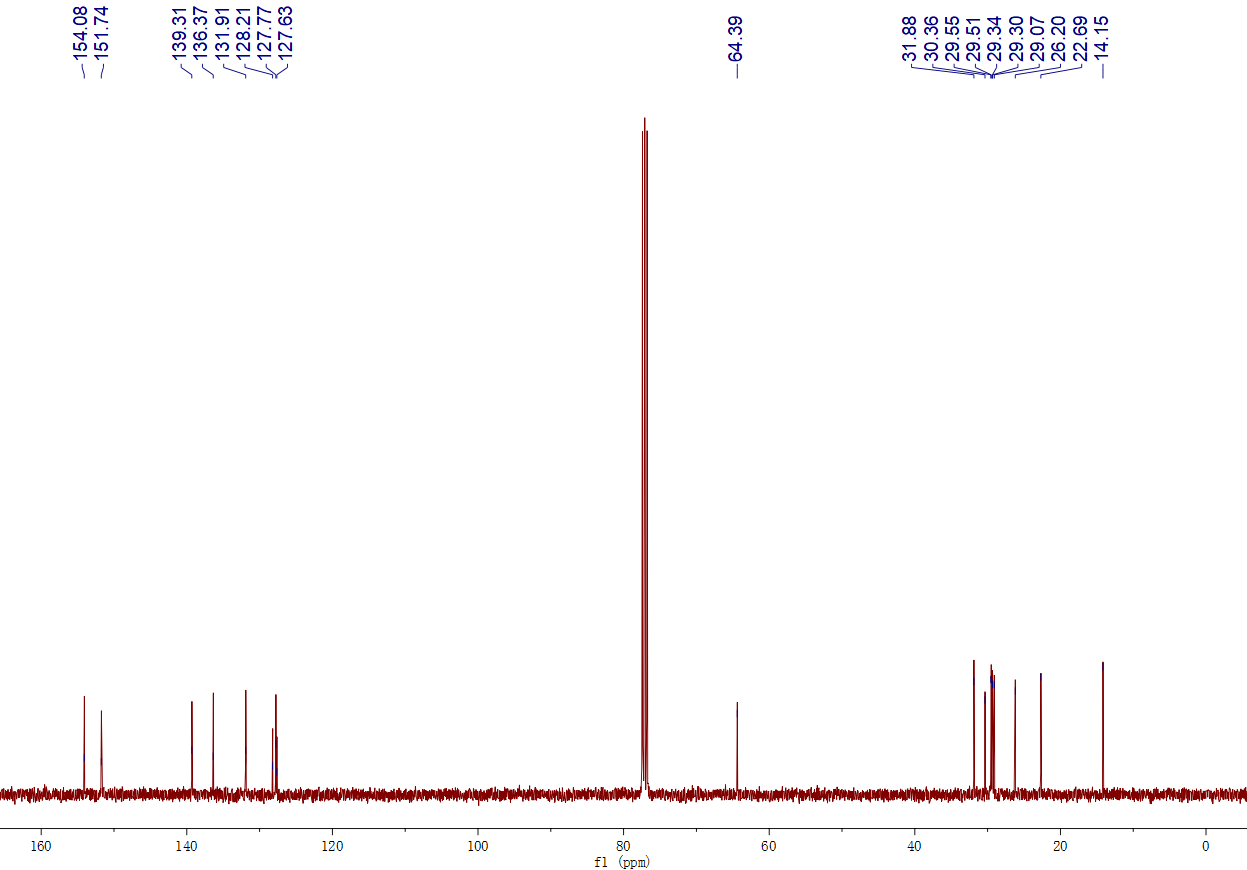

**Figure S11**. The NMR, HRMS and LC data of compound **2k**.


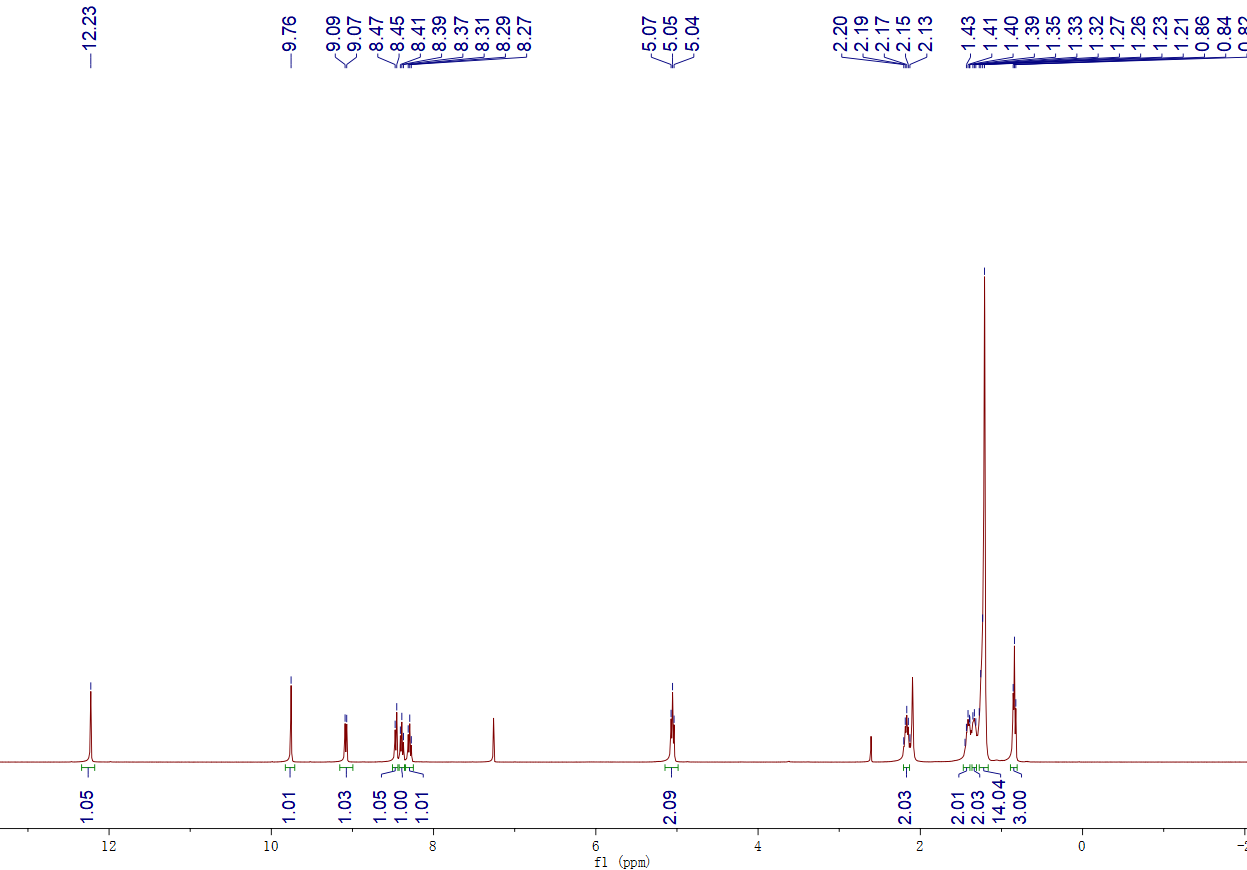


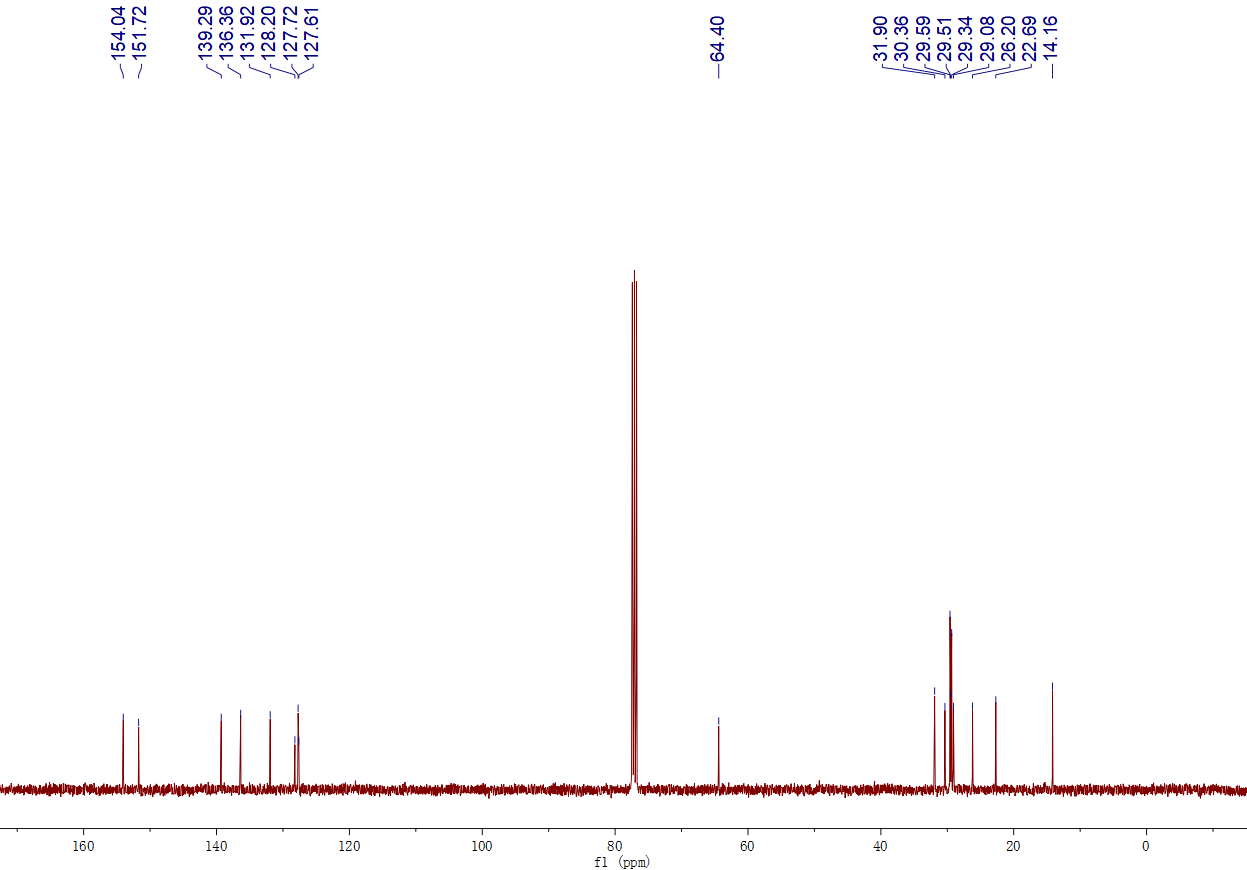

**Figure S12**. The NMR, HRMS and LC data of compound **2l**.


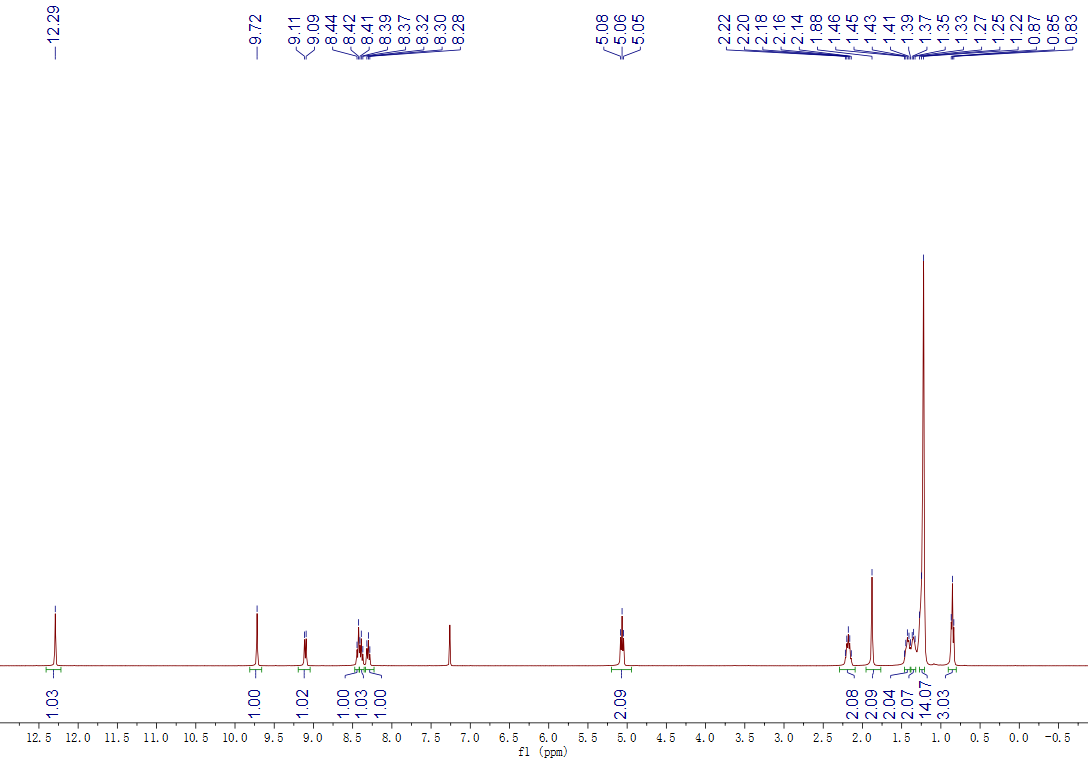


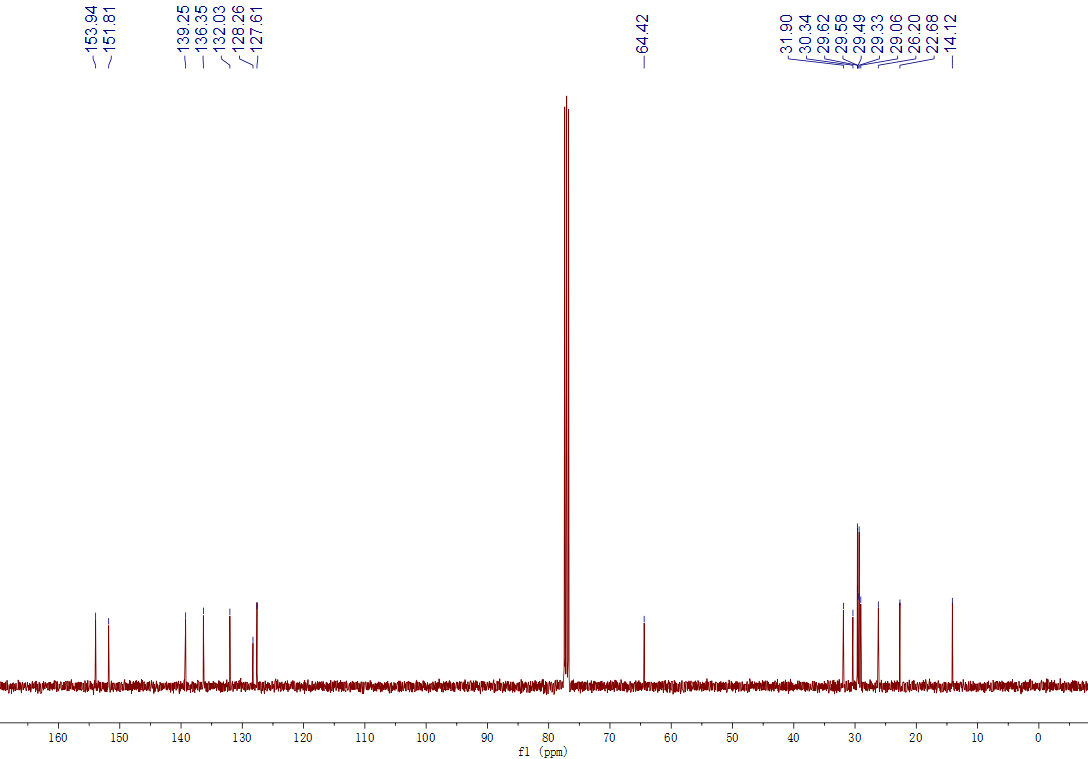

**Figure S13**. The NMR, HRMS and LC data of compound **2m**.


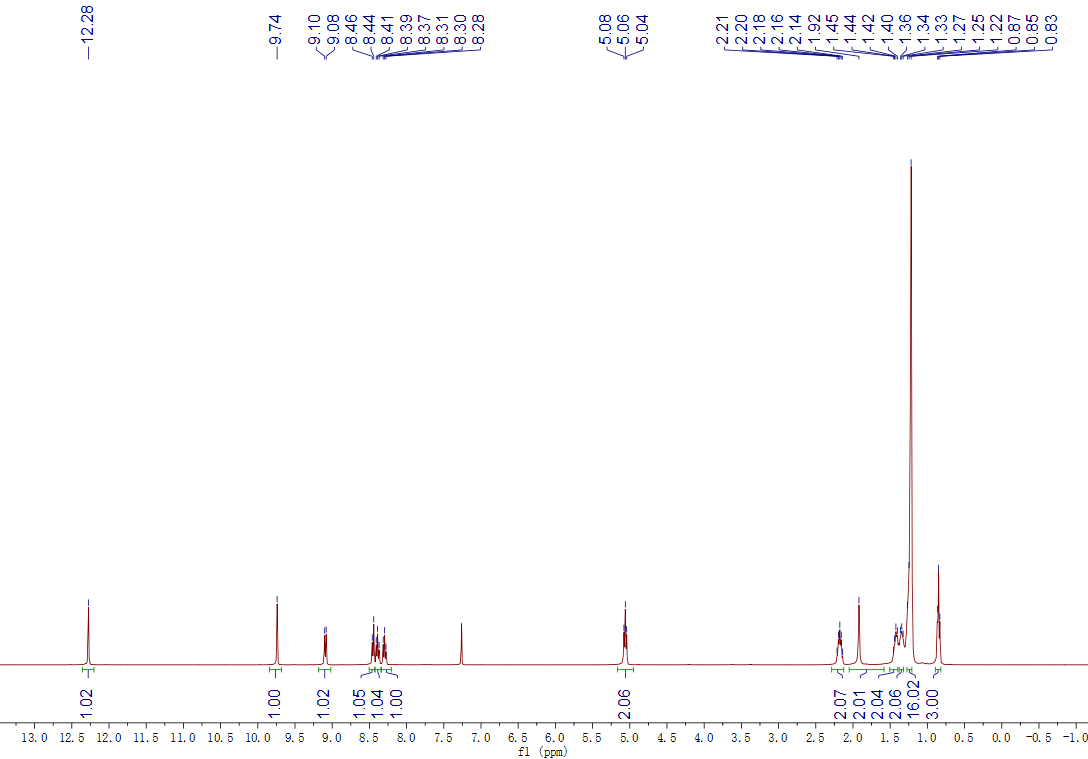


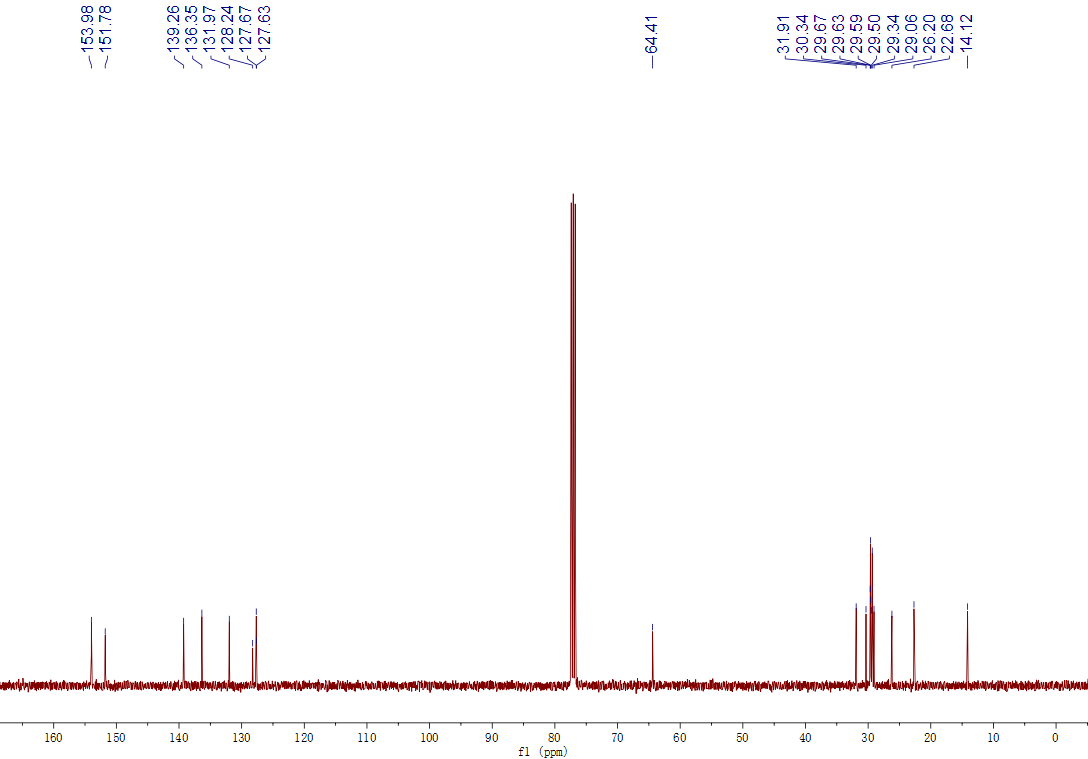

**Figure S14**. The NMR, HRMS and LC data of compound **2n**.


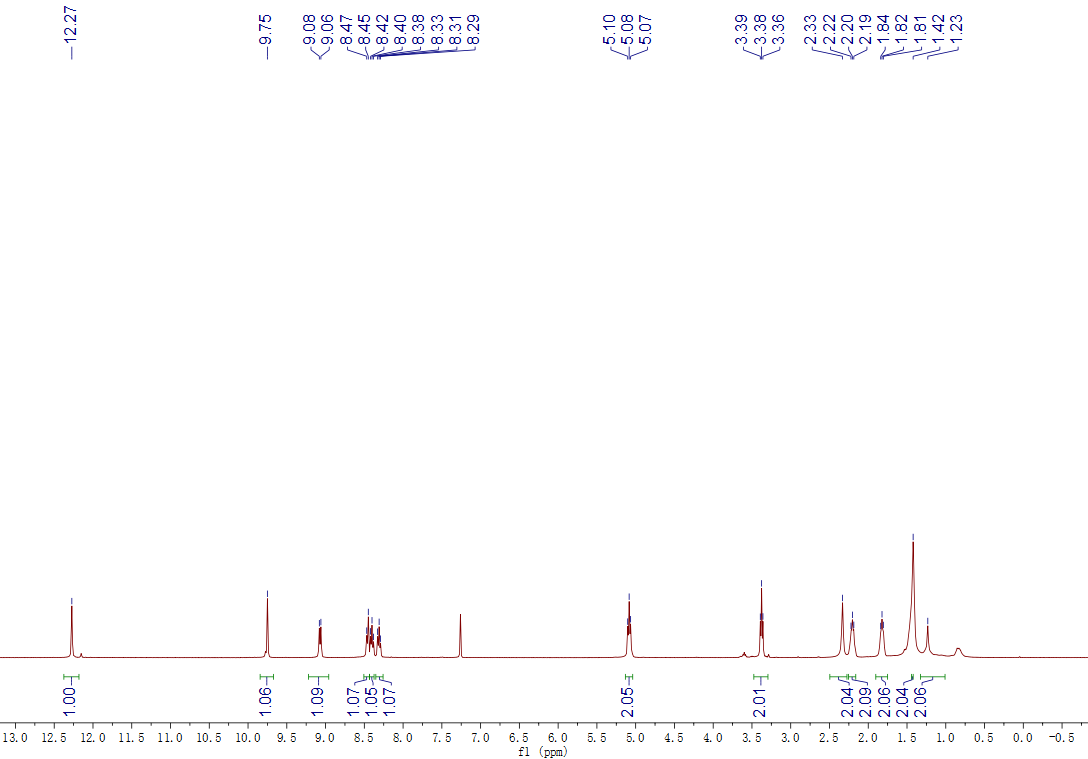


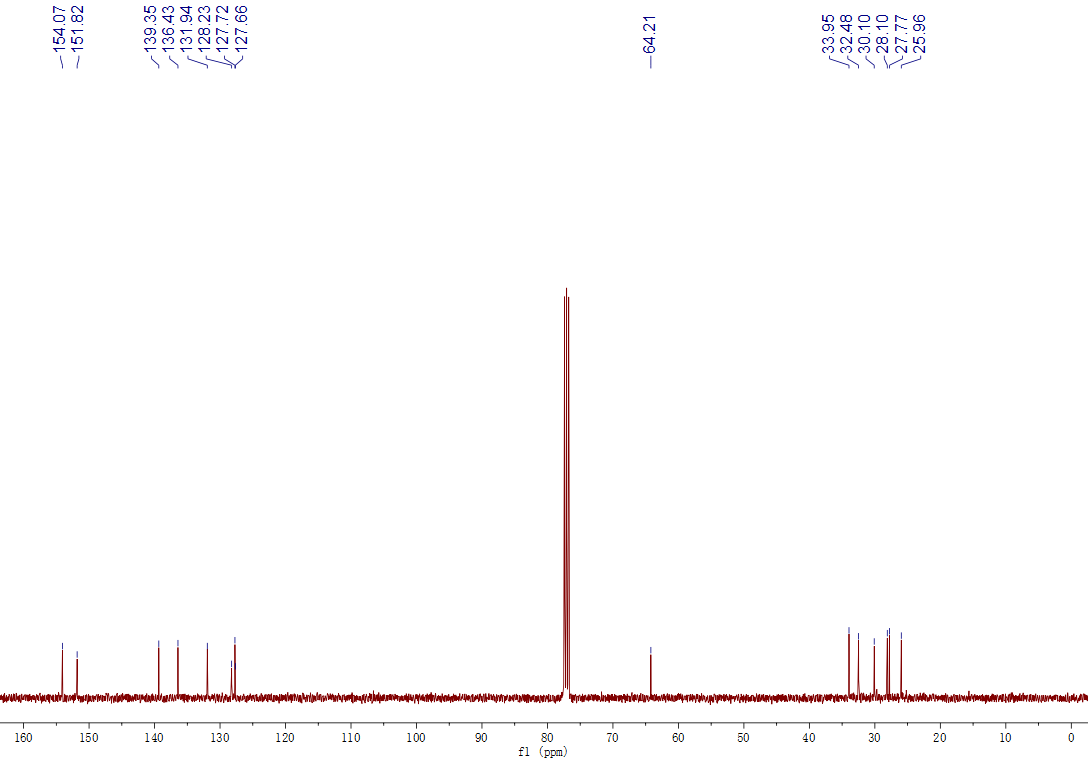

**Figure S15**. The NMR, HRMS and LC data of compound **2o**.


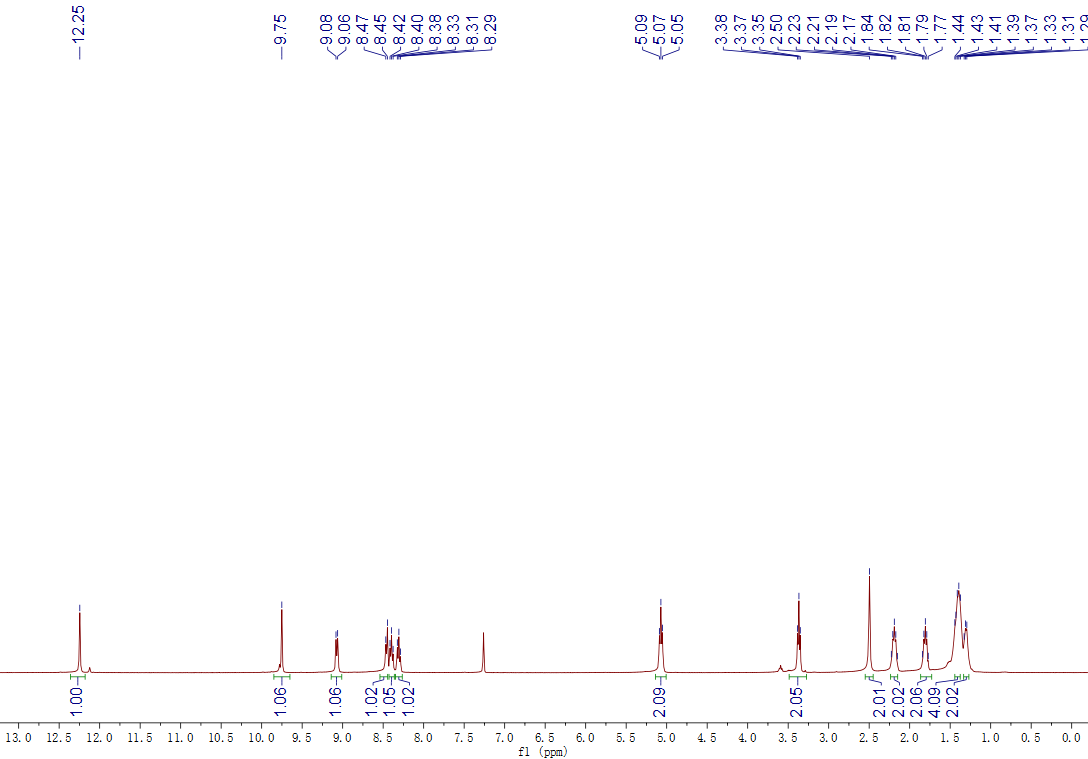


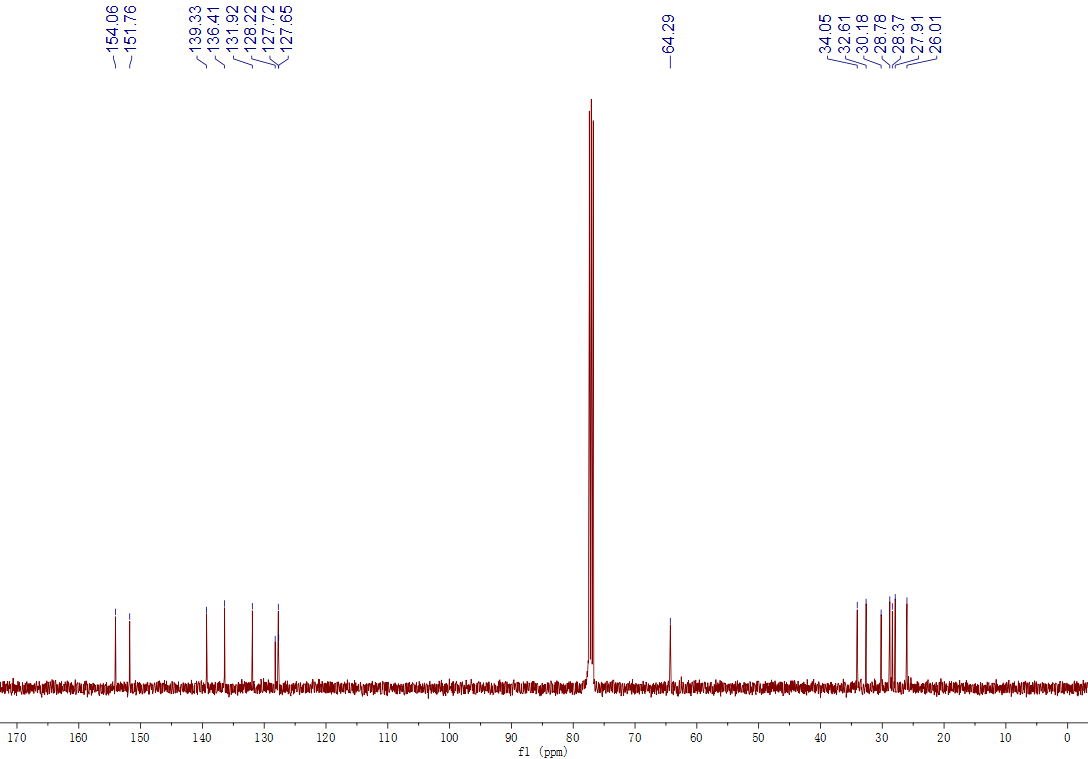

**Figure S16**. The NMR, HRMS and LC data of compound **2p**.


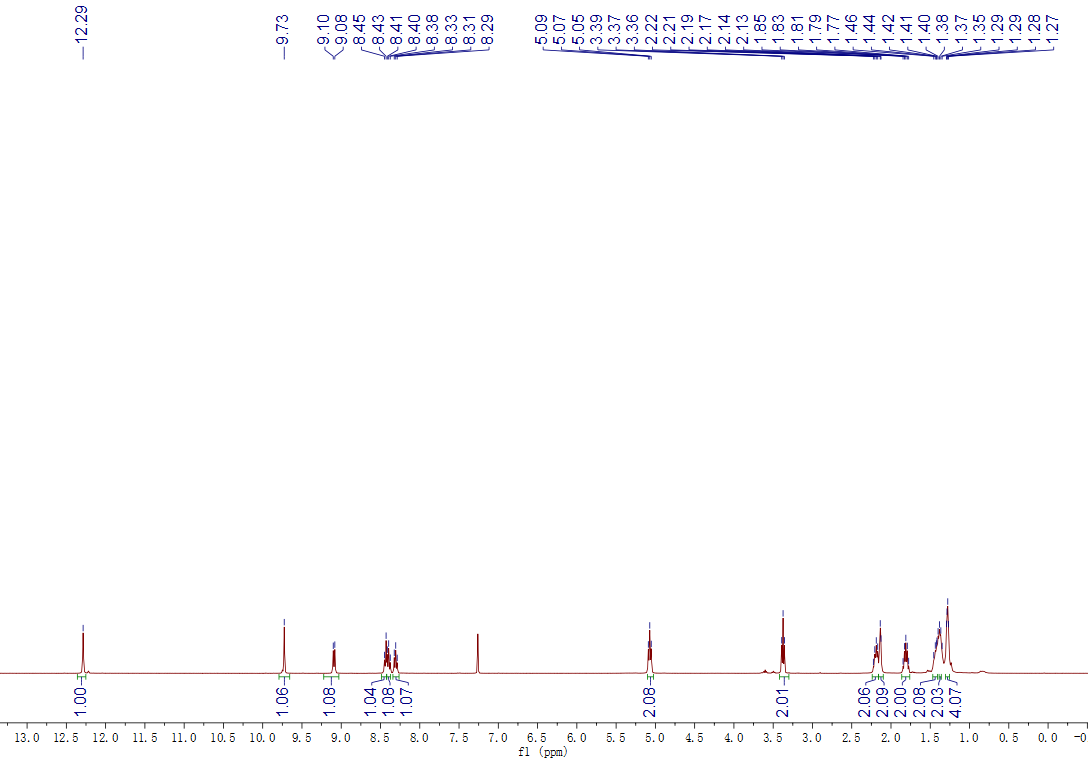


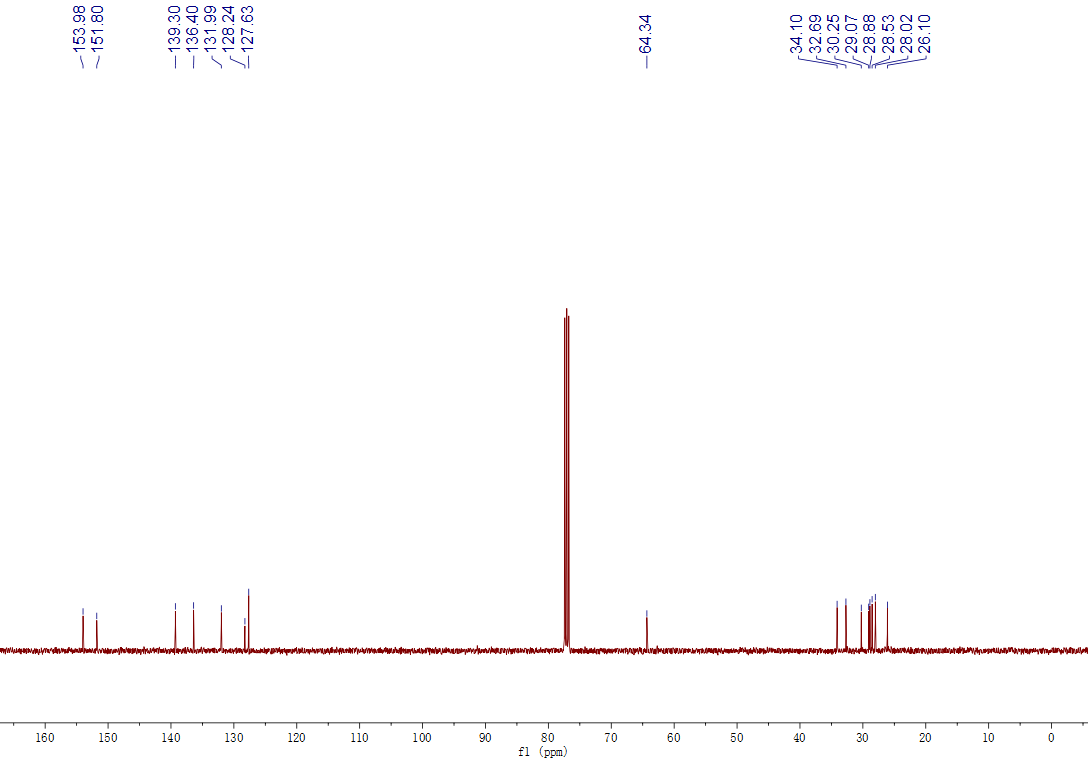

**Figure S17**. The NMR, HRMS and LC data of compound **2q**.


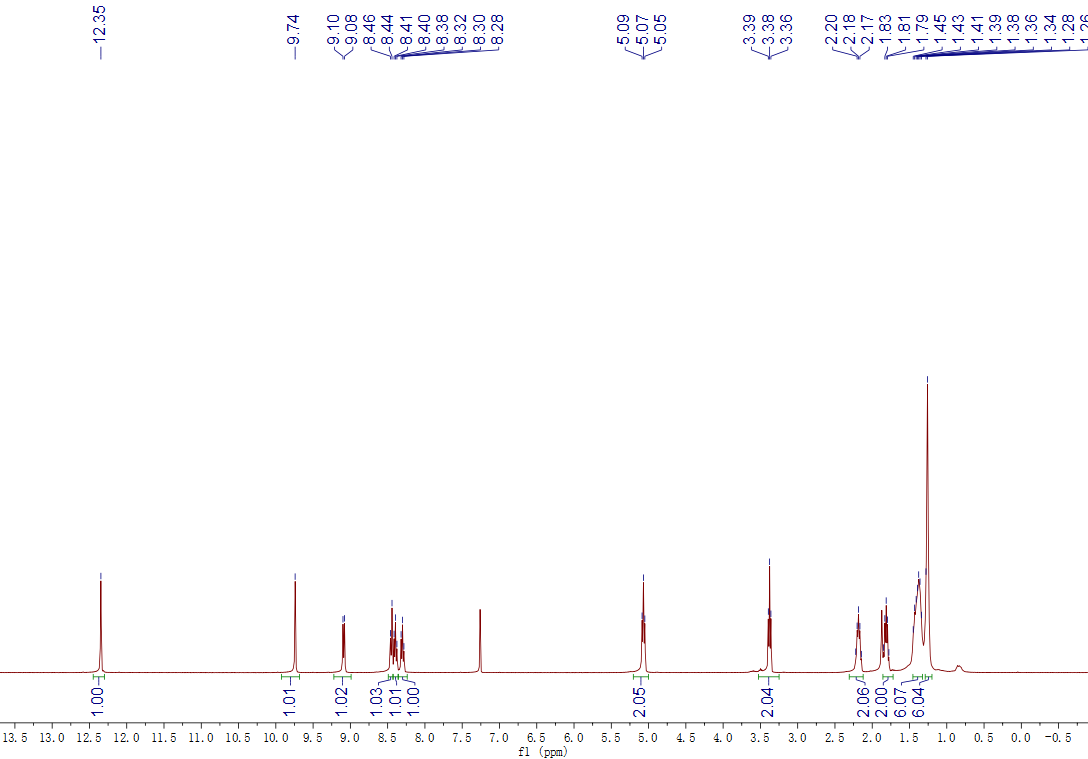


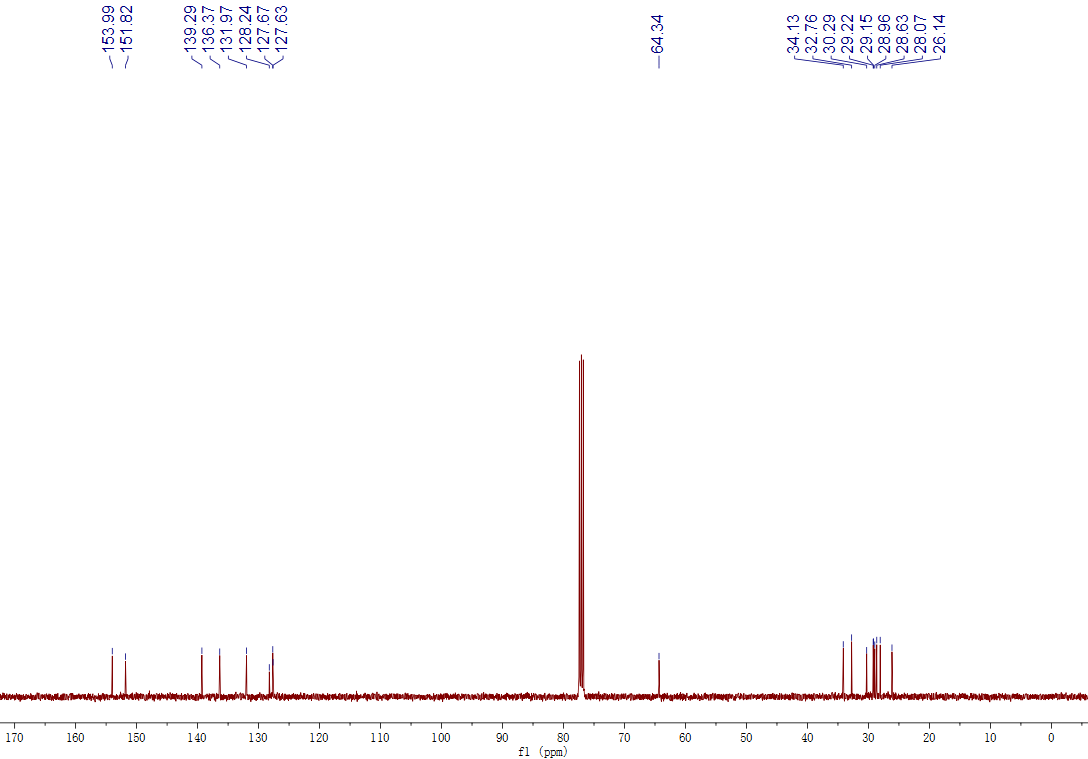

**Figure S18**. The NMR, HRMS and LC data of compound **2r**.


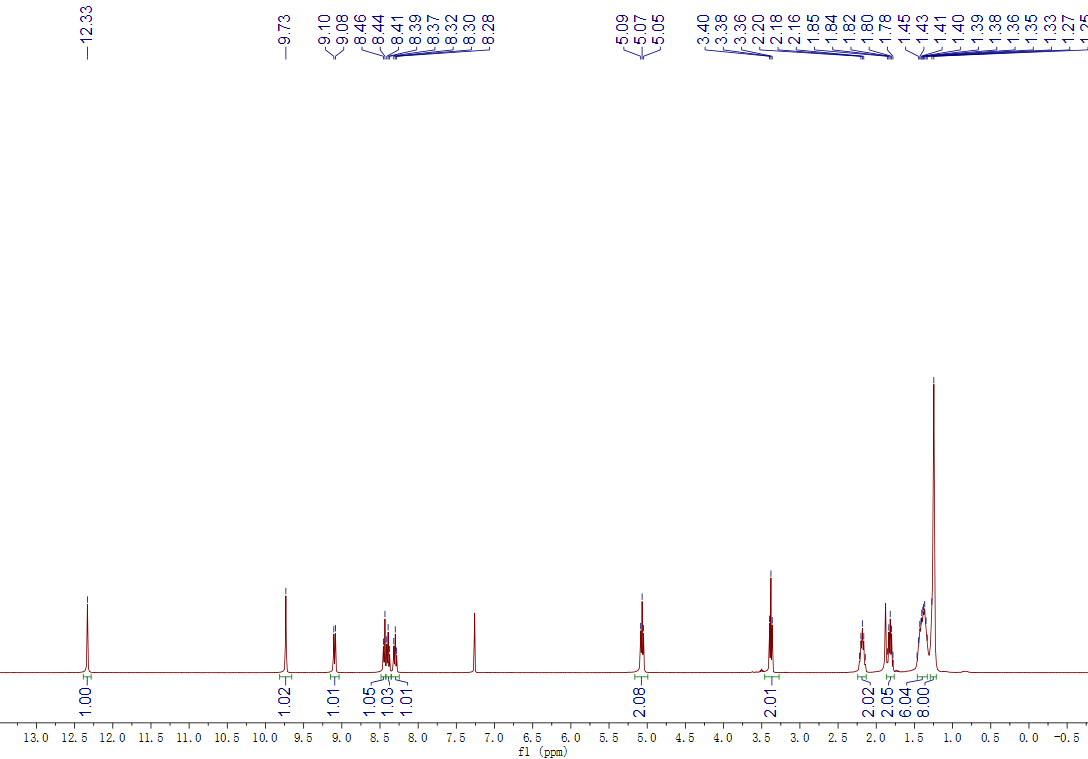


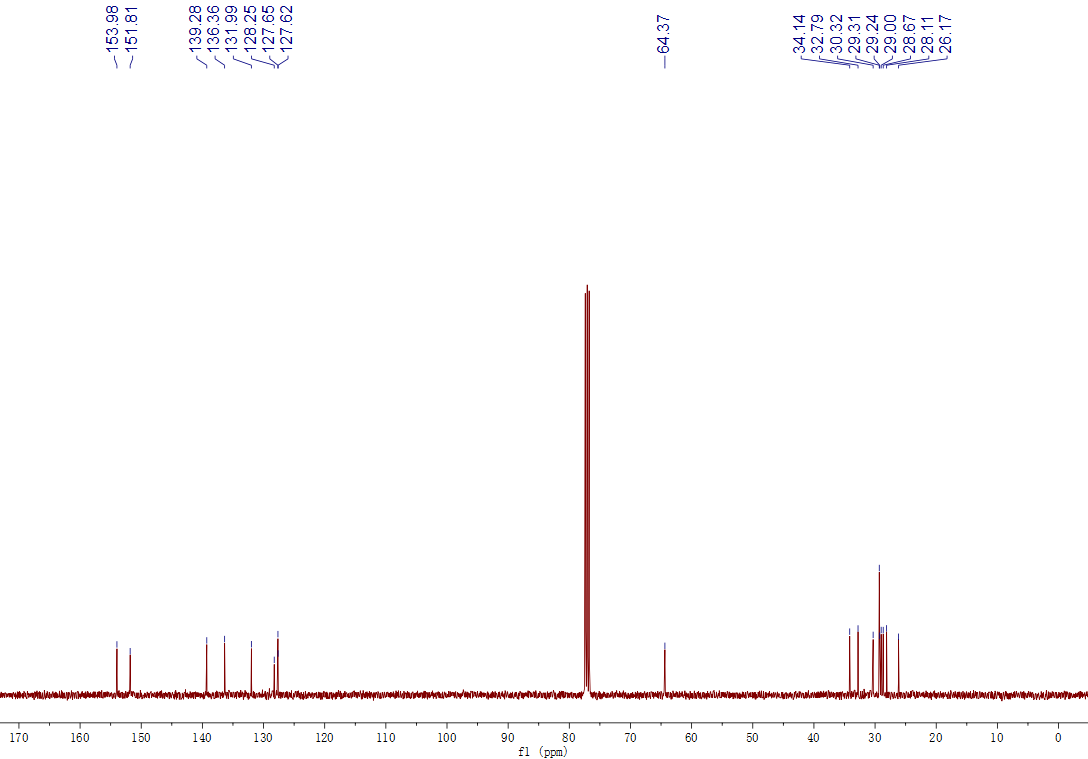

**Figure S19**. The NMR, HRMS and LC data of compound **2s**.


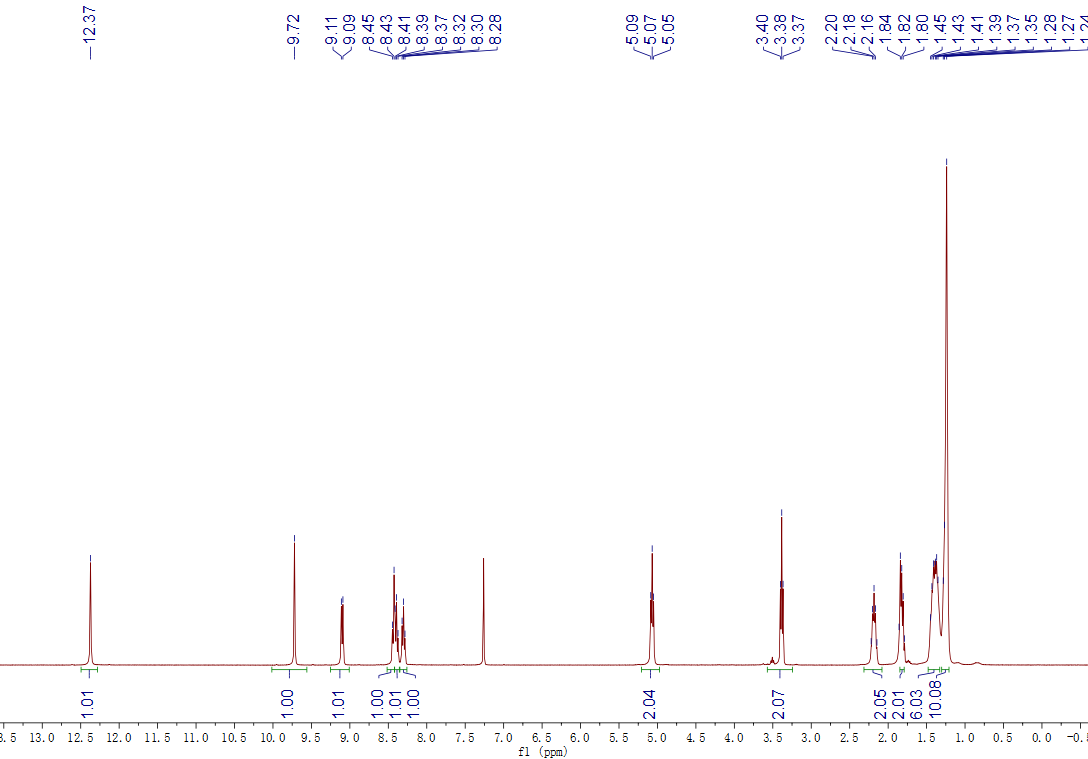


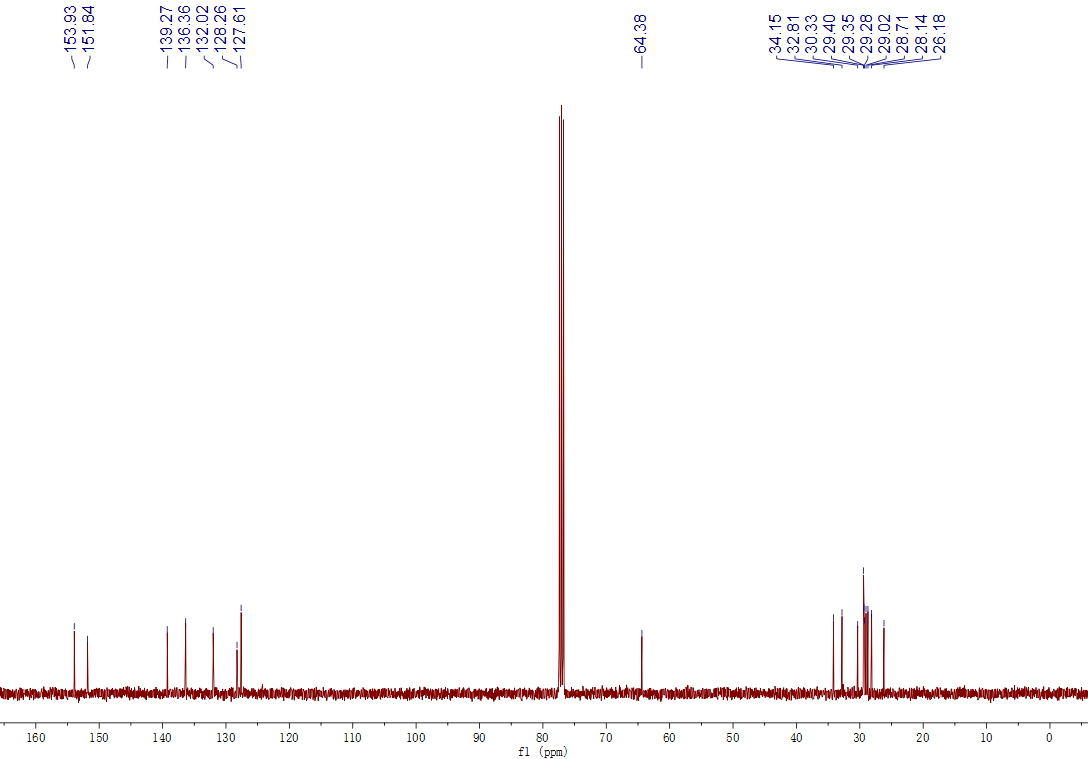

**Figure S20**. The NMR, HRMS and LC data of compound **2t**.


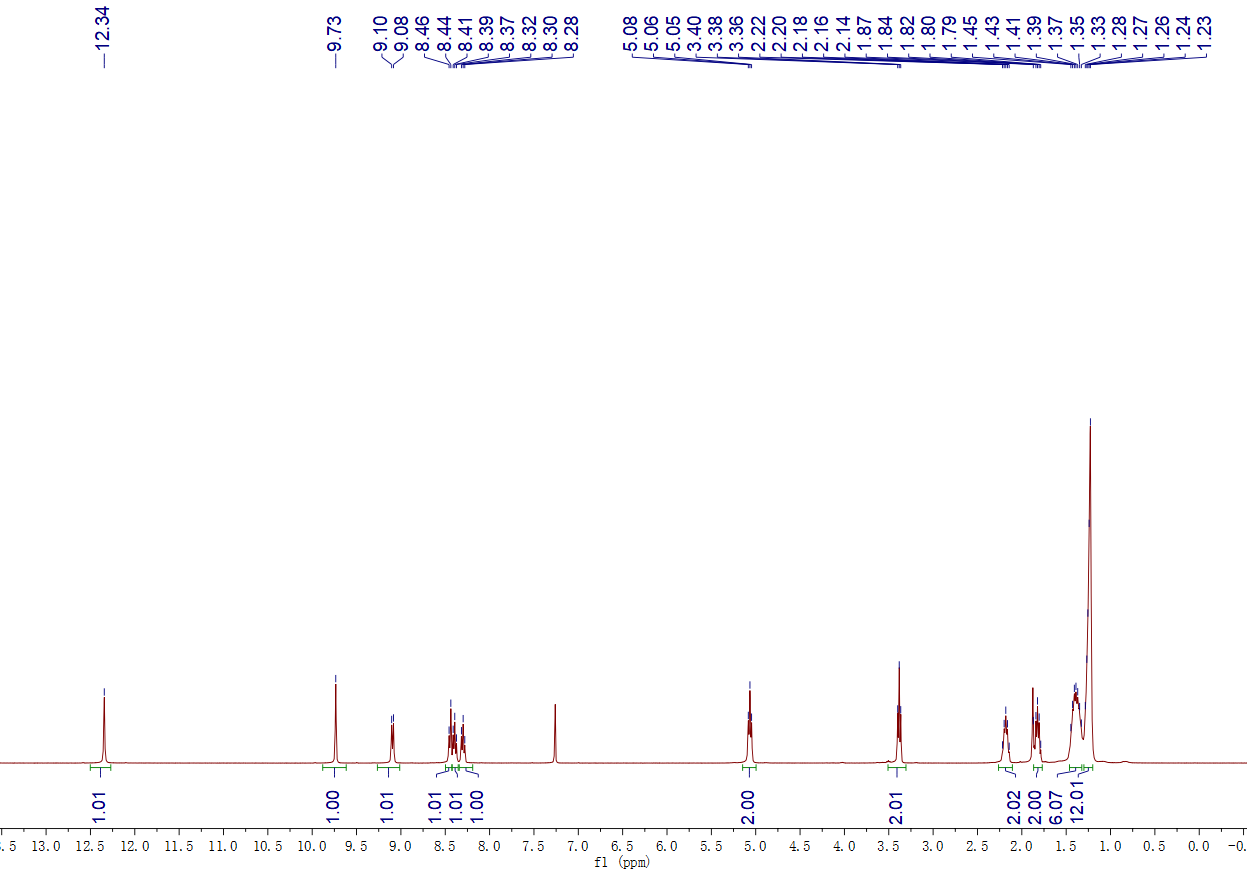


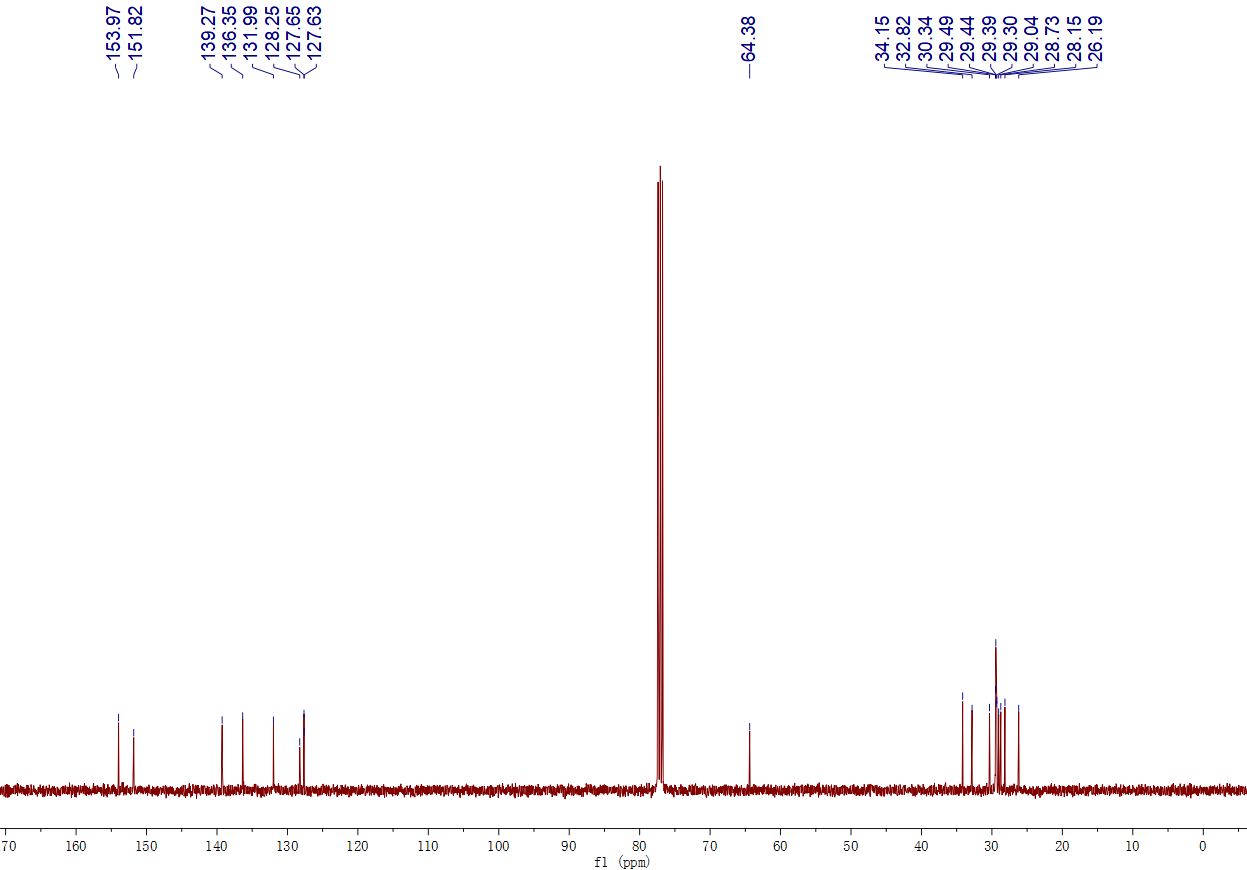

**Figure S21**. The NMR, HRMS and LC data of compound **2u**.


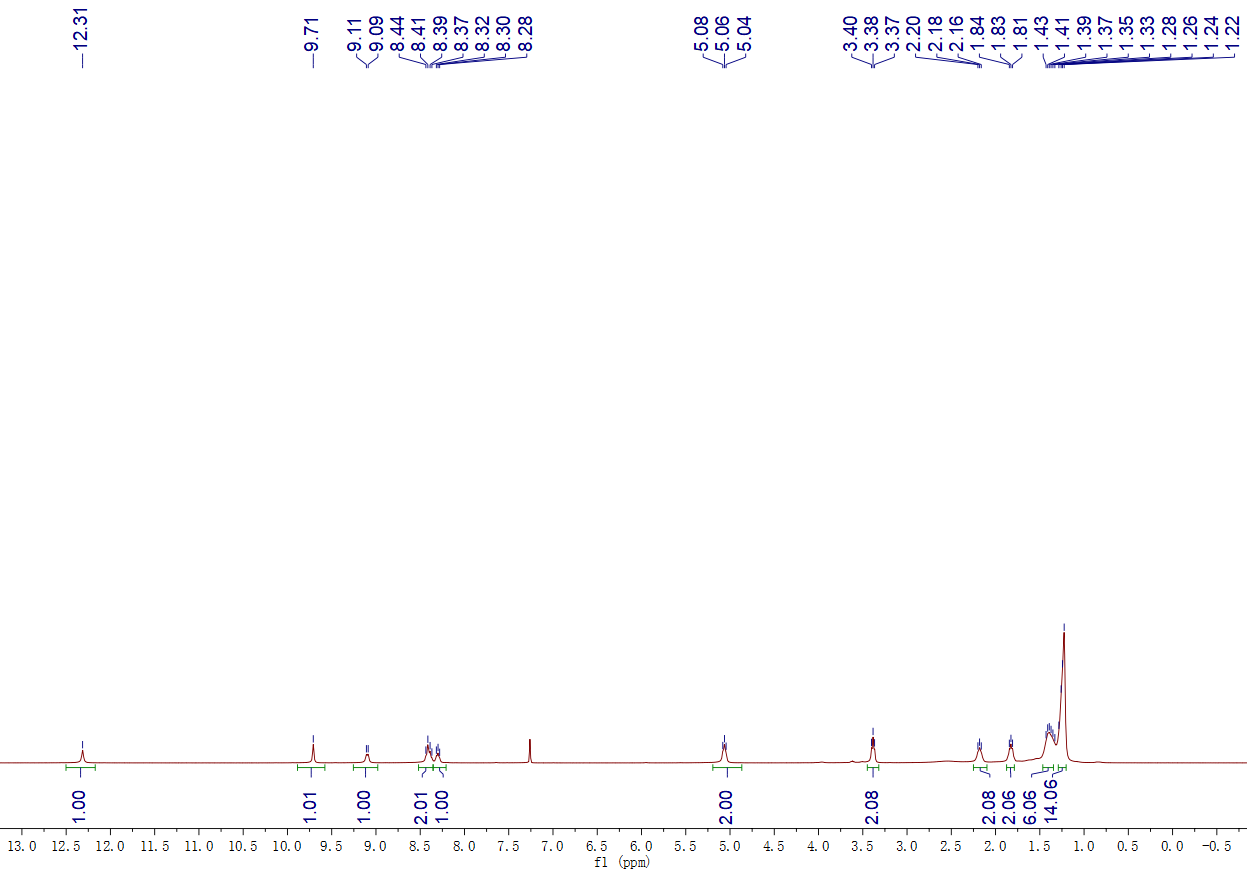


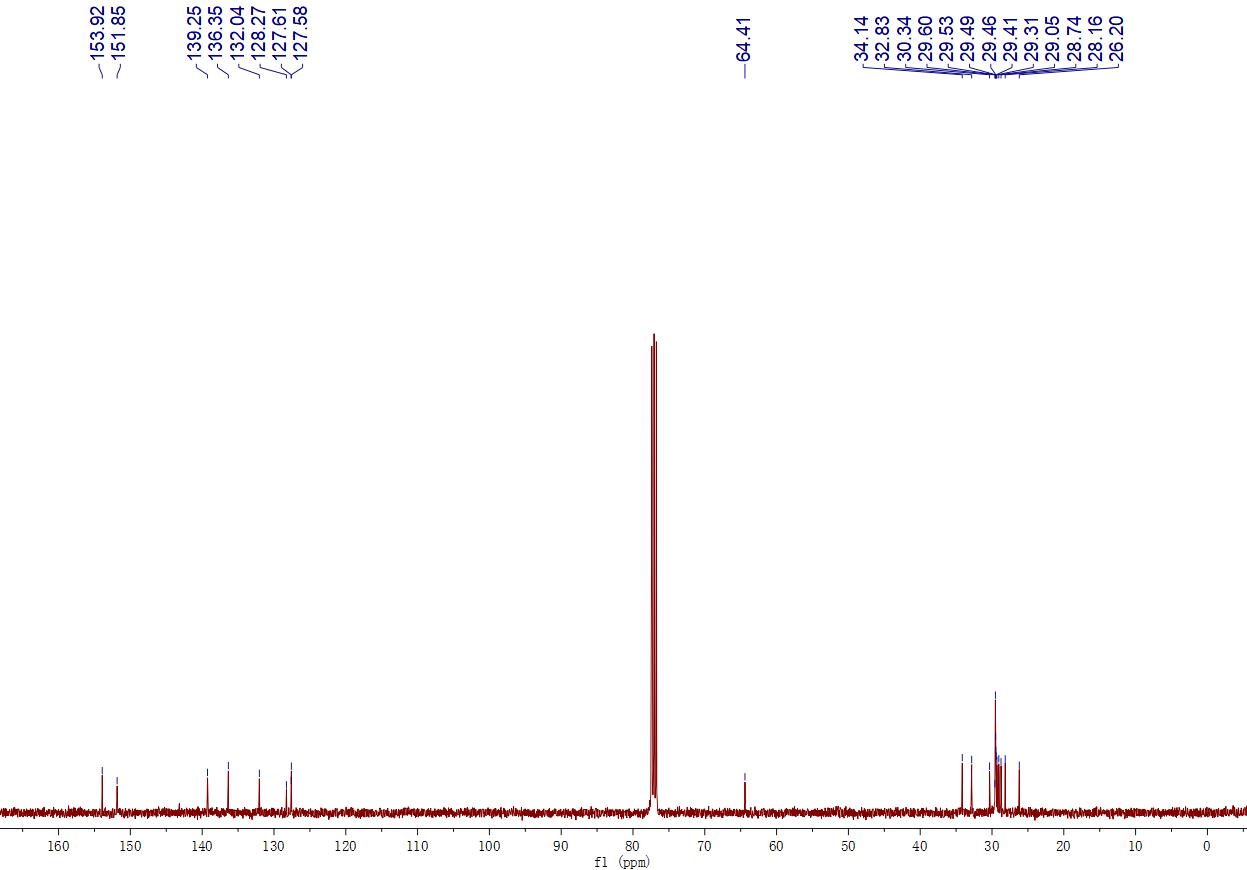

**Figure S22**. The NMR, HRMS and LC data of compound **2v**.
